# Site-specific bioorthogonal protein labelling by tetrazine ligation using endogenous β-amino acid dienophiles

**DOI:** 10.1038/s41557-023-01252-8

**Published:** 2023-07-03

**Authors:** Daniel Richter, Edgars Lakis, Jörn Piel

**Affiliations:** Institute of Microbiology, Eidgenössische Technische Hochschule (ETH) Zürich, Zürich, Switzerland

**Keywords:** Biocatalysis, Proteome, Chemical modification, Applied microbiology

## Abstract

The tetrazine ligation is an inverse electron-demand Diels–Alder reaction widely used for bioorthogonal modifications due to its versatility, site specificity and fast reaction kinetics. A major limitation has been the incorporation of dienophiles in biomolecules and organisms, which relies on externally added reagents. Available methods require the incorporation of tetrazine-reactive groups by enzyme-mediated ligations or unnatural amino acid incorporation. Here we report a tetrazine ligation strategy, termed TyrEx (tyramine excision) cycloaddition, permitting autonomous dienophile generation in bacteria. It utilizes a unique aminopyruvate unit introduced by post-translational protein splicing at a short tag. Tetrazine conjugation occurs rapidly with a rate constant of 0.625 (15) M^−1^ s^−1^ and was applied to produce a radiolabel chelator-modified Her2-binding Affibody and intracellular, fluorescently labelled cell division protein FtsZ. We anticipate the labelling strategy to be useful for intracellular studies of proteins, as a stable conjugation method for protein therapeutics, as well as other applications.

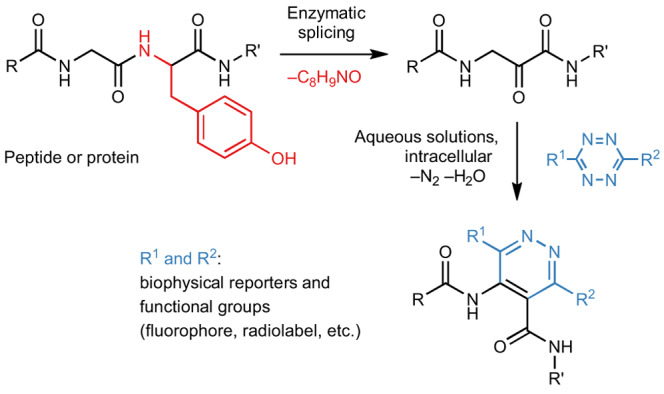

## Abstract (web summary)

An enzymatic reaction installs endogenous β-amino acids in proteins with unique reactivity. Now it has been shows that this reaction can be used for site-specific modification with tetrazine dienophiles to introduce labels onto target proteins. Applications include generation of a radiolabel chelator-modified Her2-binding Affibody and intracellular, fluorescently labelled cell division protein FtsZ.

## Main

Bioorthogonal chemistry has found widespread use in biological research and medicine by bringing rare chemical transformations into the diverse and crowded environment of living cells^1^. The rapid pace of innovation in the field has resulted in the development of numerous reactions that can be performed in biological settings. Site-specific chemical protein modifications have found many applications in biomedicine ranging from the incorporation of various biophysical probes^2^ to protein–drug conjugates^3^. Despite their utility, a number of drawbacks still exist. First, the bioconjugation of natural amino acids is often complicated by the presence of additional reactive sites in a protein. For instance, cysteine side-chain thiols have been used extensively in nucleophilic addition reactions; however, competing reactions with disulfide moieties or free thiols diminish the regioselectivity. Second, non-proteinogenic amino acids offer functionalities outside of the naturally occurring chemical space but can be challenging to incorporate into proteins. For example, carbonyl- and alkyne-functionalized amino acids have been used for their bioorthogonal reactivity with nucleophiles and azides, respectively^4–6^. Third, conjugation reactions are suitable for biological applications only if they are spontaneous, selective, fast and possible at low reagent concentrations, and many reactions developed in vitro are not suitable for in vivo applications^7^.

One of the most useful in vivo bioorthogonal transformations is the ligation of tetrazines with reactive strained dienophiles through an inverse electron-demand Diels–Alder (IEDDA) cycloaddition^8^^,^^9^. This rapid reaction is more selective than alternative bioorthogonal reactions, such as the Staudinger ligation^10^, copper-catalysed azide-alkyne cycloaddition^11^, strain-promoted azide-alkyne cycloaddition^12^ or variants of the Pictet–Spengler reaction^13^. A limitation of the tetrazine technology, however, is the incorporation of non-natural dienophiles into protein targets^14^. This is typically achieved by genetic code expansion^15^ or through enzyme-mediated ligation^16–20^, which require the addition of an external dienophile-carrying reagent. The de novo cellular production of a unique dienophile directly on the target protein would eliminate the need for external reagents, lessen the synthetic effort, and limit side reactivity and toxicity arising from substrate molecules and codon ambiguity.

**Fig. 1:**
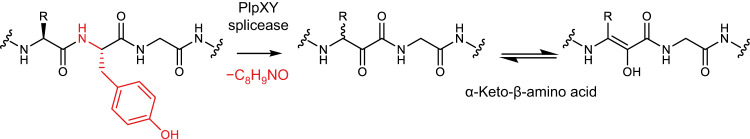
Net reaction of tyramine splicing. In the post-translational modification, the PlpXY splicease excises a tyramine equivalent (red, C_8_H_9_NO) at the tyrosine-glycine (YG) motif. R: amino acid side chain. The product is configurationally unstable at the former α-carbon due to enolization.

In this article, we leveraged a naturally occurring transformation in ribosomally synthesized and post-translationally modified peptide (RiPP) biosynthesis to accomplish facile in vivo installation of IEDDA sites. RiPPs are biosynthesized by post-translational maturation enzymes that act on ribosomally produced precursor peptides and install a wide range of non-canonical amino acids with diverse functionalities^21,22^. After maturation, a modified peptide is usually proteolytically cleaved and exported from the cell. We recently discovered an unusual bacterial RiPP modification that converts amino acids X within precursor XYG motifs to the corresponding ɑ-keto-β-amino acid residue, in the following referred to as ketoamide (Fig. 1)^23^.

This remarkable modification is achieved by the splicease complex PlpXY, of which PlpX belongs to the radical *S*-adenosylmethionine (rSAM) superfamily. It catalyses a formal net excision of a tyramine moiety from tyrosine and reconnection of the spliced peptide ends, thus fusing the remaining amide carbonyl of tyrosine to the carbonyl function of the preceding amino acid. The resulting homologized X residue, an α-keto-β-amino acid derivative, is predicted to possess unique chemical reactivity in the cellular proteome. Here we leveraged this peptide modification to achieve in vivo bioorthogonal IEDDA labelling of target proteins in a bacterial cell requiring only the external addition of a probe. While the previously discovered natural ketoamides carry side chains derived from non-glycine units, here we used an engineered GYG site to minimize steric hindrance during cycloaddition and enable a final aromatization step. We produced proteins containing the glycine-derived aminopyruvate residue in bacteria and site-selectively conjugated tetrazine probes using IEDDA cycloaddition. The utility of the technology was demonstrated by conjugating a Her2/ErbB2-binding Affibody with a dodecane tetraacetate chelator for use in cancer target radiolabelling and by fluorescently labelling the bacterial cell division protein FtsZ in fixed *Escherichia coli* cells.

## Results and discussion

### Reaction of aminopyruvate with tetrazines

Inspired by reports of tetrazine IEDDA cycloaddition to synthetic aldehydes^24^ and ketones^25^, we tested whether ketoamides also possess similar reactivity. We hypothesized that either the ketoamide enol form or an enamine spontaneously generated in the presence of biological amines could serve as the dienophile for IEDDA cycloaddition. Nevertheless, we did not find literature precedents where ketoamide functional moieties act as a dienophile.

**Fig. 2:**
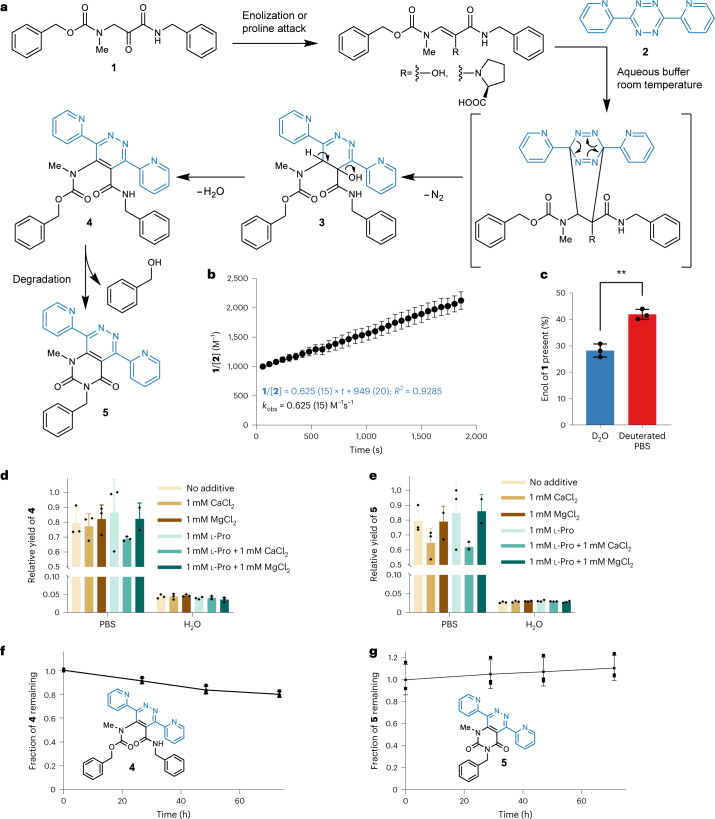
Test reactions of the aminopyruvate model compound 1 to explore the feasibility of pyridazine formation in vitro. **a**, Proposed reaction between **1** and 3,6-di-2-pyridyl-1,2,4,5-tetrazine **2** to yield intermediate **3**, and pyridazine products **4** and **5**. **b**, Reaction kinetics in PBS pH 7.4 measured by visible light spectrometry. The decrease in absorbance at 515 nm of tetrazine **2** was measured over time and fitted to a second-order reaction kinetic equation yielding an apparent rate constant of *k*_obs_ = 0.625 (15) M^−1^ s^−1^ in 30% DMSO in H_2_O (*n* = 3 independent experiments). Points describe mean; error bars describe standard deviation. **c**, Enol form of **1** present in solution in D_2_O or deuterated PBS (28.2% (2.4%) in D_2_O, 41.9% (1.9%) in PBS, *P* = 0.0016 by a two-tailed *t*-test) measured by deuterium exchange and MS (*n* = 3 independent experiments). Bars describe mean; error bars describe standard deviation. **d**,**e**, Effect of medium, l-proline and divalent metal ion additives on the yields of pyridazine **4** (**d**) and **5** (**e**), estimated by MS product ion abundance and relative product formation (*n* = 3 independent experiments). Bars describe mean; error bars describe standard deviation. **f**,**g**, Stability of conjugate **4** (**f**) and **5** (**g**) over time in 50% acetonitrile in H_2_O, assessed by UV signal (280 nm). The line connects mean values; error bars represent standard deviations (*n* = 3 independent experiments).

For model reactions we synthesized the aminopyruvate derivative **1** to study its IEDDA reactivity and the catalytic effect of l-proline and divalent metal ion additives. Synthetic compound **1** was incubated with 3,6-di-2-pyridyl-1,2,4,5-tetrazine (**2**) for 18 h in aqueous solution at 37 °C (Fig. 2a). To individual test reactions we added l-proline, Ca^2+^ or Mg^2+^. Product formation and the effect of additives on product yield was monitored by liquid chromatography–mass spectrometry (LC–MS) comparing apparent relative conversions by MS intensities (Fig. 2d,e). Model aminopyruvate IEDDA reactivity data showed pyridazine formation regardless of additives in aqueous conditions. Neither l-proline nor other additives resulted in substantially higher conversion in water. A 20-fold increase in yield, however, was observed in phosphate-buffered saline (PBS), a buffer that emulates in vivo conditions (Fig. 2d,e). The reported proline-catalysed IEDDA to synthetic aldehydes and ketones proceeds through the corresponding enamine intermediates; however, an enolization is probably sufficient for the aminopyruvate cycloaddition and might benefit from the presence of phosphate, which can act as enolization catalyst^26^. We observed accelerated enolization of **1** in PBS (*k*_obs_ = 1.05 × 10^−4^) compared with water (*k*_obs_ = 2.5 × 10^−7^) by deuterium exchange and LC–MS (Supplementary Fig. 22). Additionally, we observed an increased amount of enol present in PBS compared with D_2_O (Fig. 2c, 28.2% (2.4%) in D_2_O compared with 41.9% (1.9%) in PBS, *P* = 0.0016). The stability of conjugates **4** and **5** was analysed by LC (Fig. 2f,g) over 3 days, which showed less than 25% decrease in UV signal after 72 h.

Reaction kinetics were analysed in PBS by ultraviolet (UV)–visible spectrometry. The decrease of the absorption maximum at 515 nm for tetrazine **2** at 25 °C was recorded over 30 min (Fig. 2b). The acquired data follow a second-order reaction mechanism affording a second-order rate constant of 0.625 (15) M−^1^ s^−1^. For lower concentrations of tetrazine in solution we observed a systematic deviation from the rate law in the first 5 min of reaction progress (Supplementary Fig. 20e). This accelerated rate might indicate excess enol that is more rapidly consumed than the formation of enol. However, we were not able to test the reaction behaviour more carefully during the initial seconds to fully characterize this behaviour. We additionally tested the less electronically activated (4-(1,2,4,5-tetrazin-3-yl)phenyl)methanamine **8**, which showed a slower second-order rate constant of *k*_obs_ = 0.049 (9) M^−1^ s^−1^, although for this example we observed a faster rate constant in H_2_O of *k*_obs_ = 0.18 (5) M^−1^ s^−1^ (Supplementary Fig. 21). While these kinetic values are slower than conventional strained dienophiles, the reasonably fast kinetics of the model reaction encouraged us to test next whether it is possible to enzymatically incorporate the glycine-derived aminopyruvate moiety into proteins.

### Reactive site incorporation and in vitro conjugation

**Fig. 3:**
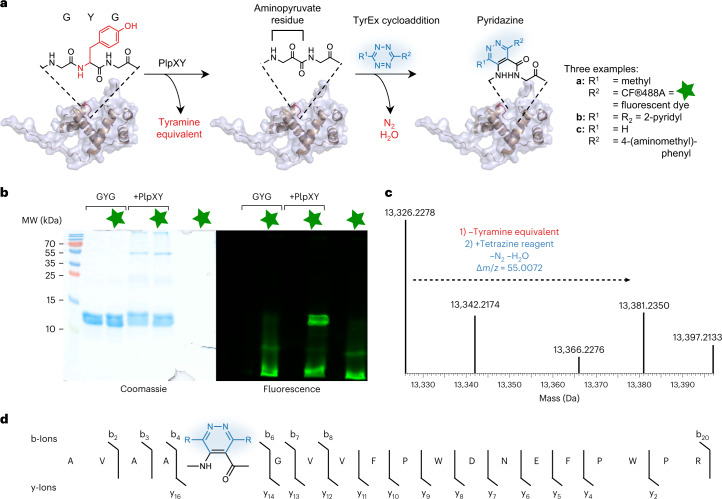
In vitro peptide conjugation of His_6_-PlpA3-M5G with tetrazines. **a**, Overall tyramine excision (TyrEx) strategy for labelling of His_6_-PlpA3-M5G: PlpXY expressed in the cell installs aminopyruvate site-specifically in the target biomolecule at a GYG-containing motif. Splicing results in a mass loss of −135.0757 Da (−C_8_H_9_NO). The post-translationally modified product reacts tetrazine derivatives to form the pyridazyl protein conjugates **a**–**c**. Structures are based on models calculated with AlphaFold^47,48^. **b**, Conjugation of His_6_-PlpA3-M5G with a fluorescent tetrazine probe analysed by SDS–PAGE. GYG: unmodified His_6_-PlpA3-M5G molecular weight (MW) 13.3 kDa. +PlpXY: His_6_-PlpA3-M5G was co-produced with PlpXY to yield the aminopyruvate-containing product (MW 13.2 kDa). The CF®488A fluorophore (**6**) was imaged by gel fluorescence using a 488 nm excitation and 532/528 nm emission filter. The experiment was performed once. **c**, Deconvoluted electrospray ionization–mass spectra (ESI–MS) showing ions for the substrate (His_6_-PlpA3-M5G, *m*/*z* calculated 13,326.2112, found 13,326.2278) and the cycloaddition product using tetrazine **2** (*m*/*z* calculated 13,381.2071, found 13,381.2350). **d**, Observed y- and b-ions for the ESI–MS/MS fragmentation of a trypsinized reaction product localizing the cycloaddition site (blue). Source data

As mentioned, ketoamide units are biologically generated by the post-translational machinery of spliceotides, a family of bacterial ribosomal peptides that we had discovered previously. For this study we tested the prototype splicease PlpXY from the cyanobacterium *Pleurocapsa* sp. PCC 7319, which naturally acts on MYG and LYG peptide sites to generate the corresponding ketoamides with methionine and leucine side chains. Studies on precursor analogues showed that a point mutant of the natural substrate PlpA3 with an engineered GYG motif is also accepted as a substrate and converted to the aminopyruvate-containing product lacking a ketoamide side chain^23^. The mutation to the sterically less demanding residue was hoped to facilitate tetrazine cycloadditions in complex cellular environments. To test whether IEDDA is compatible with a large peptide, an N-terminally His_6_-tagged variant of the GYG precursor mutant, His_6_-PlpA3-M5G (117 amino acids), was co-produced with the splicease PlpXY in *E. coli* by expression from the plasmids *plpA3-M5G*/pACYCDuet-1 and *plpXY*/pRSFDuet (Supplementary Table 2). Isolation of His_6_-PlpA3-M5G by Ni-nitrilotriacetic acid (NTA) affinity purification and subsequent in vitro conjugation with tetrazines verified successful conversion of the substrate to the aminopyruvate-containing product by PlpXY (Fig. 3 and Supplementary Figs. 24 and 25). Using the affinity-purified protein, we next investigated IEDDA reactions of spliced His_6_-PlpA3-M5G with a range of tetrazine reagents (Fig. 3a). Tetrazine **2** was added to the isolated protein in a phosphate-based buffer (50 mM NaH_2_PO_4_, 300 mM NaCl and 250 mM imidazole, pH 8.0) and incubated at 20 °C for 18 h, similar to experiments with the model compound in PBS. Protein conjugation with the tetrazine probe **2** was confirmed by MS analysis and subsequent mass deconvolution (product *m*/*z* calculated 13,381.2071; observed 13,381.2350 in Fig. 3c). Limited conversion is due to the incomplete enzymatic step during co-expression of substrate and PlpXY (Supplementary Fig. 24). Notably, in tandem mass spectrometry (MS/MS) analyses, b- and y-ions of the labelled peptide displayed a mass change of +55.0099 relative to the His_6_-PlpA3-M5G precursor, indicative of the incorporation of the tetrazine probe at the expected aminopyruvate site (Fig. 3d). A commercially available fluorescent tetrazine derivative, CF®488A (**6**), was also tested in IEDDA reactions with the aminopyruvate-containing His_6_-PlpA3-M5G, and similar results were obtained (Supplementary Fig. 31 and 32). Fluorophore incorporation was verified by gel electrophoresis followed by fluorescence imaging (Fig. 3b). By splicing the target protein to generate bioconjugation substrates in vivo and subsequent in vitro IEDDA cyclization, we were able to generate labelled His_6_-PlpA3-M5G after 18 h at mild conditions. We termed this method TyrEx (tyramine excision) cycloaddition.

### In vitro production of Her2-binding affibody conjugates

After establishing the TyrEx cycloaddition in the context of the natural splicease substrate, we asked whether it can be implemented for non-native target proteins unrelated to spliceotides or RiPPs. We previously established that ketoamide production does not require the RiPP leader. This permits the incorporation of ketoamide moieties into diverse proteins at C-, N-terminal and internal positions in *E. coli* by integrating a ten-residue splice tag^27^. Here we considered antigen-binding conjugates, which are widely used in targeted drug delivery^28^, positron emission tomography^29^ and near-infrared^30^ in vivo imaging, and for antigen inactivation by binding^31^. This therapeutic and diagnostic class of molecules can be divided into antibody-based (for example, antibody–drug conjugates, light and heavy chain fragments^32^, and nanobodies)^33^ and engineered non-antibody protein-binding scaffolds (for example, DARPins^34^ and Affibodies)^35^. While the first category is produced mainly in eukaryotic cells, the second can be readily produced and genetically manipulated in *E. coli*. We therefore selected a Her2/ErbB2-binding Affibody (Z_Her2:342_) as a model protein^36^. The α-helical Affibody (8.3 kDa) is highly water soluble and substantially smaller than antibody fragments (4×) or antibodies (20×). For diagnostic radiolabelling purposes, Affibodies are conjugated to ligands that bind radioactive metals, enabling tumour imaging in vivo. Usually, these conjugates are prepared through cysteine-maleimide addition with derivatives of 1,4,7,10-tetraazacyclododecane-1,4,7,10-tetraacetate (DOTA), utilizing a cysteine residue at the Affibody C-terminus^37^. Maleimide conjugations have been reported to suffer from hydrolytic cleavage and thiol exchange reactions in vivo^38,39^. We therefore envisioned that linking the Affibody by TyrEx addition would alleviate these shortcomings and require fewer preparatory steps than the thiol chemistry as no reduction step is needed and the reaction selectively proceeds in the presence of other biomolecules.

**Fig. 4:**
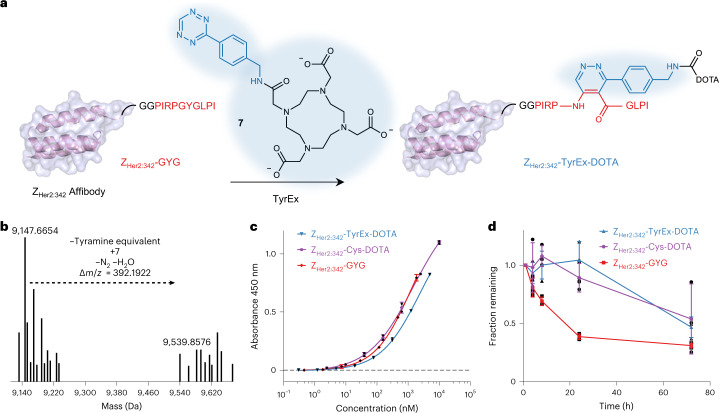
Production of a Her2-binding Affibody conjugate for radiolabelling by TyrEx cycloaddition. **a**, Scheme for generating the Z_Her2:342_-DOTA conjugate. Cartoon visualization of the Z_Her2:342_ Affibody based on crystal structures (PDB accession 3MZW)^49^ with the C-terminal splicease tag (in red, GG serves as a linker). In vivo splicing and subsequent in vitro conjugation to DOTA-tetrazine (**7**) yields conjugates for radiolabelling. **b**, Deconvoluted MS spectrum of Z_Her2:342_-GYG (*m*/*z* calculated 9,147.6540, found 9,147.6654) and the DOTA conjugate (Z_Her2:342_-TyrEx-DOTA, *m*/*z* calculated 9,539.8277, found 9,539.8576). **c**, ELISA to detect Affibody binding to the recombinant Her2 extracellular domain immobilized on a 96-well plate. There was no significant difference between Z_Her2:342_-TyrEx-DOTA and Z_Her2:342_-Cys-DOTA (*n* = 2 independent experiments, *P* = 0.3131 by a two-tailed *t*-test with Welch’s correction). **d**, Stability of the Z_Her2:342_-TyrEx-DOTA conjugate, its precursor protein, and Z_Her2:342_-Cys-DOTA in human blood plasma over 72 h at 37 °C, measured by LC–MS. The line connects mean values; error bars represent standard deviations (*n* = 3 independent experiments).

To prepare the dienophile substrate, we generated an N-terminally His_6_-tagged Affibody His_6_-Z_Her2:342_ hybrid with a C-terminally fused 10-amino-acid minimal PlpA2 core containing an engineered GYG motif (splice tag sequence in Fig. 4a). The protein, designated His_6_-Z_Her2:342_-GYG, was heterologous co-produced in *E. coli* together with the splicease PlpXY by expression from pACYC and pRSF vectors (Supplementary Table 2). Affinity purification and LC–MS/MS analysis suggested the presence of the reactive aminopyruvate residue (Supplementary Figs. 33 and 34). We performed the IEDDA reaction on spliced Z_Her2:342_-GYG with the DOTA tetrazine **7** (Fig. 4a). Affinity-purified aminopyruvate-containing His_6_-Z_Her2:342_-GYG was added with **7** in PBS buffer. Incubation at 37 °C for 18 h yielded the Affibody-DOTA conjugate (Z_Her2:342_-TyrEx-DOTA), which was confirmed by LC–MS analysis and subsequent mass deconvolution (product *m*/*z* calculated 9,539.8277; observed 9,539.8553 in Fig. 4b). MS/MS analysis confirmed localization of the modification to the expected site (Supplementary Figs. 35 and 36).

Z_Her2:342_-TyrEx-DOTA and the precursor protein Z_Her2:342_-GYG were purified by reversed-phase high-performance liquid chromatography (HPLC) and analysed by an enzyme-linked immunosorbent assay (ELISA) with Her2 immobilized on 96-well plates to assess whether binding functionality is preserved (Fig. 4c). For comparison, Z_Her2:342_ with a C-terminal cysteine was expressed from a pACYC vector in *E. coli* and conjugated with maleimide-DOTA as previously described to obtain Z_Her2:342_-Cys-DOTA^37^. The ELISA suggested comparable binding (*P* = 0.3131) affinities for Z_Her2:342_-TyrEx-DOTA (*K*_D,app_ = 2,348 nM) and Z_Her2:342_-Cys-DOTA (*K*_D,app_ = 1,432 nM).

To characterize the stability of the two conjugates, Z_Her2:342_-TyrEx-DOTA and Z_Her2:342_-Cys-DOTA as well as the precursor fusion protein Z_Her2:342_-GYG were incubated in human blood plasma for up to 3 days at 37 °C (Fig. 4d). While there was no significant difference between the two conjugates, the precursor tagged with the GGPIRPGYGLPI sequence was degraded very quickly with below 50% remaining after 24 h. MS analysis of the degradation products suggested that the proline-glycine bond N-terminal to the modification site is degraded (Supplementary Fig. 38). MS data for the TyrEx conjugate suggested the same degradation site (Supplementary Fig. 38). Therefore, we hypothesize that the detected degradation is not a consequence of the conjugation but of the peptide sequence itself, as the conjugated protein degraded more slowly than the unconjugated precursor (Fig. 4d). We hope to leverage any of the 27 other characterized or 939 predicted splicease systems reported by our group for future applications to avoid the issue of degradation^40^.

### Confocal imaging of labelled FtsZ in *E. coli* cells

**Fig. 5:**
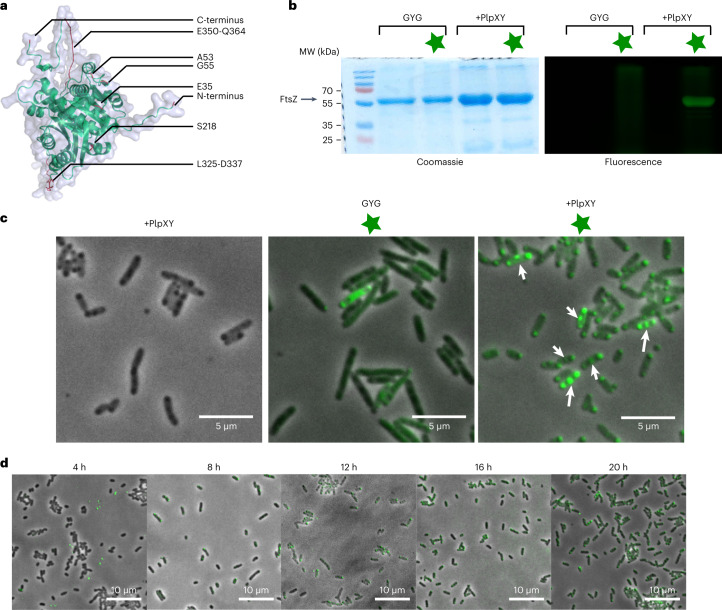
Imaging of FtsZ fluorescently labelled in vivo by TyrEx cycloaddition. **a**, Structure of FtsZ predicted with AlphaFold^47,48^. Studied tag locations are highlighted in red and labelled with black lines (see also Table 1). **b**, SDS–PAGE analysis of CF®488A (**6**) labelled FtsZ G55-Q56. The left gel shows Coomassie-stained protein bands; the right gel shows gel fluorescence using 488 nm excitation and 532/28 nm emission filter. Samples labelled +PlpXY were co-expressed with the splicease PlpXY. The star symbol denotes the addition of **6** (10 μM). The experiment was performed once. **c**, In situ fluorescence micrographs of fixed *E. coli* cells harbouring fluorescent FtsZ-G55-Q56. Fluorescent signals in the septum of cells are highlighted with white arrows. **d**, Fluorescence micrographs of cells cultured to the indicated time points after induction of protein expression. Overlaid images of brightfield illumination and 488 nm are shown. All representative microscopy images where manually selected from 3–12 images acquired for each condition. Source data

With protein bioconjugation established in vitro, further experiments aimed to establish intracellular labelling. As the cellular target, we selected the cell division protein FtsZ for fluorescence imaging. FtsZ is a polymeric cytoskeletal protein that during cell division localizes at the nascent septum of prokaryotic cells and thus guides cell wall formation^41,42^. Due to its focused localization, fluorescent variants can be distinguished readily from non-specific labelling, and the catalytic activity and inter-monomer interactions required for functional FtsZ were useful features to assess potential functional disturbances caused by the TyrEx modification that impair cell division. We probed the surface of *E. coli* FtsZ by inserting splice tag sequences at eight locations across the protein (Table 1 and Fig. 5a). Different splicease recognition motifs containing the GYG region were genetically introduced into His_6_-tagged FtsZ at either the N- or the C-terminus, or inserted at internal sites between amino acids G55-Q56, A53-V54, E35-G36 or S218-E219 that have been reported before as suitable for small peptide insertions^41,43^. In addition, 11- and 13-amino acid segments L325-D337 and E350-Q364, respectively, in the intrinsically disordered FtsZ ‘spring’ region were substituted with the splicease motif sequence.

**Table 1:** Tested splice tag sequences and locations in FtsZ

Insertion site	Tag peptide sequence	Modified
N-terminus	GGPIRPGYGLPI	−
	GAVAAGYGVVFPG	−
E35	GGPIRPGYGLPI	−
A53	GGPIRPLYGLPI	−
	GGPIRPGYGLPI	−
G55-Q56	GPIRPLYGLPI	−
	GPIRPGYGLPI	−
	GPIRPGYGLPILEGSTI	−
	IGSTLEGGPIRPGYGLPI	−
	IGSTLEGGPIRPGYGLPILEGSTI	+
S218	GGPIRPGYGLPI	−
L325-D337	FPIRPGYGLPI	−
	AVAGYGLPI	−
E350-Q364	PLFPIRPGYGLPI	+
C-terminus	GGPIRPGYGLPI	−
	GGAVAAGYGVVFP	−

‘−’ denotes FtsZ variants that were not accepted; ‘+’ denotes accepted substrates.

First, we tested production of aminopyruvate-modified FtsZ variants for the constructs by heterologous co-production with the PlpXY splicease in *E. coli*. His_6_-tagged target proteins were expressed from pACYCDuet-1 and pRM006 plasmids (the latter purchased from Addgene, #98922) and purified by Ni^2+^-affinity chromatography for LC–MS analysis. While most of the tested constructs yielded soluble FtsZ variants, only the two variants FtsZ-E350-Q364 and FtsZ-G55-Q56 were successfully spliced at the GYG site, as verified by LC–MS/MS analysis (Supplementary Figs. 39–42).

Subsequently, we conducted a test conjugation in vitro with the aminopyruvate-containing FtsZ variant G55-Q56 using a fluorescent CF488A® tetrazine probe (**6**) and assessed labelling by gel electrophoresis imaging (Fig. 5b). For the assays, the His_6_-FtsZ-G55-Q56 protein solution prepared by Ni^2+^-affinity chromatography was mixed with **6** (1 µM) and incubated for 18 h at 37 °C. The reaction mixtures were directly loaded on the polyacrylamide gel. Gel fluorescence imaging and Coomassie staining showed a distinct band at the expected molecular weight region for FtsZ, while no fluorescence was observed in a control experiment using non-spliced protein.

With suitable FtsZ variants in hand, we next interrogated intracellular protein splicing, labelling, and imaging in the same cells. An *E. coli* strain was prepared carrying the two expression plasmids *ftsZ-G55*/pACYCDuet-1 and *plpXY*/pRSFDuet for the target protein FtsZ-G55-Q56 and the PlpXY splicease, respectively. 20 h after induction of expression in TB medium, *E. coli* cells were fixed with formaldehyde and permeabilized with Triton X-100. Bacteria were resuspended, mixed with **6** (10 μM) and shaken for 1 h at 37 °C. Subsequently, the cells were washed and analysed for cell phenotype by confocal laser scanning microscopy. These analyses revealed selective staining only for assays containing the FtsZ variant, the splicease, and the dye (Fig. 5c). Fluorescence is localized in the septum in the Z-ring structure as well as on poles of dividing cells where FtsZ is sequestered in granules that form at the cell poles in stationary phase^42,44^. Fluorescence of the cytoskeletal structure was not observed in cells producing FtsZ G55-Q55 alone (Fig. 5c, middle micrograph). In a time course experiment, we observed that co-expression of FtsZ with PlpXY was needed to proceed for at least 12 h for sufficient enzyme activity (Fig. 5d). These results showed that the TyrEx bioconjugation can fluorescently label proteins in cells by introducing a short peptide tag, thus providing an attractive alternative to existing intracellular labelling methods.

## Conclusion

This study describes an additional tetrazine ligation strategy, termed TyrEx cycloaddition that is based on autonomous dienophile generation in a bacterial cell. The method utilizes a unique aminopyruvate unit incorporated into target proteins by enzymatic backbone splicing at a genetically introduced GYG site. IEDDA reactions between aminopyruvate residues and tetrazines probably proceed through the aminopyruvate enol form with a rate of ~0.625 M^−1^ s^−1^ in PBS buffer for the analysed model compound, slower than conventional strained dienophiles. The conjugate was shown to be stable over 72 h with no major degradation in vitro. The TyrEx cycloaddition was applied to site-specifically label the therapeutically relevant Her2/ErbB2 Affibody protein with the DOTA chelator for use in cancer target radiolabelling. In addition, the method allowed us to fluorescently label the cell division protein FtsZ in fixed *E. coli* cells via an inserted 24-residue sequence.

Several features of the TyrEx cycloaddition system make it attractive for protein conjugation. First, the protein splicing system is naturally not present in *E. coli* or mammalian cells, which makes the post-translational modification of target proteins bioorthogonal and genetically tractable. Second, the de novo cellular production of the dienophile directly on the target protein eliminates the need for incorporation of dienophiles by enzyme-mediated ligations or unnatural amino acid incorporation, often accompanied by low yields and interferences from codon reassignments. Third, the dienophile is located on the protein backbone, reducing the linker length to the label and effective size of the protein modification, making this method attractive for exact molecular distance measurements by Förster resonance energy transfer^45^ or electron paramagnetic resonance^46^. TyrEx conjugates can easily be prepared in few steps, lessen the synthetic effort, and limit side reactivity and toxicity arising from substrate molecules. Antigen-binding protein conjugates prepared through TyrEx thus provide an alternative strategy to existing methods that can alleviate shortcomings from other methods. Conjugates are functional and similarly stable in human blood plasma as other methods. In fluorescent protein labelling, the small size of the TyrEx tag (minimally ten amino acids) permits applications that are not compatible with the larger GFP-derived labels. However, the slower reaction kinetics can be limiting in some cases where the use of strained dienophiles will be more appropriate.

Regarding further optimization potential, FtsZ tested here required relatively elaborate tag optimization regarding length and location to achieve modification by PlpXY. In contrast, splicing has been more straightforward for five different other proteins in vitro using terminal as well as internal positions^27^. More processive spliceases (either natural homologues or engineered variants) and alternative and further shortened peptide tag sequences will further broaden the applicative use, as would an implementation of the method in eukaryotic cells. Efforts to develop such enhanced splicing systems are ongoing in our laboratory^40^.

In vivo production of dienophiles for ligation with tetrazines expands the scope of bioorthogonal reactions for chemical biology, and simultaneously highlights the utility of the vast enzyme diversity from natural product biosynthesis outside their native context. We anticipate the labelling strategy to be useful for in vivo studies of cellular proteins, as a stable conjugation method for therapeutic and diagnostic proteins, and many other applications.

## Methods

### General

All reagents were purchased from commercial sources and used as received. Solvents were procured from Sigma-Aldrich and used as received. Double-distilled water was obtained from a PURELAB Chorus system (ELGA Veolia). Oligonucleotides for molecular cloning were synthesized by Microsynth (Switzerland). Plasmids were purified with the NucleoSpin plasmid purification kit purchased from Macherey-Nagel (Germany), agarose gel purifications were carried out with the NucleoSpin Gel and PCR Clean‑up kit (Macherey-Nagel). Q5 Site-Directed Mutagenesis kit, restriction enzymes, T4 DNA ligase and Q5 DNA polymerases were purchased from New England Biolabs (NEB). Antibiotics (chloramphenicol, kanamycin and ampicillin) were purchased from Applichem. GluC endoprotease was purchased from NEB, and sequencing-grade trypsin endoprotease was purchased from Promega. Bacteria lysis was done on Qsonica Q700 sonicator equipped with either a 2 or 6 mm probe. Protino agarose Ni-NTA resin was purchased from Macherey-Nagel. Nuclear magnetic resonance (NMR) spectra were acquired on a Bruker 300 (AV III), Bruker 400 (Ascend, Ult) or Bruker 500 (Avn) instrument using TopSpin 3.5./4.1 (Bruker). ^1^H-NMR chemical shifts are reported in p.p.m. relative to SiMe_4_ (*δ* = 0) and were referenced internally with respect to residual protons in the solvent (*δ* = 7.26 for CDCl_3_, *δ* = 2.50 for dimethyl sulfoxide (DMSO)-d_6_). Coupling constants are reported in Hz. ^13^C-NMR chemical shifts are reported in p.p.m. relative to SiMe_4_ (*δ* = 0) and were referenced internally with respect to solvent signal (*δ* = 77.16 for CDCl_3_). Peak assignments are based on calculated chemical shift and multiplicity. Splitting patterns are reported as follows: singlet (s), doublet (d), triplet (t), quartet (q) and multiplet (m). LC–MS experiments were performed on a Dionex Ultimate 3000 UHPLC equipped with columns from Phenomenex and coupled to a mass spectrometer. Mass spectra were acquired on an LTQ Orbitrap XL or Q Exactive (Thermo Fisher Scientific) spectrometer by using heated electrospray ionization. Thin-layer chromatography was performed using commercial Merck silica gel plates, and components were visualized with UV (*λ* = 254 nm). Model structures were predicted using the online Colab version of AlphaFold^47,48,50^. IUPAC names of all compounds are provided and were determined using CS ChemDraw Professional 18.0. LC–MS data were analysed with the Thermo Xcalibur Qual browser 4.1 (Thermo Fisher Scientific). For vector graphic creation, the open-source software Inkscape 0.92 was used.

### Plasmid construction

All plasmids used in this study are listed in Supplementary Table 2. Protein expression plasmids were constructed by Gibson Assembly Cloning (for genes >100 base pairs) or the Q5® site-directed mutagenesis (for genes <100 base pairs) protocol provided by NEB. Gene assembly fragments were designed by NEBuilder tool online. Overlapping mutagenic primers were designed by NEBaseChanger tool online. A typical polymerase chain reaction (PCR) (50 µl) contained 20 ng template DNA, 1× Q5 reaction buffer, 200 µM dNTPs, 0.5 µM of each primer (Supplementary Table 1) and 0.5 U Q5 High-Fidelity DNA Hot-Start Polymerase. The reaction was heated to 98 °C for 45 s followed by 35 cycles of 98 °C for 10 s, *X* °C for 20 s, and 72 °C for 20 s per kilobase DNA target sequence. The primer annealing temperature *X* was calculated with the NEB Tm calculator website or the NEBaseChanger (in case of the Q5 site-directed mutagenesis protocol). The resulting PCR amplicons were treated with kinase-ligase-DpnI mix (KLD mix, NEB). Gibson Assembly was performed with Gibson Assembly Master Mix (NEB) according to the manufacturer’s instructions. DNA was visualized by 1% (w/v) agarose gel electrophoresis supplemented with ethidium bromide and GeneRuler 1 kb DNA ladder (Thermo Scientific) marker. Plasmids were transformed into chemically competent *E. coli* DH5α strain (Invitrogen) and grown on LB agar containing appropriate antibiotics, then inoculated in corresponding liquid LB medium. Plasmids were isolated from fresh overnight cultures, and the gene of interest was sequence-verified by Microsynth. All plasmids are under isopropyl-β-d-1-thiogalactopyranoside regulation. pACYCDuet encodes chloramphenicol resistance, pRSFDuet kanamycin resistance and pRM006 ampicillin resistance.

### Small-molecule studies

For additive studies, model compound **1** (100 μM) was dissolved H_2_O or PBS and added with 3,6-di-2-pyridyl-1,2,4,5-tetrazine **2** (100 μM), then supplied with additives l-proline (50 µM), CaCl_2_ (1 mM) and/or MgCl_2_ (1 mM). The samples were incubated overnight at 37 °C and analysed by LC–MS. Relative product formation of **4** and **5** was analysed by comparing area under the curve for the respective masses in the chromatogram, normalizing to the highest conversion rate. From the MS data, the area under the curve for *m*/*z* = 531.2139 (for **4**) or *m*/*z* = 423.1564 (for **5**) were extracted. Data analysis and statistical analysis were done in Microsoft Excel (2016) and Prism 9 (GraphPad).

### Stability measurements

Stability of **4** and **5** in aqueous solutions was measured by dissolving purified **4** or **5** in 50% acetonitrile in H_2_O to obtain 10 mM solutions. From these samples, 5 µl was then analysed every 24 h by LC–MS with method B over the course of 3 days, incubating at room temperature in between. Areas under the curve for UV traces (280 nm) were plotted against time. Measurements were done in triplicates.

### Kinetic measurements

For kinetic measurements, 140 µl H_2_O or PBS were added with 20 µl of a 10 mM stock solution of **1** in DMSO (final concentration 1 mM) and 20 µl, 40 µl or 80 µl of a 5 mM stock solution of **2** in DMSO (final concentrations: 0.5 mM, 1 mM or 2 mM) in a 96-well plate. Wells were filled up to 200 µl with DMSO (final mixture: H_2_O/DMSO: 70/30). Alternatively 10 µl, 20 µl or 40 µl of a 10 mM stock solution of (4-(1,2,4,5-tetrazin-3-yl)phenyl)methanamine hydrochloride (compound **8**) in DMSO was added and filled up to 200 µl with DMSO. Absorbance at 515 nm was monitored over time every minute on a BioTek Synergy H1 plate reader (Agilent). Before every measurement, samples were shaken for 1 s. Measurements were done in triplicates. Control samples without **1**, **2** or **8** were prepared by using the appropriate amount of DMSO. For each sample the signal change of just **1** in H_2_O was subtracted and the absorbance converted to a concentration of **2** or **8**. The reciprocal concentration of remaining tetrazine reagent versus time were plotted to yield a second-order rate constant through linear regression. Data analysis and statistical testes were done in Microsoft Excel (2016) and Prism 9 (GraphPad).

### Enolization studies

To determine the amount of enol present and *k*_obs_ for the enolization of **1** in solution, a 10 mM stock solution of **1** in DMSO was diluted with either H_2_O, D_2_O or deuterated PBS 1:100 (100 µM final concentration). Samples were then directly injected on a Dionex Ultimate 3000 coupled to a Q Exactive mass spectrometer. Samples were then re-injected every 30 min to measure the change in signal of **1** versus deuterated **1** or doubly deuterated **1**. The remaining fraction of **1** was then plotted to yield a half life of **1** towards the enolization reaction and the corresponding rate constant. The fraction of **1** observed in the very first measurement was assumed to yield the initial amount of enol present in either D_2_O or PBS due to the enol exchanging its proton faster than the keto form. Kinetic isotope effects were not considered in this study. Measurements were done in triplicates and statistical tests done in Prism 9 (GraphPad).

### Affibody-splicease motif fusion cloning

Protein expression plasmid *his*_*6*_*-z*_*Her2:342*_*-gyg*/pACYCDuet-1 encoding His_6_-Z_Her2:342_-GYG was generated by Gibson assembly. The *z*_*Her2:342*_*-gyg* gene was cloned into the multiple cloning site 1 of the pACYCDuet-1 vector in frame with the N-terminal His_6_-tag. The assembly PCR was carried out with overlapping Her2:342_UP and Her2:342_DOWN synthetic oligonucleotides (Supplementary Table 1) and pACYCDuet-1 as template DNA. The plasmid *his*_*6*_*-z*_*Her2:342*_*-cys*/pACYCDuet-1 encoding His_6_-Z_Her2:342_-Cys was constructed by full plasmid amplification with *his*_*6*_*-z*_*Her2:342*_*-gyg/*pACYCDuet-1 as template and mutagenic primers (Supplementary Table 1).

### FtsZ-splicease motif fusion cloning

The protein expression plasmid *ftsZ*/pRM006 encoding His_6_-SUMO tagged FtsZ was purchased from Addgene (#98922). The *ftsZ*/pRM006 was used as a template DNA for subsequently generating plasmids for expressing FtsZ fusions with the splice tag at the N-terminus and between E35-G35, A53-V54 and G55-Q56. These constructs were made by full plasmid amplification with mutagenic primers named after the corresponding plasmid constructs (Supplementary Table 1).

The FtsZ gene was subcloned also into pACYCDuet-1 vector, multiple cloning site 1 in frame with N-teminal His_6_-tag by Gibson assembly. The linear FtsZ gene was PCR amplified from *ftsZ*/pRM006 as the template with overlapping primers FtsZ gene_fwd, FtsZ gene_rev. The pACYCDuet-1 vector was used as a template for full plasmid amplification by PCR using pACYCDuet1_fwd and pACYCDuet1_rev primers. Primers were designed in NEBuilder tool online. Assembly of PCR products yielded *ftsZ*/pACYCDuet-1 plasmid, which was used as a template DNA for splice tag insertion by Q5 site-directed mutagenesis. The splicing motif was inserted between L325-D337, E350-Q364 and at N- and C-terminus of FtsZ. The same name was given to primers and plasmids as for the corresponding insertion site (Supplementary Table 1).

### Protein expression and purification

A Falcon tube containing 5 ml of LB medium was inoculated with *E. coli* BL21 (DE3) cells taken from previously prepared glycerol stocks or from single colonies on agar plates and supplemented with the appropriate antibiotics. The culture was shaken at 180 to 220 r.p.m. overnight at 37 °C.

On the next day, 30–800 ml TB medium containing the appropriate antibiotics was inoculated with 1% v/v of this overnight culture and shaken at 37 °C until an OD_600_ of 1.4–1.8 was reached, according to previously reported expressions in similar systems^23^. After cooling the cultures at 4 °C for at least 20 min, 1 mM of isopropyl-β-d-1-thiogalactopyranoside was added and the cultures were incubated on a shaker at 180–220 r.p.m. at 16–24 °C for approximately 16–20 h. Subsequently, the cultures were centrifuged at 6,000*g* for 10 min at 4 °C. The supernatant was discarded, and the cell pellets resuspended in 1–25 ml NPI-10 buffer. All NPI buffers were supplemented with 10% glycerol.

All volumes were adjusted to scale with the culture volume (for example, 1 ml NPI-10 buffer for resuspension was used for 30 ml cultures, and 10 ml NPI-10 buffer were used for resuspension of cells grown in 300 ml medium). NPI buffers contain 50 mM NaH_2_PO_4_, 300 mM NaCl and 10–250 mM imidazole and are adjusted to pH 8.0.

Cells resuspended in NPI-10 buffer were sonicated for 10 times 10 s, with 10 s of pause in between, at an amplitude of 25–40. A sonication tip with a radius of 2–6 mm was used, depending on the volume to be sonicated. The resulting suspensions were kept on ice and centrifuged at 21,000*g* for 30 min at 4 °C.

The supernatant was transferred to a new tube and 125 µl to 2 ml of Protino Ni-NTA Agarose (Macherey-Nagel) was added. The samples were slowly shaken on a rotor at 10 r.p.m. for at least 30 min. An appropriate column was pre-washed with NPI-10 buffer, the sample added, washed twice with NPI-10 (500 µl to 10 ml), twice with NPI-20 (500 µl to 10 ml) and the protein eluted with NPI-250 (550 µl to 10 ml) and collected. Elution fractions were digested with appropriate endoproteinases. Protein splicing was analysed by high-resolution LC–MS. Samples were stored at −20 °C for further use.

### General protein labelling reactions

Ni-NTA affinity-purified proteins containing aminopyruvate residues were generally incubated in NPI-250 buffer with 100 µM (**2** and **7**) or 1–10 µM (**6**) for 18 h at 37 °C. Reactions were then directly analysed by LC–MS for full proteins or first digested with appropriate endoproteases.

Proteins labelled with a tetrazine probe conjugated to a fluorescent dye (CF®488A tetrazine **6**, Chemie Brunschwig AG) were run on sodium dodecyl sulfate polyacrylamide gel electrophoresis (SDS–PAGE) after incubation overnight at 37 °C. The gels were analysed by a fluorescent imager with a band pass filter 532/28 (ChemiDoc MP, Bio-Rad), followed by staining with Coomassie Brilliant Blue and subsequent destaining (10% AcOH, 30% MeOH and 60% H_2_O) overnight.

### Z_Her2:342_-TyrEx-DOTA and Z_Her2:342_-Cys-DOTA conjugation and purification

The Z_Her2:342_-TyrEx-DOTA conjugation reaction was prepared by adding DOTA-tetrazine **7** (10 μl, 1 mM solution in DMSO) to the splice-tagged protein solutions in NPI-250 buffer. Following incubation overnight at 37 °C, reaction mixtures were loaded to solid phase extraction columns (Strata-X 100 mg/3 ml, Phenomenex) pre-equilibrated first with 10 column volumes acetonitrile and subsequently with 10 column volumes H_2_O. Columns were washed with 10 column volumes 5% acetonitrile/H_2_O + 0.1% formic acid. Protein conjugates were eluted with 10 column volumes 30% acetonitrile/H_2_O + 0.1% formic acid. Resulting elutions were purified by reversed-phase HPLC on a Phenomenex Luna 5 µm Phenyl-Hexyl column (250 × 4.6 mm) with solvent A: H_2_O + 0.1% trifluoroacetic acid, solvent B: acetonitrile + 0.1% trifluoroacetic acid by the following gradient method: B 5% ramped up to 25% over the first 2 min, then a slow gradient to 29% at 14 min, then to 100% B over 1 min, 100% B for 2 min, then again 5% B for three more minutes to equilibrate. Fractions were analysed by LC–MS for purity and pure fractions combined.

Z_Her2:342_-Cys-DOTA^37^ was prepared from Ni-NTA elutions (2 mg ml^−1^) by first reducing the protein solutions with 30 µM dithiothreitol for 2 h at 37 °C and removal of excess dithiothreitol by PD-10 desalting columns pre-equilibrated with 20 mM ascorbic acid. Then 900 µl of the resulting elution was mixed with DOTA maleimide (200 µl, 1 mg ml^−1^) and NH_4_OAc (300 µl, 1 M, pH 5.5). The reaction mixtures were incubated overnight at 37 °C, desalted by PD-10 desalting columns pre-equilibrated with 200 mM NH_4_OAc, then purified by reversed-phase HPLC with the same conditions as Z_Her2:342_-TyrEx-DOTA.

Purified proteins were dried on a small-scale rotary evaporator (Eppendorf Concentrator 5301) and resuspended in PBS. Protein concentrations were measured by ROTI Nanoquant assay (Carl Roth).

### ELISA

Z_Her2:342_-TyrEx-DOTA (5 µM), Z_Her2:342_-GYG (1.875 µM), Z_Her2:342_-Cys (5 µM), Z_Her2:342_-TyrEx-Cys-DOTA (10 µM) and Her2 commercial Affibody (5 µM) were prepared as solutions in PBS at the given concentrations. The assay was carried out with a Her2-coated plate and buffers from an *EDI*^Tm^ Humanized Anti-Her2/neu (Herceptin/trastuzumab) ELISA Kit (Epitope Diganostics) following the manufacturer’s protocol. A 4× dilution series of each sample in 200 µl was prepared in duplicates in a 96-well transfer plate with the supplied assay buffer. Samples (100 µl) were then transferred to the Her2-coated plate and incubated at 22 °C on an orbital shaker at 400 r.p.m. for 90 min. Each well was washed five times with the supplied wash buffer, and 100 µl Goat Anti-Affibody IgG (Affibody SE) 0.5 µg ml^−1^ in assay buffer was added to each well, then incubated at 22 °C at 400 r.p.m. for 60 min. Each well was washed five times with the supplied wash buffer and 100 µl anti-goat IgG (Fc specific)-peroxidase antibody (Sigma-Aldrich), 50 ng ml^−1^ in assay buffer was added to each well, and the plate was then incubated at 22 °C at 400 r.p.m. for 60 min. Each well was washed five times with wash buffer, 100 µl of the supplied horseradish peroxidase substrate solution was added to each well, and the plate was wrapped with aluminium foil and incubated at room temperature in the dark for 30 min. The reaction was stopped by the addition of 100 µl of the supplied stop solution. Absorption at 450 nm was measured with a Victor3 (PerkinElmer) spectrophotometer. Binding curves and sigmoidal fits were constructed in GraphPad Prism 9.

### Blood plasma stability

Z_Her2:342_-TyrEx-DOTA, Z_Her2:342_-GYG, and Z_Her2:342_-Cys-DOTA (135 µg ml^−1^, 25 µl) and 25 µl human blood plasma (Sigma-Aldrich) were mixed and incubated at 37 °C in triplicates. After 0, 1, 4, 8, 24 and 72 h, 10 µl was removed from the solutions and stored at −80 °C until LC–MS analysis. Samples were mixed with 25 µl of 75% acetonitrile in water to precipitate blood plasma proteins. Samples were subjected to LC–MS after centrifugation (2 × 20,000*g* for 10 min). Intensities of deconvoluted mass ions for Z_Her2:342_-TyrEx-DOTA, Z_Her2:342_-GYG and Z_Her2:342_-Cys-DOTA respective conjugates were recorded. Intensities were normalized to the *t* = 1 h timepoint and plotted against incubation time.

### Labelling proteins with a fluorescent tetrazine in *E. coli* cells

*E. coli* BL21 (DE3) cells harbouring precursor and splicease plasmids were grown according to the protein expression procedure. Cells were taken from the culture and diluted to an OD_600_ of 0.4–0.6 with PBS (pH 7.4, final volume: 3 ml). The samples were centrifuged (7,000*g*, 5 min), the supernatant discarded and the cells resuspended in PBS (3 ml). Samples were again centrifuged and resupended in PBS containing 0.3% Triton X-100 and 100 µM CF®488A tetrazine **6**. Samples were incubated for 60 min at 16 °C, then then centrifuged (7,000*g*, 5 min), the supernatant discarded and the cells resuspended in PBS (1 ml). This wash was repeated once. Samples were then lysed by sonication and purified according to the general Ni-NTA affinity purification protocol. Elution fractions were concentrated on Vivaspin 500 concentrators (molecular weight cut-off 3 kDa) and were run on SDS–PAGE. The gels were analysed by a fluorescent imager with a band pass filter 532/528 (ChemiDoc MP, Bio-Rad), followed by staining with Coomassie Brilliant Blue and subsequent destaining (10% AcOH, 30% MeOH and 60% H_2_O) overnight.

### Labelling proteins with a fluorescent tetrazine in *E. coli* cells and preparation for microscopy

*E. coli* BL21 (DE3) cells harbouring precursor and splicease plasmids were grown according to the protein expression procedure. Cells were taken from the culture and diluted to an OD_600_ of 0.4–0.6 with PBS (pH 7.4, final volume: 100 µl). The samples were centrifuged (7,000*g*, 5 min), the supernatant discarded and the cells resuspended in PBS (100 µl). Samples were incubated with 4% formaldehyde for 30 min, then washed twice with PBS. Samples were incubated with 0.3% Triton X-100 at room temperature for 30 min, then washed twice with PBS. Samples were incubated with 100 µM CF®488A tetrazine for 60 min at 16 °C, then centrifuged (7,000*g*, 5 min), the supernatant discarded and the cells resuspended in PBS (100 µl). This wash was repeated twice. Samples (1–3 µl) were then added to a cover slip and covered with 1.5% w/v agarose pad for imaging. From these samples, regions of interest were located using the brightfield mode on the microscope, and *z*-stacks were recorded in brightfield and at 488 nm (200 mW, 30% power, 400 ms).

Samples were imaged on an Axiovert 200 m (inverse) microscope, equipped with a Yokogawa CSU-X1 spinning-disk confocal unit and a LUDL BioPrecision2 stage with Piezo Focus. Images were acquired with an sCMOS camera (Orca Flash 4.0 V). Diode-pumped solid-state lasers were used as light sources, where the 488 nm (200 mW) laser was used. A 100× 1.4 CFI Plan Apo Oil objective was used for all image acquisitions. Emitted light was filtered using the GFP (ET 525/550) filter. The microscope was operated using the VisiVIEW (Metamorph) software. Image analysis was performed with Fiji (ImageJ).

### High-resolution MS

Mass spectra were acquired on an LTQ Orbitrap XL or Q Exactive (Thermo Fisher Scientific) spectrometer by using heated electrospray ionization. The following method was used for analysis on LC–MS:

Solvent A, H_2_O + 0.1% formic acid; solvent B, MeCN + 0.1% formic acid; column, Phenomenex Kinetex 2.6 μm C18-XB 100 Å (150 × 4.6 mm); flow rate, 1.0 ml min^−1^; gradient: 95:5 A/B for 0.5 min ramped to 5:95 A/B over 20 min).

For MS/MS analysis a normalized collision energy of 15–28 was used, depending on the observed fragmentation properties of peptide fragments. The MS was operated in positive ionization mode at a scan range of 150–2,000 *m*/*z*, automatic gain control target 2 × 10^5^, maximum injection time 100 ms and a resolution of 70,000 at 400 *m*/*z*. The spray voltage was set to 5.0 kV, probe heater temperature to 475 °C and capillary temperature to 270 °C. Columns were heated to 50 °C for methods A and C.

### Reporting summary

Further information on research design is available in the Nature Portfolio Reporting Summary linked to this article.

## Online content

Any methods, additional references, Nature Portfolio reporting summaries, source data, extended data, supplementary information, acknowledgements, peer review information; details of author contributions and competing interests; and statements of data and code availability are available at 10.1038/s41557-023-01252-8.

### Supplementary information

Supplementary Information Synthetic procedures and Supplementary Figs. 1–42 and Tables 1–3.

Reporting Summary

Supplementary Table 1 Raw source data for Figs. 2c–g and 4c,d and Supplementary Figs. 20b–g, 21b–g and 22b.

### Source data

Source Data Fig. 3 Unprocessed gel scans.

Source Data Fig. 5 Unprocessed + cropped section of microscopy images, and unprocessed gel scans.

## Data Availability

The data supporting the findings of this study are available in this article and Supplementary Information. The crystal structure of the affibody Z_Her2:342_ was downloaded from the RCSB PDB (PDB 3MZW). Source data are provided with this paper.
