# Supplementary material for: Site-specific bioorthogonal protein labelling by tetrazine ligation using endogenous β-amino acid dienophiles
Source: Nat Chem. 2023 Jul 3;15(10):1422–30. doi: 10.1038/s41557-023-01252-8 (PMC10533398; doi:10.1038/s41557-023-01252-8)
Supplement: Supplementary file 1 — Synthetic procedures and Supplementary Figs. 1–42 and Tables 1–3. [file 41557_2023_1252_MOESM1_ESM.pdf]

nature chemistry

Article

https://doi.org/10.1038/s41557-023-01252-8

Site-specific bioorthogonal protein labelling by tetrazine ligation using endogenous β-amino acid dienophiles

In the format provided by the authors and unedited

Table of contents

Synthetic procedures 2

Supplementary figures and tables 6

List of Supplementary Figures

Supplementary Figure	Figure Title	Page
**1**	Full gel images for PlpA3-M5G	6
**2**	Full gel images for FtsZ	6
**3**	Full gel images for FtsZ with co-expression of PlpXY in E. coli stained with CF®488A Tetrazine	7
**4**	Gel images for FtsZ with co-expression of PlpXY in *E. coli* stained with CF®488A Tetrazine	8
**5**	Full confocal microscopy images for FtsZ-G55	9
**6**	Full confocal microscopy images for FtsZ-G55 with co-expression of PlpXY (time course)	10
**7**	^{1}H-NMR (400 MHz) spectrum of aminopyruvate model compound **1** in $CDCl_3$	10
**8**	^{13}C-NMR (126 MHz) spectrum of aminopyruvate model compund **1** in $CDCl_3$.	11
**9**	^{1}H-^{1}H-COSY NMR (500 MHz) spectrum of aminopyruvate model compound **1** in $CDCl_3$.	12
**10**	HSQC (500 MHz) spectrum of aminopyruvate model compound **1** in $CDCl_3$.	13
**11**	^{1}H-NMR (400 MHz) spectrum of **3** in $CDCl_3$.	13
**12**	^{1}H-^{1}H-COSY (400 MHz) spectrum of **3** in $CDCl_3$.	14
**13**	HSQC (400 MHz) spectrum of **3** in $CDCl_3$.	14
**14**	^{1}H-NMR (400 MHz) spectrum of **5** in $CDCl_3$.	15
**15**	^{13}C-NMR (101 MHz) spectrum of **5** in $CDCl_3$.	15
**16**	HSQC (400 MHz) spectrum of **5** in $CDCl_3$.	16
**17**	^{1}H-^{1}H-COSY-NMR (400 MHz) spectrum of **5** in $CDCl_3$.	16
**18**	HMBC (400 MHz) spectrum of **5** in $CDCl_3$.	17
**19**	^{1}H-NMR (400 MHz) spectrum of DOTA-Tetrazine **7** in DMSO-d_6.	18
**20**	Kinetic measurements for the reaction between model compound **1** and 3,6,-di-2-pyridyl-1,2,4,5-tetrazine **2**	19
**21**	Kinetic measurements for the reaction between model compound **1** and (4-(1,2,4,5-tetrazin-3-yl)phenyl)methanamine hydrochloride **8**	20
**22**	Enolization kinetics of **1**	21
**23**	Cycloaddition of **1** and **2**, forming compounds **3**, **4**, and **5.**	22
**24**	LC-MS analysis for His_6-PlpA3-M5G cleaved with trypsin	23
**25**	Localization of C_8H_8NO loss from His_6-PlpA3-M5G cleaved with trypsin	24
**26**	LC-MS analysis for conjugation of tetrazine **2** with His_6-PlpA3-M5G cleaved with trypsin	25
**27**	Localization of pyridazine formation on His_6-PlpA3-M5G (with tetrazine **2**) cleaved with trypsin	26
**28**	LC-MS analysis of tetrazine **2** with His_6-PlpA3-M5G	27
**29**	LC-MS analysis for conjugation of 4-(1,2,4,5-tetrazin-3-yl)phenyl)methanamine **8** with His_6-PlpA3-M5G cleaved with trypsin	28
**30**	Localization of pyridazine formation on His_6-PlpA3-M5G (with 4-(1,2,4,5-tetrazin-3-yl)phenyl)methanamine) cleaved with trypsin	29
**31**	LC-MS analysis for conjugation of CF®488A tetrazine **6** with His_6-PlpA3-M5G cleaved with trypsin	30

**32**	Localization of pyridazine formation on His$_6$-PlpA3-M5G (with CF®488A tetrazine **6**) cleaved with trypsin	31
**33**	LC-MS analysis for $Z_{Her2:342}$-GYG cleaved with trypsin	32
**34**	Localization of C_8H_8NO loss from $Z_{Her2:342}$-GYG cleaved with trypsin	33
**35**	LC-MS analysis for conjugation of DOTA-tetrazine **7** with $Z_{Her2:342}$-GYG cleaved with trypsin	34
**36**	Localization of pyridazine formation on $Z_{Her2:342}$-GYG (with DOTA-tetrazine **7**) cleaved with trypsin	35
**37**	LC-MS analysis of DOTA-tetrazine **7** with $Z_{Her2:342}$-GYG	36
**38**	LC-MS analysis of degradation of $Z_{Her2:342}$-GYG and $Z_{Her2:342}$-GYG-DOTA	37
**39**	LC-MS analysis for FtsZ-G55-Q56 cleaved with trypsin	38
**40**	Localization of C_8H_8NO loss from FtsZ-G55-Q56 cleaved with trypsin	39
**41**	LC-MS analysis for FtsZ-E350-Q364 cleaved with trypsin	40
**42**	Localization of C_8H_8NO loss from FtsZ-E350-Q364 cleaved with trypsin	41

Synthetic procedures

Benzyl (2-hydroxyethyl)(methyl)carbamate (S1)

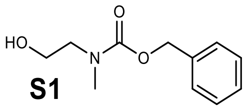

S1

To a solution of 2-(methylamino)ethan-1-ol (1.0 mL, 13 mmol, 1 eq.) and triethylamine (2.5 mL, 18 mmol, 1.4 eq.) in anhydrous dichloromethane (DCM) (4 mL) at 0 °C was added benzyl carbonochloridate (2.0 mL, 14 mmol, 1.05 eq.) over 30 minutes under an inert atmosphere. The reaction mixture was allowed to warm up to room temperature and stirred for 2 hours. Additional DCM (4 mL) was added and the organic phase was washed with 1 M HCl (8 mL). The aqueous phase was back-extracted with DCM (8 mL). The combined organic phases were washed with H_2O (8 mL) and brine (8 mL), dried over magnesium sulfate, filtered, and evaporated under reduced pressure to provide **S1** as a brown oil (2.085 mg, 77%).

Benzyl methyl(2-oxoethyl)carbamate (S2)

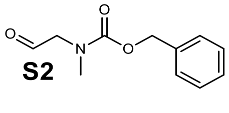

S2

After stirring a solution of **S1** (500 mg, 2.4 mmol, 1 eq.), dimethyl sulfoxide (DMSO) (1.7 mL, 24.0 mmol, 10 eq.), and *N,N*-diisopropylethylamine (DIPEA) (2.0 mL, 11.5 mmol, 5 eq.) at 0 °C for 10 minutes under an inert atmosphere, sulfur trioxide pyridine complex (1.9 g, 11.5 mmol, 5 eq.) was added and the mixture was stirred for 1.5 hours. The reaction was quenched with 1 M HCl (5 mL), and the organic phase extracted

and washed with sat. aq. $NaHCO_3$ (5 mL) and brine (5 mL). The solvent was evaporated under reduced pressure and **S2** was obtained as a yellow oil (310 mg, 60%).

Benzyl (4-(benzylamino)-3,4-dioxobutyl)(methyl)carbamate (1)

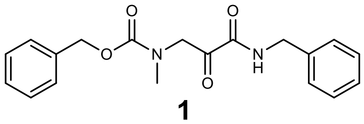

1

To a solution of *N*-methylhydroxylamine hydrochloride (74 mg, 0.88 mmol, 1.1 eq.), $NaHCO_3$ (134 mg, 1.57 mmol, 2 eq.), and 4 Å molecular sieves (600 mg) in 0.8 mL anhydrous MeOH was added **S2** (160 mg, 0.8 mmol, 1 eq.) and the mixture was stirred for 30 minutes at room temperature. To this solution was added benzyl isocyanide (100 mg, 0.84 mmol, 1.05 eq.) and AcOH (400 µL, 7.20 mmol, 9 eq.), and the mixture was stirred for 24 hours at room temperature. The reaction mixture was filtered, and the solvent evaporated under reduced pressure. The crude product was purified by silica chromatography (12 g, 60% EtOAc in n-hexane, R_f=0.6) to obtain **1** as a yellow oil (50 mg, 20%). **HR-MS** (ESI^+): calculated for $C_{19}H_{21}N_2O_4^+$ $[M+H^+]^+$: m/z = 341.1496, found: m/z = 341.1480. **^{1}H-NMR** (400 MHz, $CDCl_3$): δ = 7.40 – 7.27 (m, 10H), 5.12 (2 s, 2H), 4.73 – 4.68 (2 s, 2H), 4.47 (dd, J = 11.2, 6.1 Hz, 2H), 2.97 (d, J = 1.1 Hz, 3H) ppm (2 Rotamers). **^{13}C-NMR** (126 MHz, $CDCl_3$) δ = 193.63, 193.52, 175.27, 175.26, 159.78, 159.56, 156.79, 156.19, 137.04, 136.97, 136.52, 136.49, 136.41, 128.77, 128.53, 128.08, 127.89, 127.80, 77.64, 77.58, 77.38, 77.13, 75.30, 67.57, 67.43, 55.41, 54.94, 43.18, 43.17, 35.92, 35.40, 20.71.

Benzyl (5-(benzylcarbamoyl)-5-hydroxy-3,6-di(pyridin-2-yl)-4,5-dihydropyridazin-4-yl)(methyl)carbamate (3), Benzyl (5-(benzylcarbamoyl)-3,6-di(pyridin-2-yl)pyridazin-4-yl)(methyl)carbamate (4), and 3-benzyl-1-methyl-5,8-di(pyridin-2-yl)pyrimido[4,5-d]pyridazine-2,4(1H,3H)-dione (5)

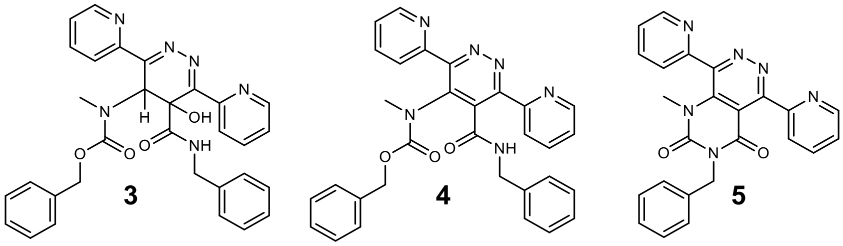

3 **4** **5**

To a solution of phosphate-buffered saline (PBS) (40 mL) were added **1** (from a 1 M stock solution in DMSO, 100 µL, 100 µmol, 1 eq.) and **2** (from a 25 mM stock solution in DMSO, 4 mL, 100 µmol, 1 eq.) and the mixture was shaken at 180 rpm at 37 °C for 18 hours. The solution was added to a solid phase extraction (SPE) column (Strata® C18-E, 55 µm, 70 Å, 10 g/60 mL Giga Tubes, Phenomenex, prewashed with five column volumes MeOH + 0.1% formic acid and five column volumes H_2O + 0.1% formic acid). The column was washed with five column volumes H_2O + 0.1% formic acid, then five column volumes 30% acetonitrile/H_2O + 0.1% formic acid. The sample was eluted in ten column volumes acetonitrile + 0.1% formic acid and the solvent evaporated under reduced pressure. The crude product was purified by reverse-phase High Performance Liquid Chromatography (HPLC) on a Phenomenex Kinetex® 5 µm C18 column (250 x 10 mm) with solvent A: H_2O + 0.1% formic acid, solvent B: acetonitrile + 0.1% formic acid by the following gradient method: B 5% ramped up to 70% over the first 2 minutes, then slow gradient to 95% at 17 minutes, then to 100% B over 1 minute, 100% B for 2 minutes, then again 5% B for 3 more minutes to equilibrate. Fractions containing **3** (1.0 mg, 1.8%), **4** (1.2 mg, 2.2%) or **5** (1.6 mg, 3.8%) were separately combined and dried by lyophilization.

3: **HR-MS** (ESI^+): calculated for $C_{31}H_{29}N_6O_4^+$ $[M+H^+]^+$: *m/z* = 549.2214, found: *m/z* = 549.2214. **^{1}H NMR** (400 MHz, $CDCl_3$) δ 8.56 (ddd, *J* = 5.0, 1.8, 0.9 Hz, 1H), 7.99 (d, *J* = 8.1 Hz, 1H), 7.78 (td, *J* = 7.9, 1.8 Hz, 1H), 7.38 (d, *J* = 4.6 Hz, 1H), 7.34 – 7.30 (m, 4H), 7.23 (s, 2H), 7.21 – 7.05 (m, 5H), 6.72 (d, *J* = 7.4 Hz, 2H), 4.95 (d, *J* = 12.3 Hz, 1H), 4.80 (s, 1H), 4.45 (s, 1H), 4.02 (s, 1H), 2.99 (s, 3H).
4: **HR-MS** (ESI^+): calculated for $C_{31}H_{27}N_6O_3^+$ $[M+H^+]^+$: *m/z* = 531.2139, found: *m/z* = 531.2112.
5: **HR-MS** (ESI^+): calculated for $C_{24}H_{19}N_6O_2^+$ $[M+H^+]^+$: *m/z* = 423.1564, found: *m/z* = 423.1539. **^{1}H NMR** (400 MHz, $CDCl_3$) δ 8.73 (dddd, *J* = 5.8, 5.0, 1.7, 0.9 Hz, 2H), 8.19 (dt, *J* = 7.9, 1.1 Hz, 1H), 7.99 (dtd, *J* = 9.3, 7.7, 1.8 Hz, 2H), 7.79 (dq, *J* = 7.8, 0.9 Hz, 1H), 7.52 – 7.43 (m, 4H), 7.30 (s, 4H), 5.19 (s, 2H), 3.09 (s, 3H). **^{13}C NMR** (101 MHz, $CDCl_3$) δ 158.78, 157.39, 155.86, 155.48, 151.23, 149.78, 149.04, 148.77, 139.76, 137.72, 137.03, 135.65, 129.36, 128.49, 127.98, 124.81, 124.31, 123.99, 123.69, 111.87, 77.33, 77.22, 77.02, 76.70, 45.48, 37.59, 1.02.

2,2',2''-(10-(2-((4-(1,2,4,5-tetrazin-3-yl)benzyl)amino)-2-oxoethyl)-1,4,7,10-tetraazacyclododecane-1,4,7-triyl)triacetate (DOTA-Tetrazine, 7)

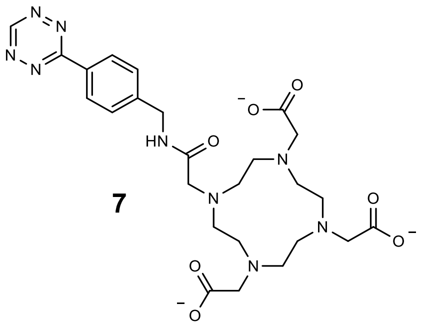

7

1,4,7,10-Tetraazacyclododecane-1,4,7,10-tetraacetic acid mono-*N*-hydroxysuccinimide ester (17.1 mg, 22.5 µmol, 1 eq.) and (4-(1,2,4,5-tetrazin-3-yl)phenyl)methanamine hydrochloride (5 mg, 22.5 µmol, 1 eq.) were dissolved in 0.75 mL dimethylformamide (DMF) in a pear-shaped flaks and added with DIPEA (14.54 mg, 112.5 µmol, 5 eq.) and stirred 16 hours at room temperature. The solvent was evaporated under reduced pressure and the reaction mixture dissolved in 50% acetonitrile/H_2O. DOTA-Tetrazine **7** was purified by reverse-phase HPLC on a Phenomenex Luna 5µm C18 column (250 x 10 mm) with solvent A: H_2O + 0.1% formic acid, solvent B: acetonitrile + 0.1% formic acid by the following gradient method: B 5% ramped up to 15% over the first 2 minutes, then a slow gradient to 95% at 17 minutes, then to 100% B over 1 minute, 100% B for 2 minutes, then again 5% B for 3 more minutes to equilibrate. Fractions containing the product were dried to obtain a pink solid (4.9 mg, 40%). **HR-MS** (ESI^+): calculated for $C_{25}H_{36}N_9O_7^+$ $[M+H^+]^+$: *m/z* = 574.2732, found: *m/z* = 574.2746. **^{1}H-NMR** (400 MHz, DMSO-d_6): δ = 10.62 (s, 1H), 9.07 (s, 1H) 8.50 (d, *J* = 8 Hz, 2H), 7.61 (d, *J* = 8 Hz, 2H), 4.50 (d, *J* = 5.6 Hz, 2H), 4.04 (d, *J* = 15.8 Hz, 4H), 3.64 (b, 4H), 3.43 - 3.06 (m, 16H) ppm.

Supplementary figures and tables

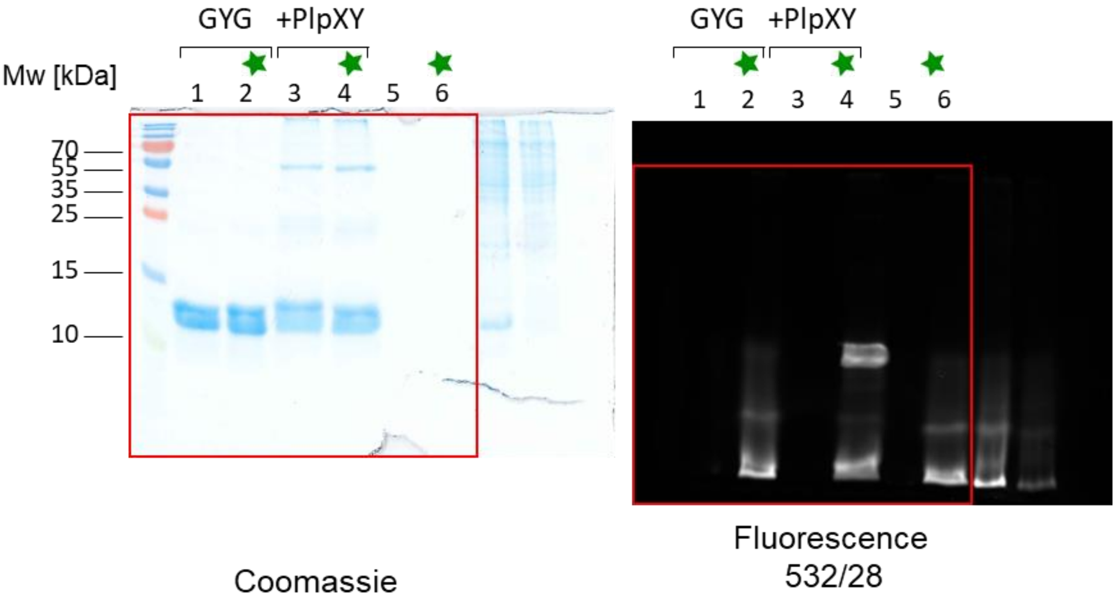

Supplementary Figure 1. Full gel images for PlpA3-M5G without (GYG, lanes 1-2) and with (+PlpXY, lanes 3-4) co-expression of PlpXY incubated overnight with CF®488A Tetrazine **6** (10 µM, star symbol). The left gel shows Coomassie-stained protein bands, the right gel shows gel fluorescence using 488 nm excitation and 532/28 nm emission filter. Lane 6 is a control without protein, lanes to the right are unrelated. Cropped images used in the main text are illustrated with red rectangles.

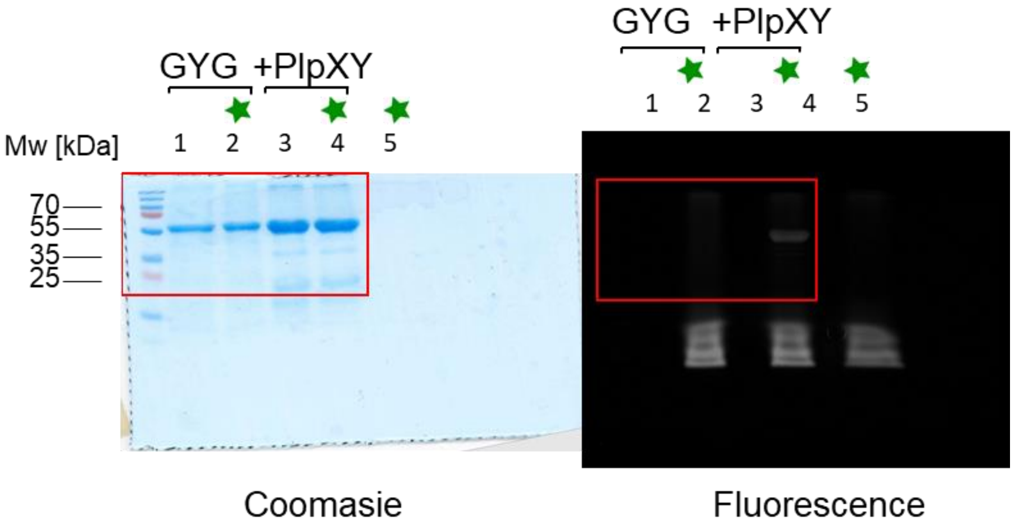

Supplementary Figure 2. Full gel images for FtsZ without (GYG, lanes 1-2) and with (+PlpXY, lanes 3-4) co-expression of PlpXY incubated overnight with CF®488A Tetrazine **6** (1 µM, star symbol). The left gel shows Coomassie-stained protein bands, the right gel shows gel fluorescence using 488 nm excitation and

532/28 nm emission filter. Lane 5 is a control without protein, lanes to the right are unrelated. Cropped images used in the main text are illustrated with red rectangles.

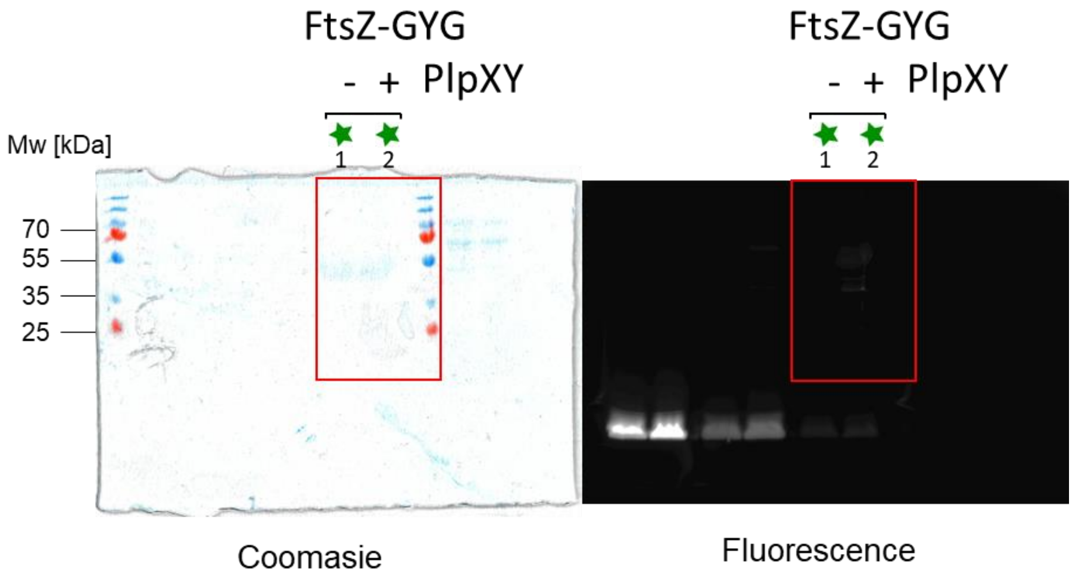

Supplementary Figure 3. Full gel images for FtsZ without (-, lane 1) and with (+, lane 2) co-expression of PlpXY in *E. coli* stained with CF®488A Tetrazine **6** (1 µM, star symbol) for 1 hour (with addition of 0.3% TritonX-100). Cells were lyzed and proteins purified by Ni-NTA affinity purification to visualize successful intracellular labelling. The left gel shows Coomassie-stained protein bands, the right gel shows gel fluorescence using 488 nm excitation and 532/28 nm emission filter. Other lanes to the right are unrelated. Cropped images used in Figure S4 are illustrated with red rectangles.

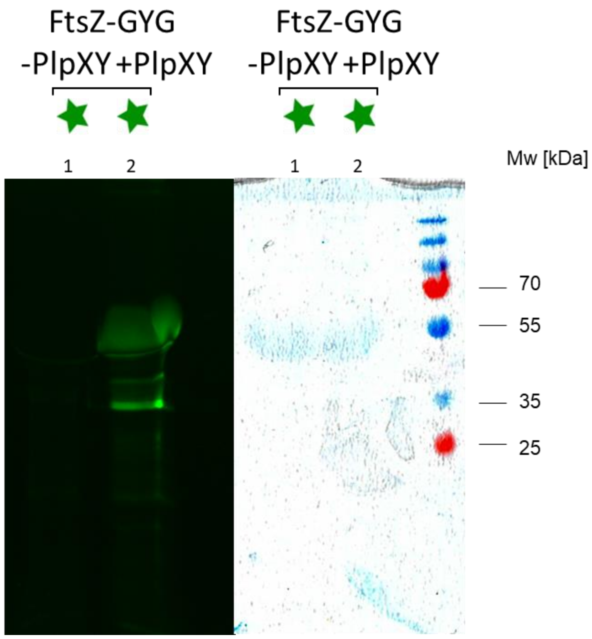

Supplementary Figure 4. Gel images for FtsZ without (-, lane 1) and with (+, lane 2) co-expression of PlpXY in *E. coli* stained with CF®488A Tetrazine **6** (100 µM, star symbol) for 1 hour (with addition of 0.3% TritonX-100). Cells were washed with PBS, lysed and proteins purified by Ni-NTA affinity purification to visualize successful intracellular labelling. The left gel shows Coomassie-stained protein bands, the right gel shows gel fluorescence using 488 nm excitation and 532/28 nm emission filter. Only the co-expression shows fluorescence for purified FtsZ. The experiment was performed once.

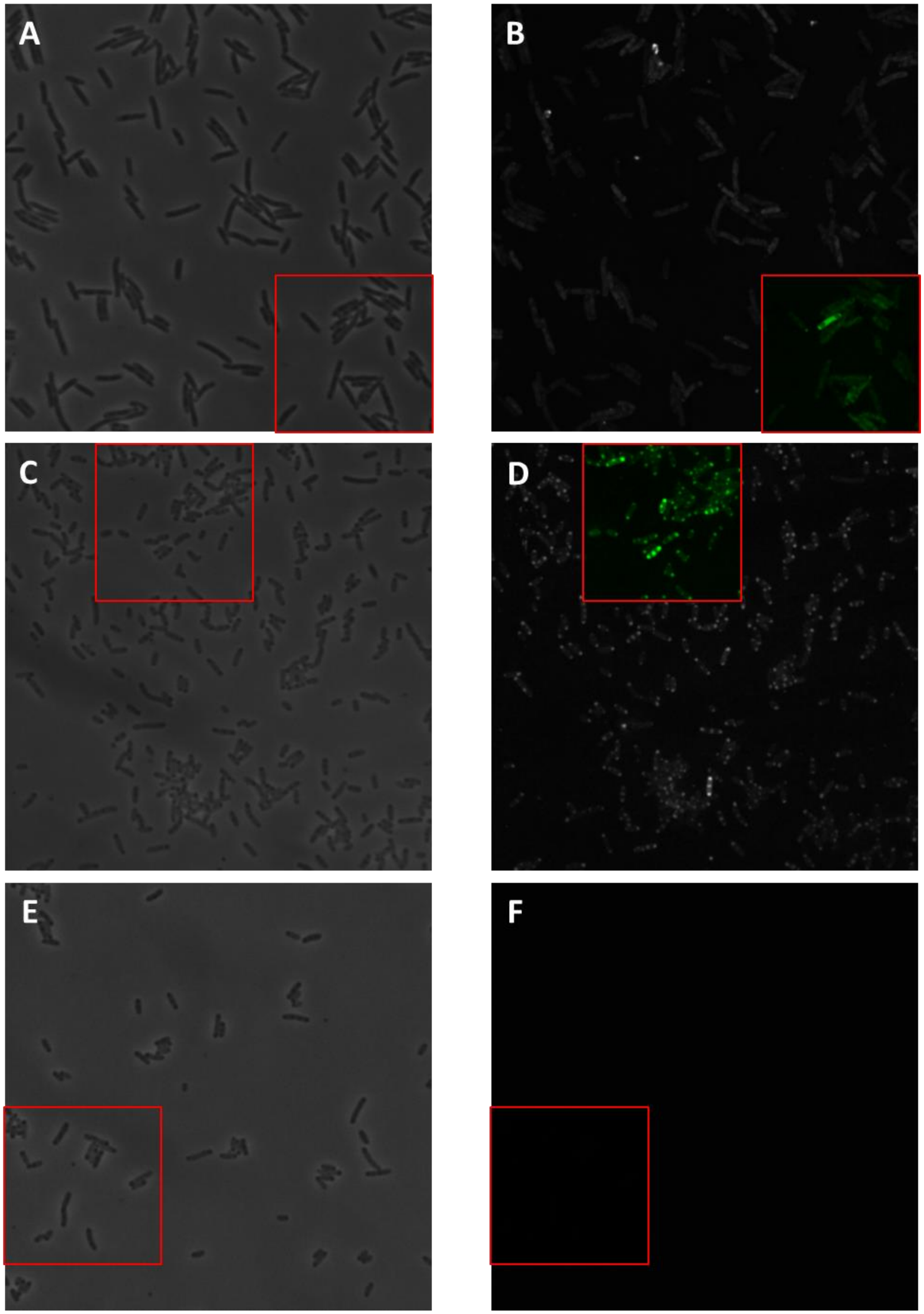

Supplementary Figure 5. Full confocal microscopy images for FtsZ-G55 without (A,B) and with co-expression of PlpXY (C, D, E, F) in brightfield (A ,C, E) and GFP channel (B, D, F). Cells were stained with CF488A® tetrazine **6** (A,B,C,D). Cropped images used in the main text are illustrated with the red rectangle.

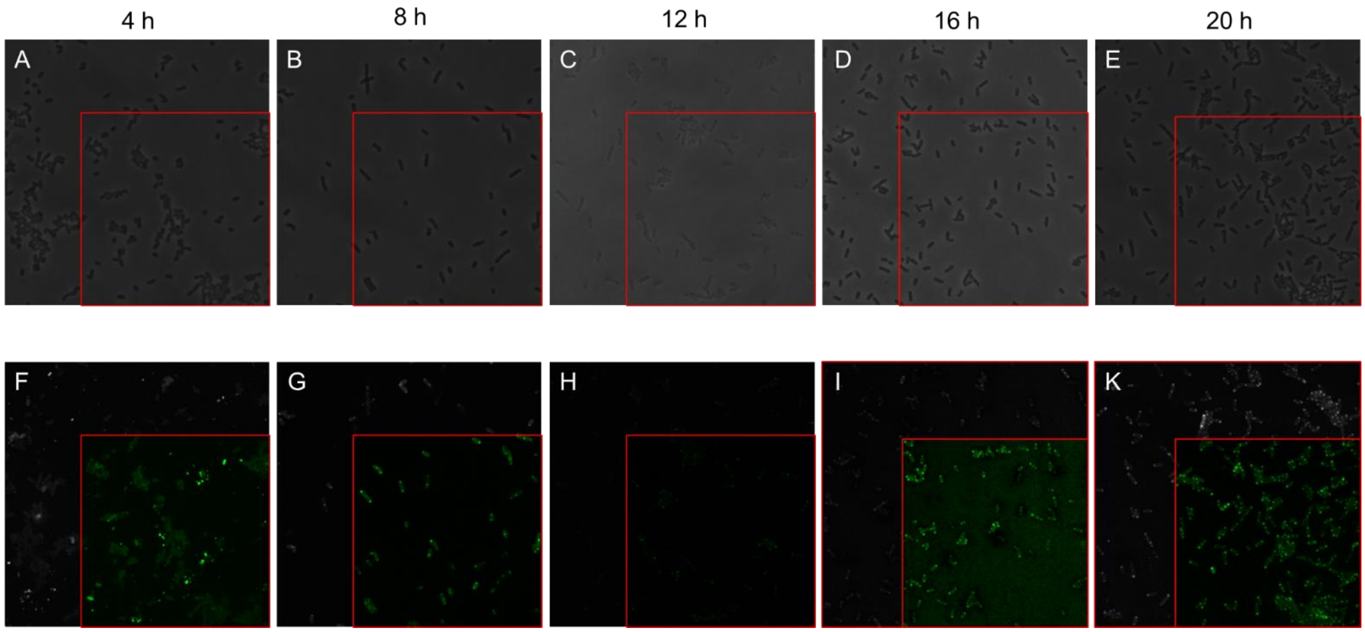

Supplementary Figure 6. Full confocal microscopy images for FtsZ-G55 with co-expression of PlpXY in brightfield (A, B, C, D, E) and GFP channel (F, G, H, I, K). Cells were stained with CF488A® tetrazine **6**. Cropped images used in the main text are illustrated with the red rectangle.

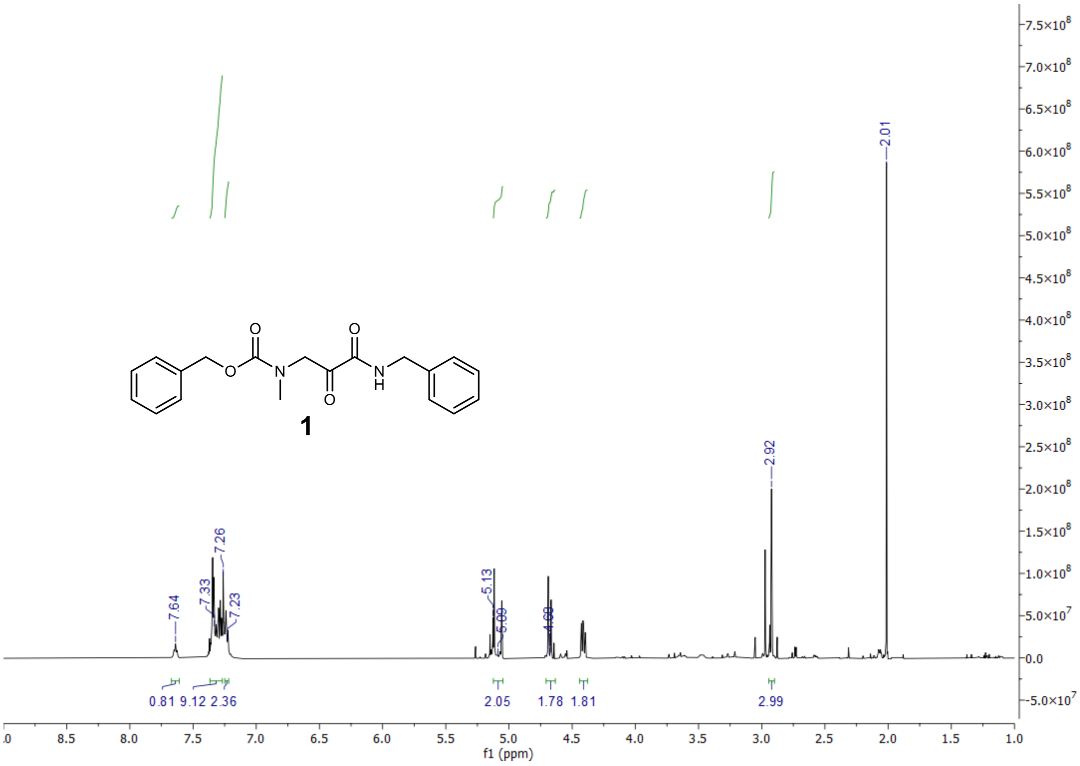

Supplementary Figure 7. ^{1}H-NMR (400 MHz) spectrum of aminopyruvate model compound **1** in $CDCl_3$.

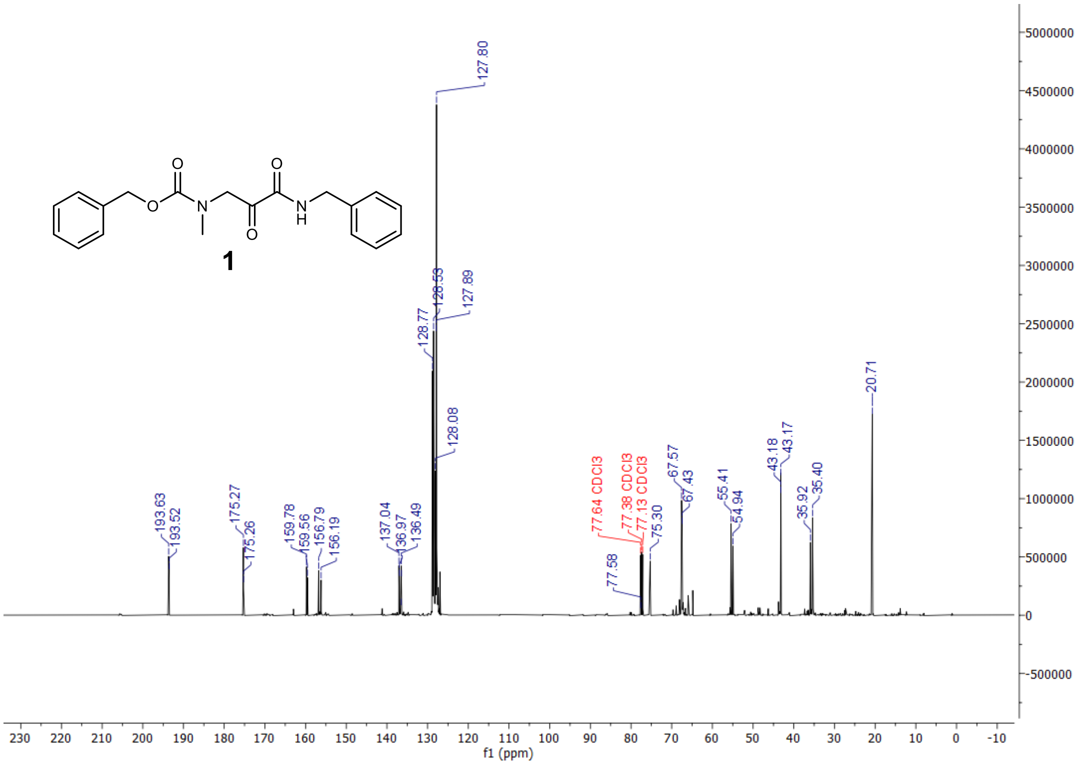

Supplementary Figure 8. ^{13}C-NMR (126 MHz) spectrum of aminopyruvate model compund **1** in $CDCl_3$.

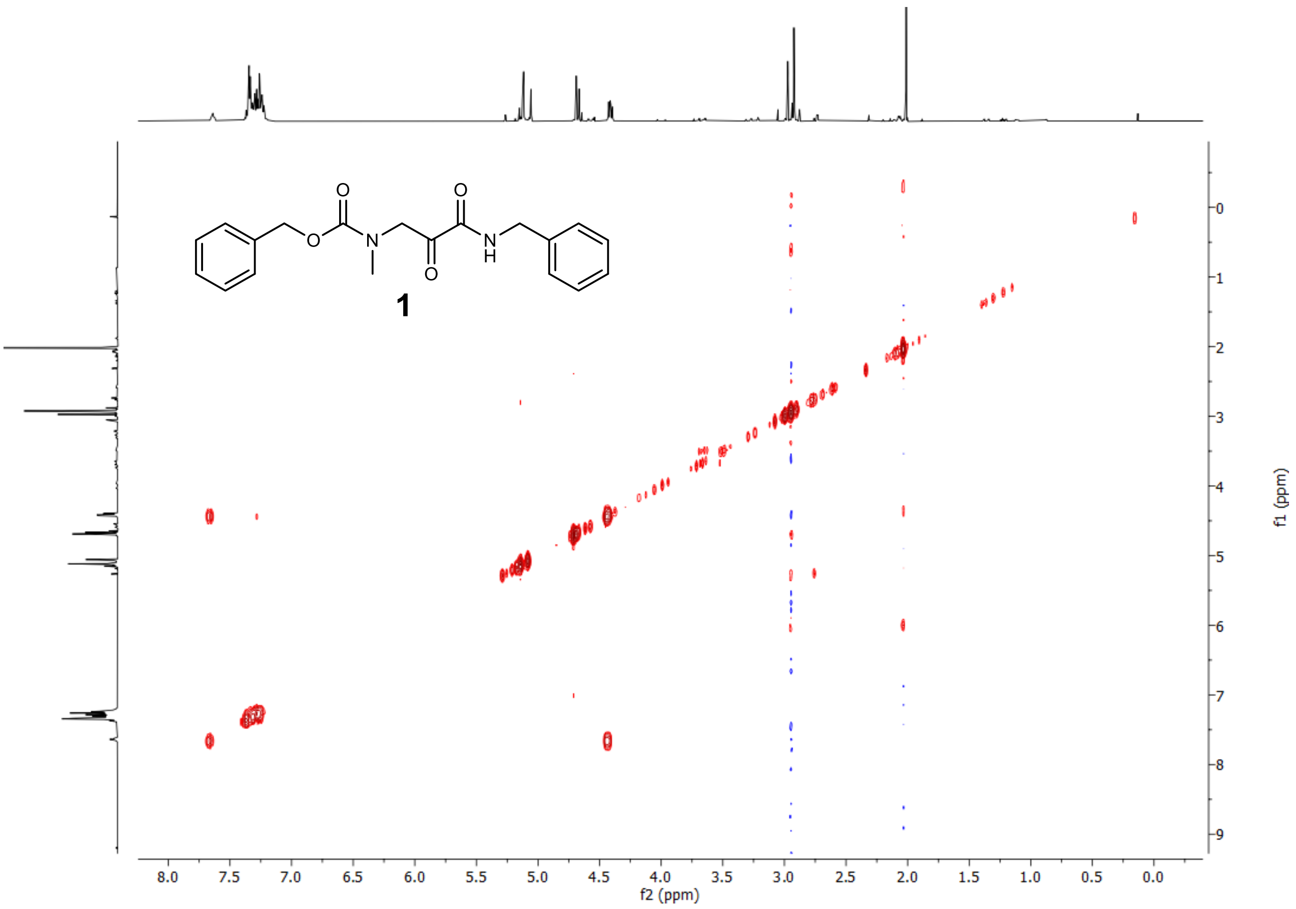

Supplementary Figure 9. ^{1}H-^{1}H-COSY NMR (500 MHz) spectrum of aminopyruvate model compound **1** in $CDCl_3$.

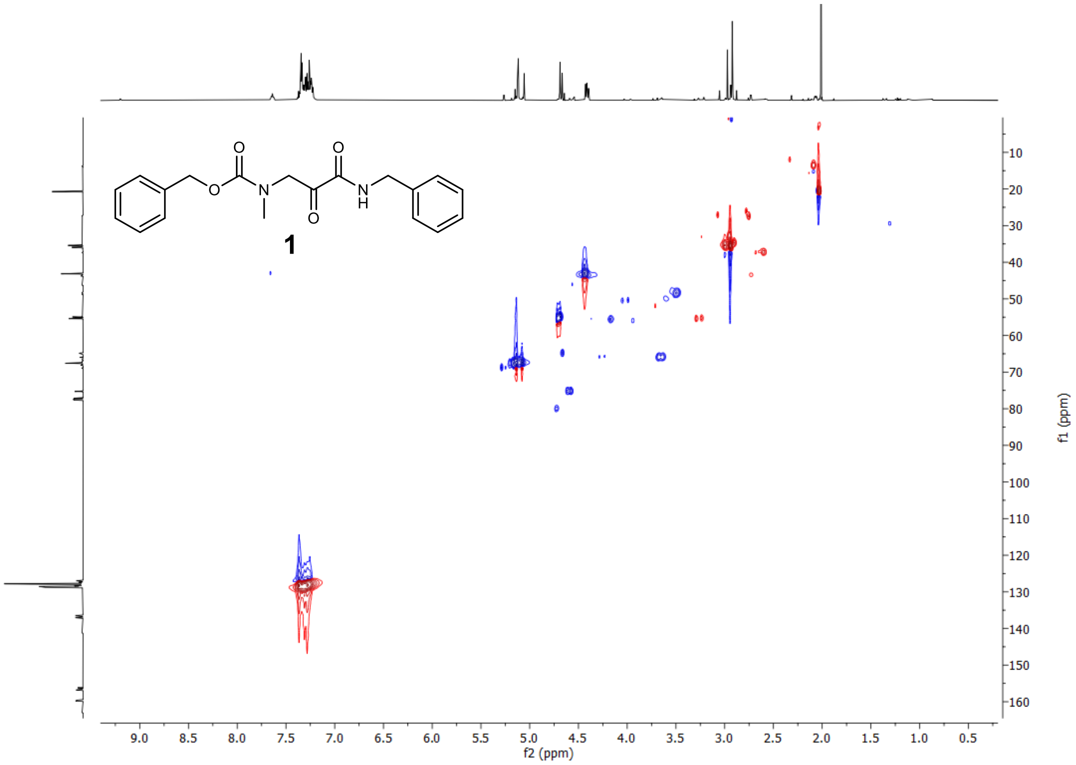

Supplementary Figure 10. HSQC (500 MHz) spectrum of aminopyruvate model compound **1** in $CDCl_3$.

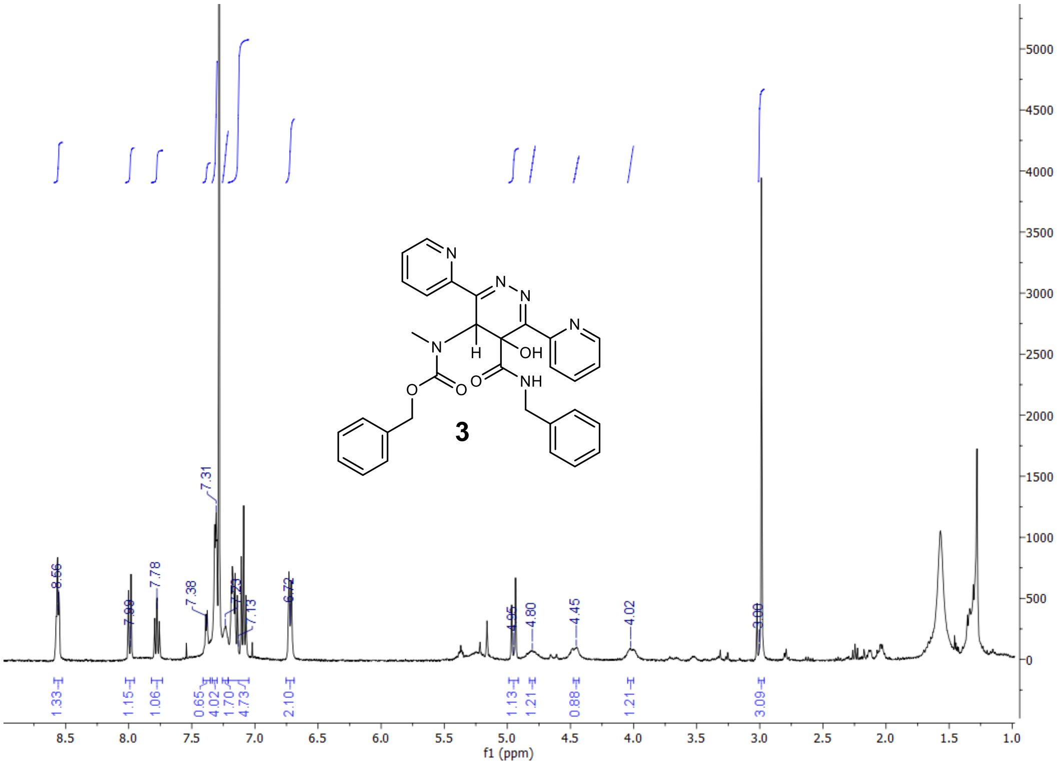

Supplementary Figure 11. ^{1}H-NMR (400 MHz) spectrum of **3** in $CDCl_3$.

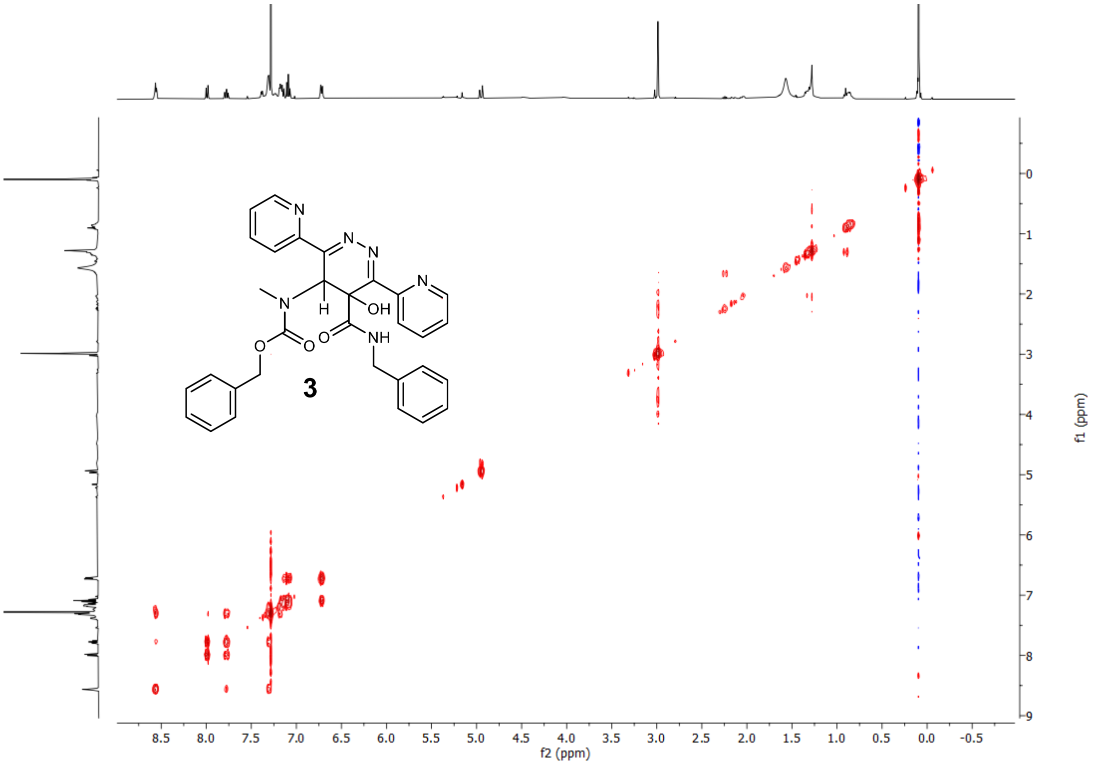

Supplementary Figure 12. ^{1}H-^{1}H-COSY (400 MHz) spectrum of **3** in $CDCl_3$.

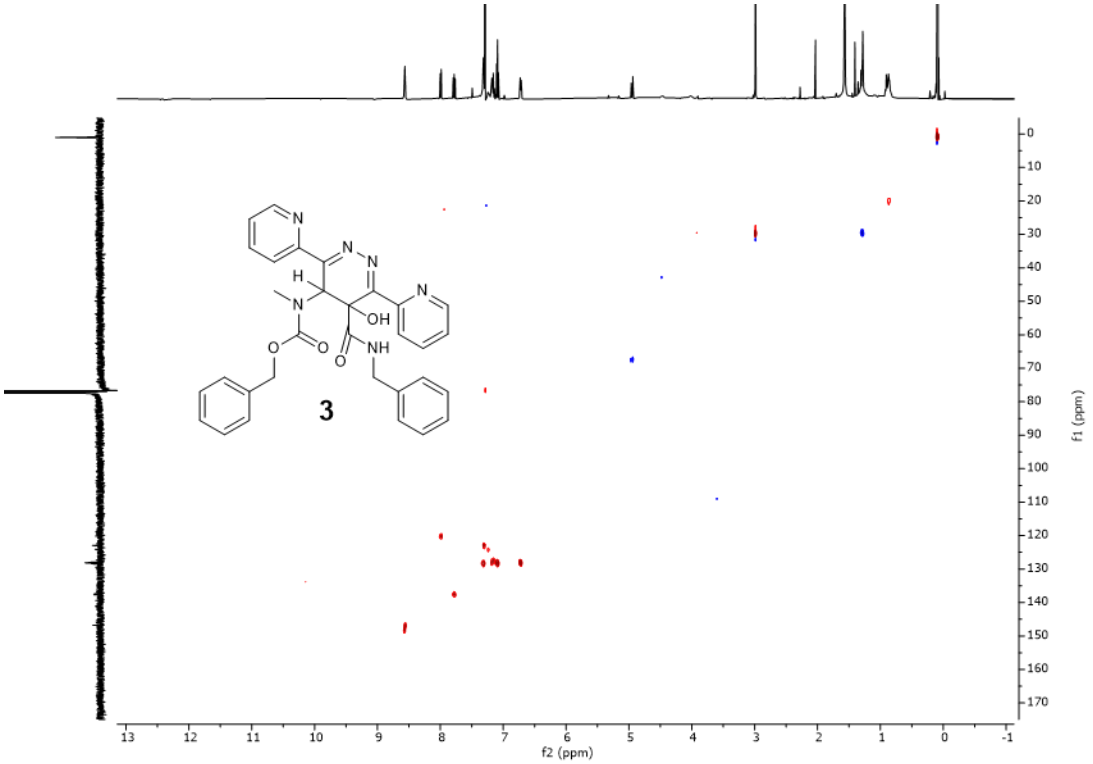

Supplementary Figure 13. HSQC (400 MHz) spectrum of **3** in $CDCl_3$.

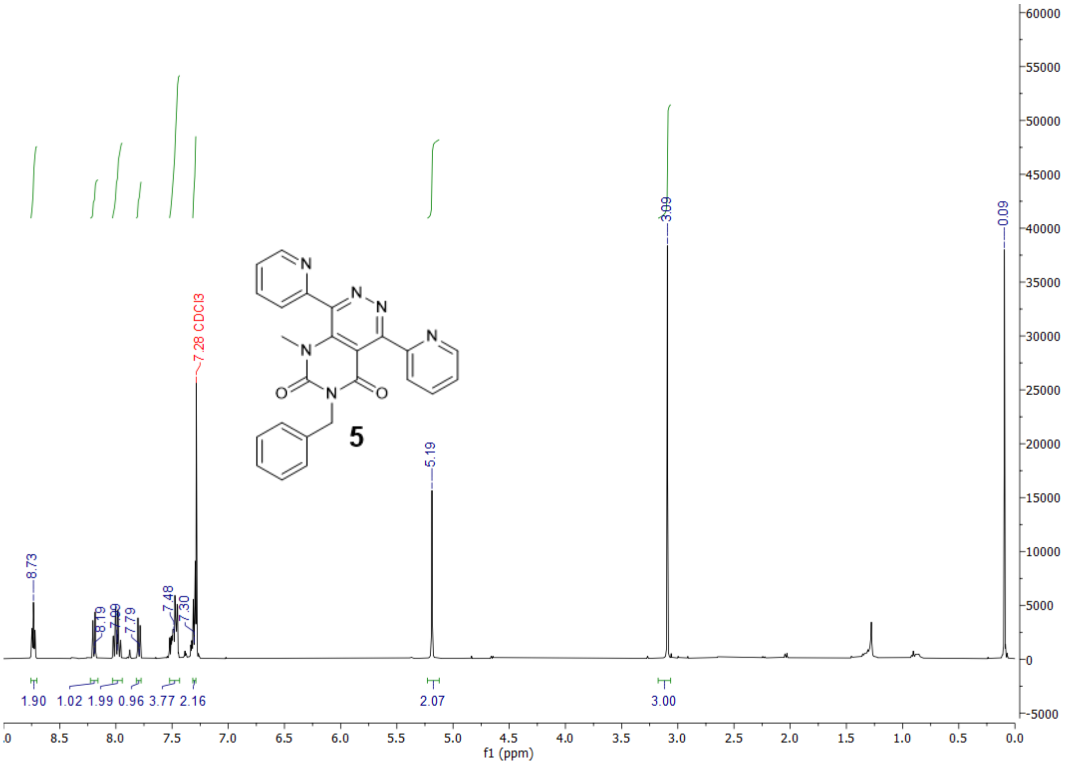

Supplementary Figure 14. ^{1}H-NMR (400 MHz) spectrum of **5** in $CDCl_3$.

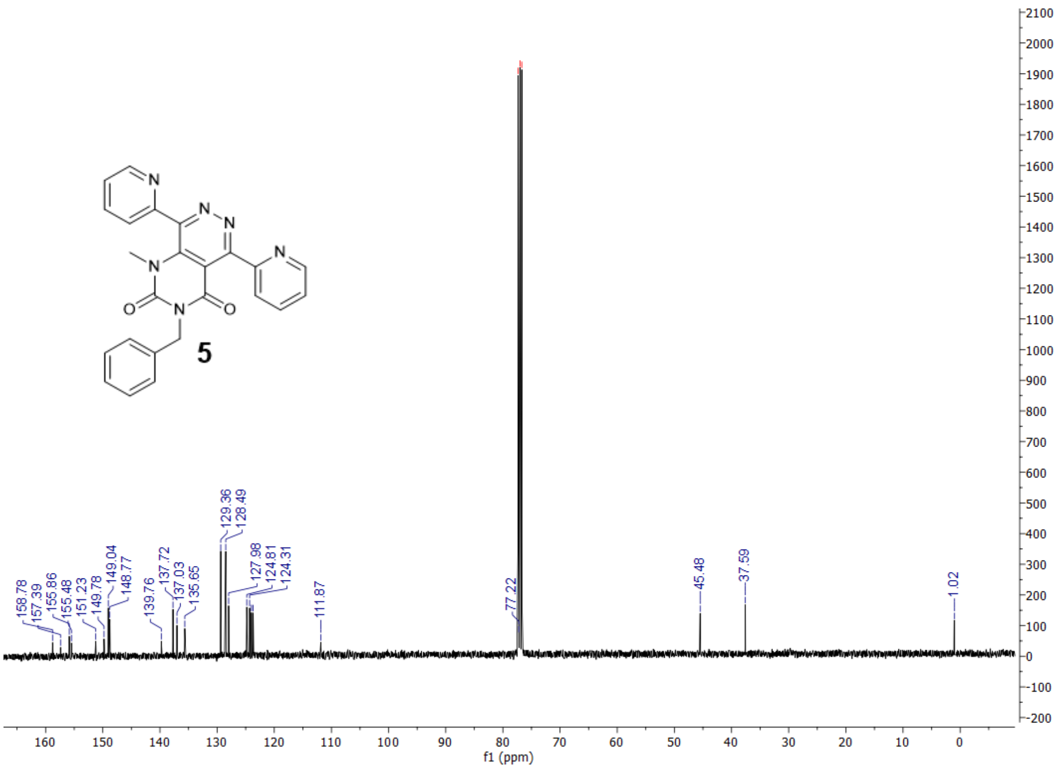

Supplementary Figure 15. ^{13}C-NMR (101 MHz) spectrum of **5** in $CDCl_3$.

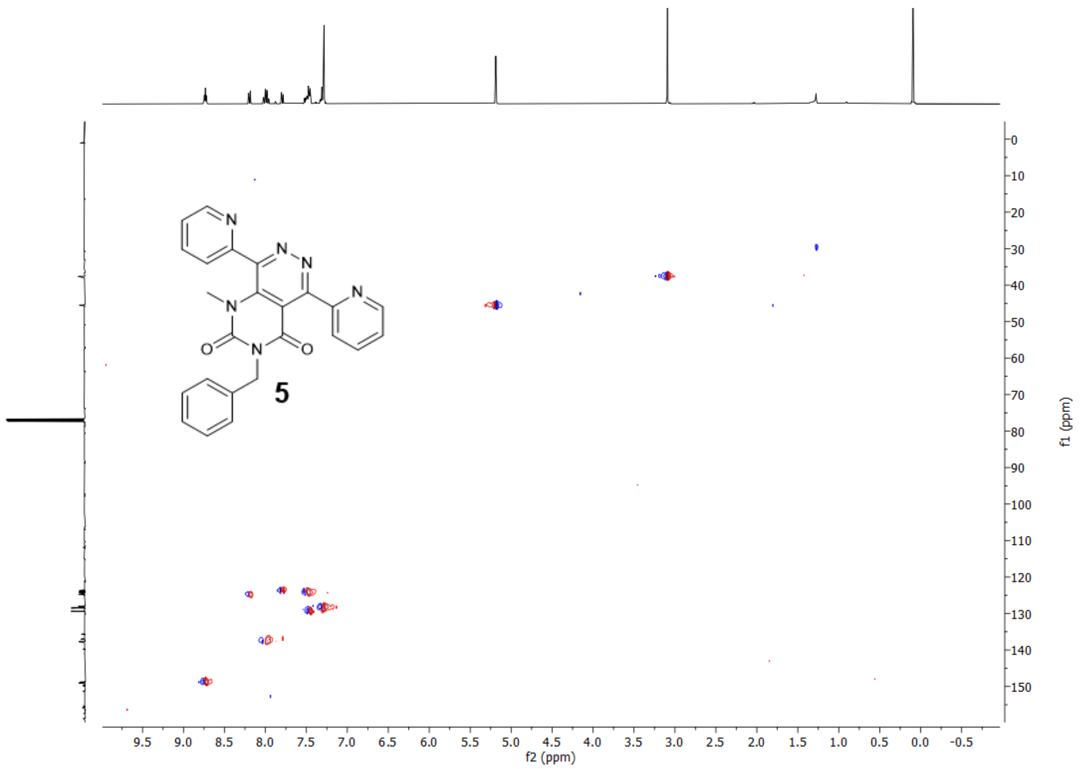

Supplementary Figure 16. HSQC (400 MHz) spectrum of **5** in $CDCl_3$.

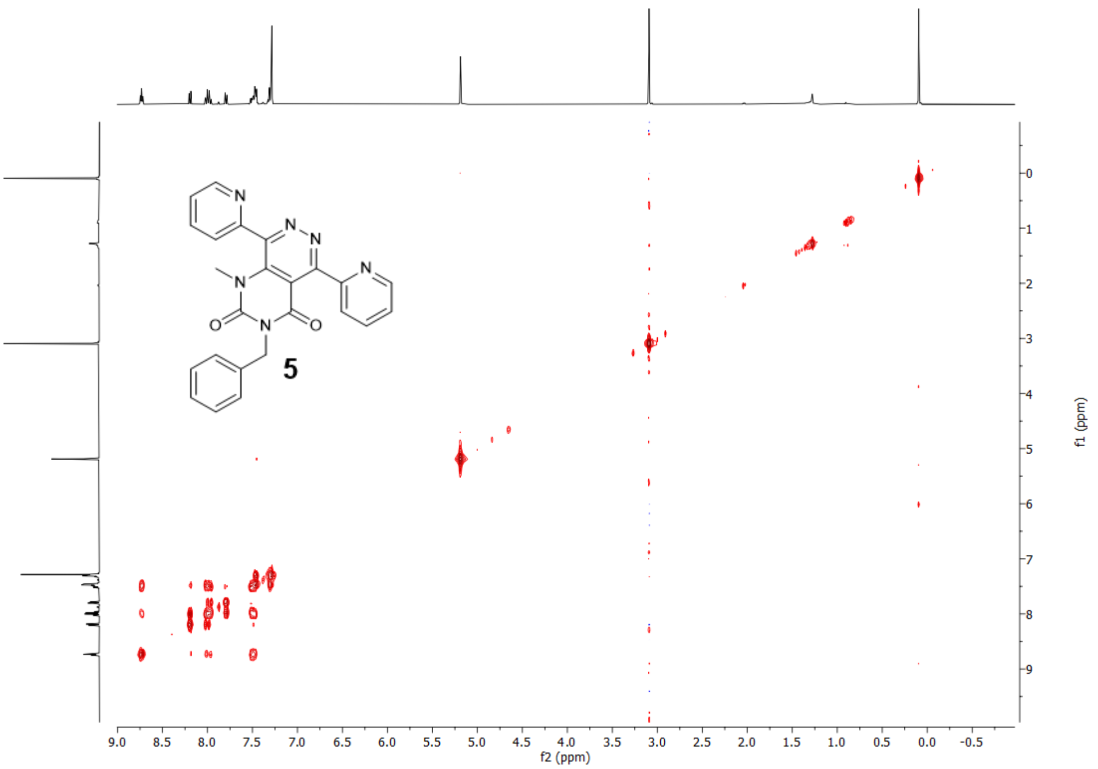

Supplementary Figure 17. ^{1}H-^{1}H-COSY-NMR (400 MHz) spectrum of **5** in $CDCl_3$.

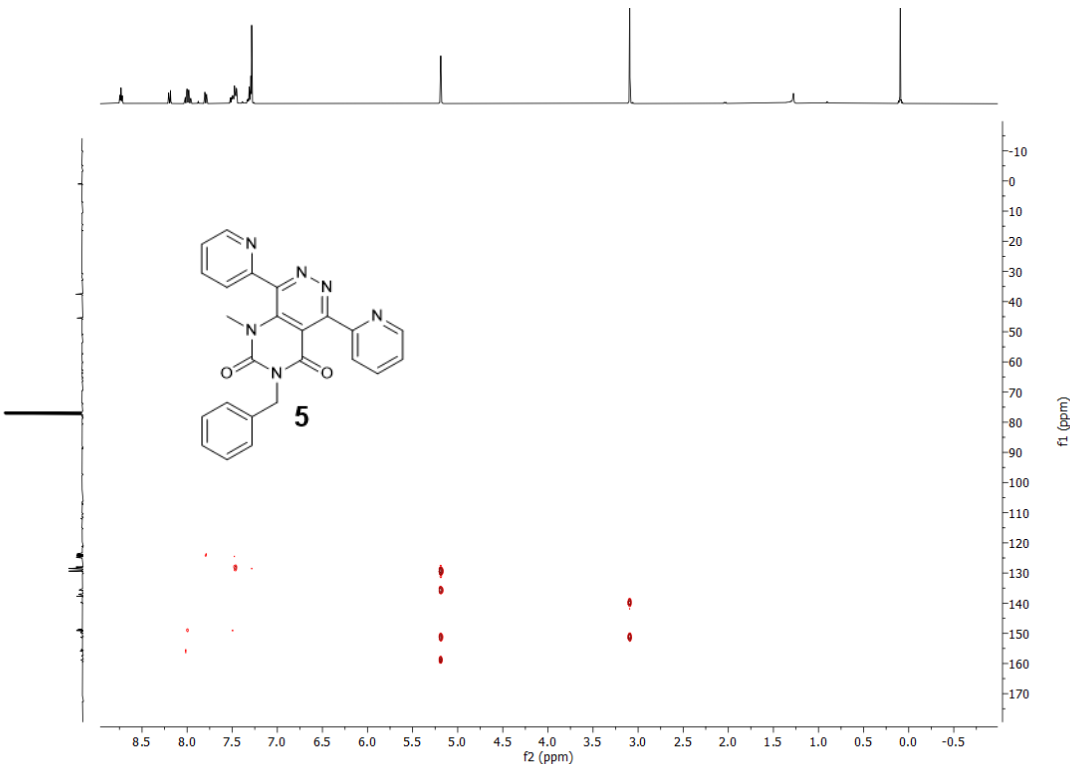

Supplementary Figure 18. HMBC (400 MHz) spectrum of **5** in $CDCl_3$.

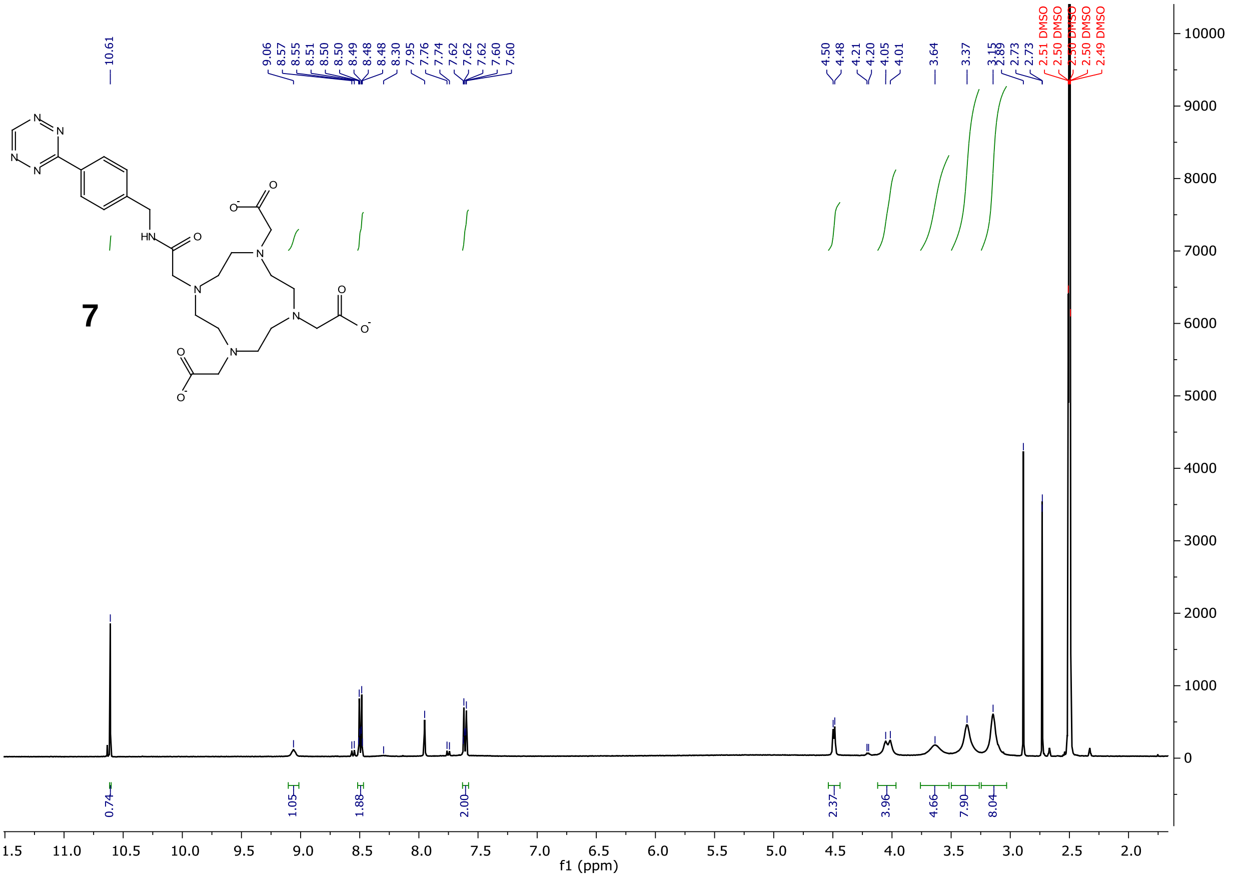

Supplementary Figure 19. ^{1}H-NMR (400 MHz) spectrum of DOTA-Tetrazine **7** in DMSO-d_6.

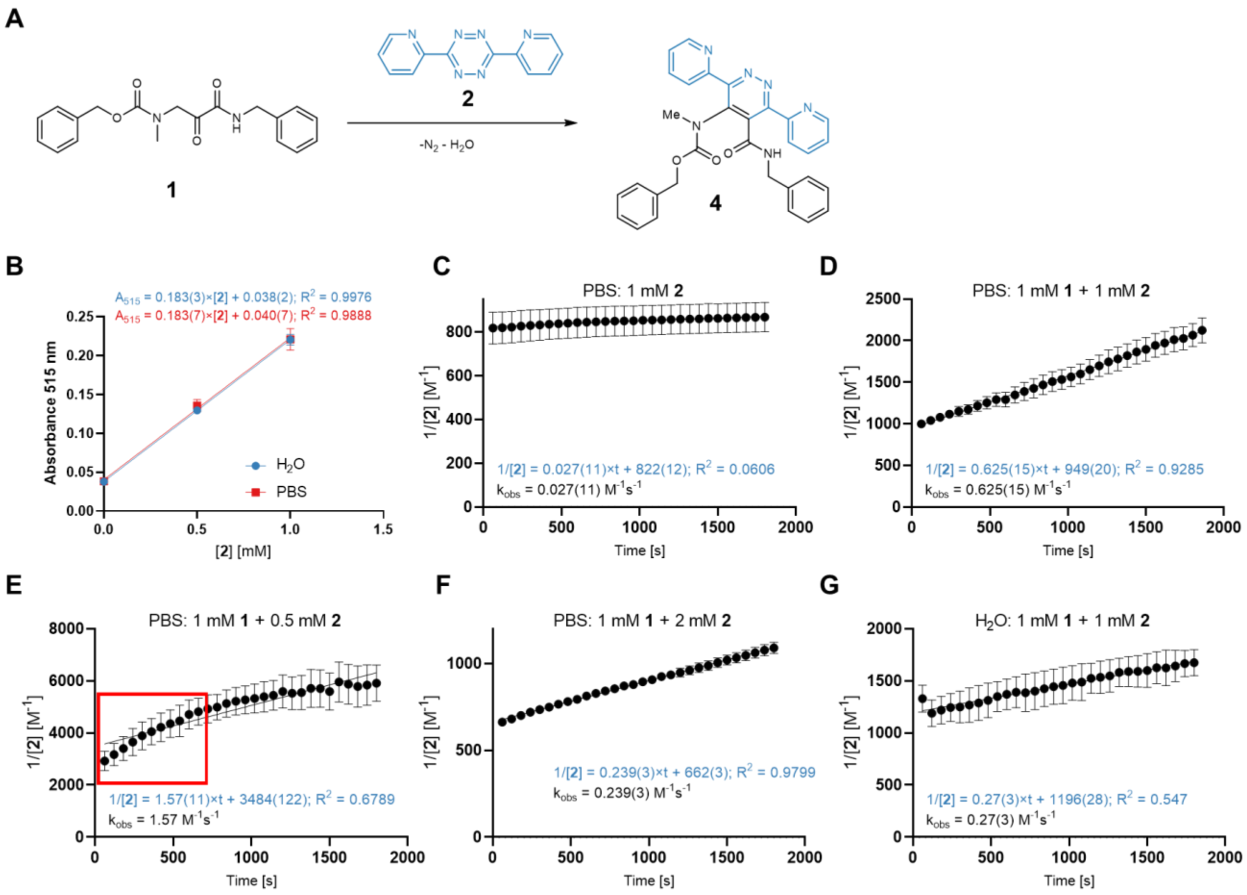

Supplementary Figure 20. Kinetic measurements for the reaction between model compound **1** and 3,6,-di-2-pyridyl-1,2,4,5-tetrazine **2** by spectrometry (515 nm) in PBS or water at 25 °C. A) Reaction of **1** and **2** leads to the formation of **4** through loss of nitrogen and water. B) Calibration curve of absorbance and concentration of tetrazine measured in PBS or H_2O in triplicates (H_2O: slope: 0.183(3); y-intercept: 0.038(2) R^2:0.9976; PBS: slope: 0.183(7); y-intercept: 0.040(7) R^2:0.9888). C) Compound **2** in PBS over time, only minor degradation was detected (k_{obs} = 0.027(11) $M^{-1}s^{-1}$). D) Absorbance over time for 1 mM **1** and 1 mM **2** in PBS, yielding k_{obs} = 0.625(15) $M^{-1}s^{-1}$. E) Absorbance over time for 1 mM **1** and 0.5 mM **2** in PBS, yielding k_{obs} = 1.57(11) $M^{-1}s^{-1}$. A systematic deviation from linearity can be observed during the first 5 minutes. F) Absorbance over time for 1 mM **1** and 2 mM **2** in PBS, yielding k_{obs} = 0.239(3) $M^{-1}s^{-1}$. G) Absorbance over time for 1 mM **1** and 1 mM **2** in H_2O, yielding k_{obs} = 0.27(3) $M^{-1}s^{-1}$. Points describe mean; error bars describe standard deviation (n=3 independent experiments for all).

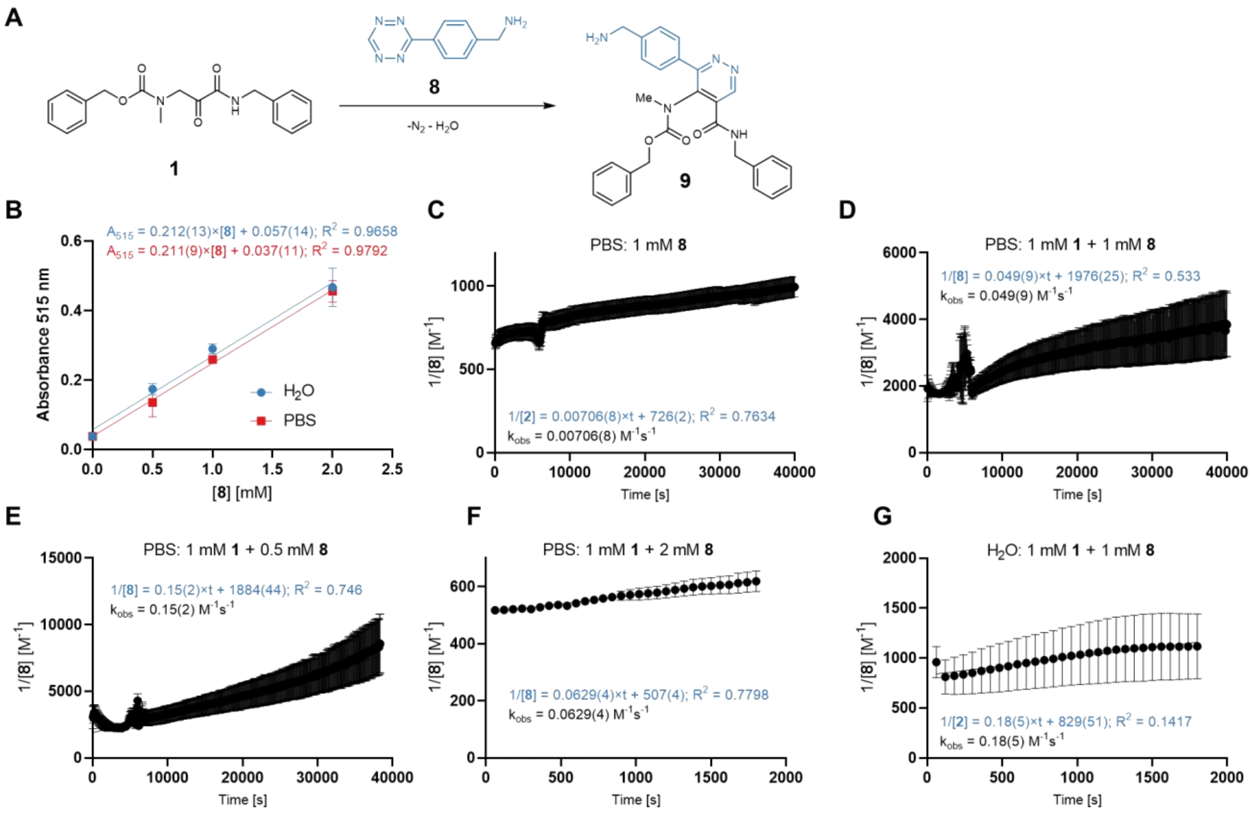

Supplementary Figure 21. Kinetic measurements for the reaction between model compound **1** and (4-(1,2,4,5-tetrazin-3-yl)phenyl)methanamine hydrochloride **8** by spectrometry (515 nm) in PBS or water at 25 °C. A) Reaction of **1** and **8** leads to the formation of **9** through loss of nitrogen and water. B) Calibration curve of absorbance and concentration of tetrazine **8** measured in PBS or H_2O in triplicates (H_2O: slope: 0.212(13); y-intercept: 0.057(14) R^2:0.9658; PBS: slope: 0.211(9); y-intercept: 0.037(11) R^2:0.9792). C) Compound **8** in PBS over time, only minor degradation was detected (k_{obs} = 0.00706(8) $M^{-1}s^{-1}$). D) Absorbance over time for 1 mM **1** and 1 mM **2** in PBS, yielding k_{obs} = 0.049(9) $M^{-1}s^{-1}$. E) Absorbance over time for 1 mM **1** and 0.5 mM **2** in PBS, yielding k_{obs} = 0.15(2) $M^{-1}s^{-1}$. Measurements deviated from linearity in the beginning and were therefore continued for 12 hours. F) Absorbance over time for 1 mM **1** and 2 mM **2** in PBS, yielding k_{obs} = 0.0629(4) $M^{-1}s^{-1}$. Measurements deviated from linearity in the beginning and were therefore continued for 12 hours. G) Absorbance over time for 1 mM **1** and 1 mM **2** in H_2O, yielding k_{obs} = 0.18(5) $M^{-1}s^{-1}$. Points describe mean; error bars describe standard deviation (n=3 independent experiments for all).

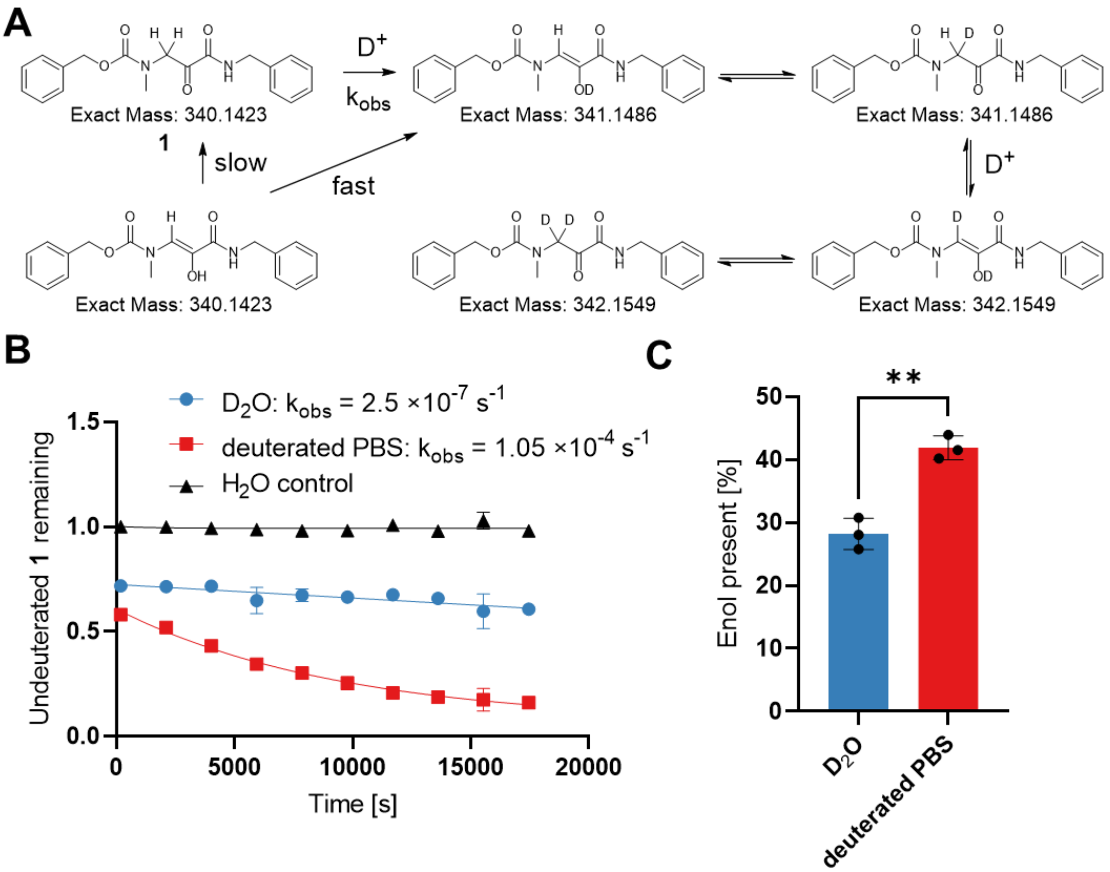

Supplementary Figure 22. Enolization kinetics of **1**. A) Model reaction to measure enolization behaviour of **1**. Initial enol present was measured due to the fast exchange of **1** in deuterated PBS or D_2O in the enol form to the once deuterated enol. Further enolization behaviour of **1** to the deuterated product in either solvent was measured over time by LC/MS. B) Undeuterated **1** remaining over time in H_2O, deuterated PBS and D_2O. Exponential decay fits yield k_{obs}=0.000105 s^{-1} in PBS and k_{obs}=0.0000002511 s^{-1} in D2O (n=3 independent experiments). The line connects mean values, error bars represent standard deviations. C) Initial enol present estimated due to the fast exchange of protonated to deuterated enol in solution. Enol concentration is 28.2 (2.5) % in D_2O and 41.9 (1.9) % in deuterated PBS (n=3 independent experiments, P=0.0016 by a two-tailed t test). Bars describe mean; error bars describe standard deviation.

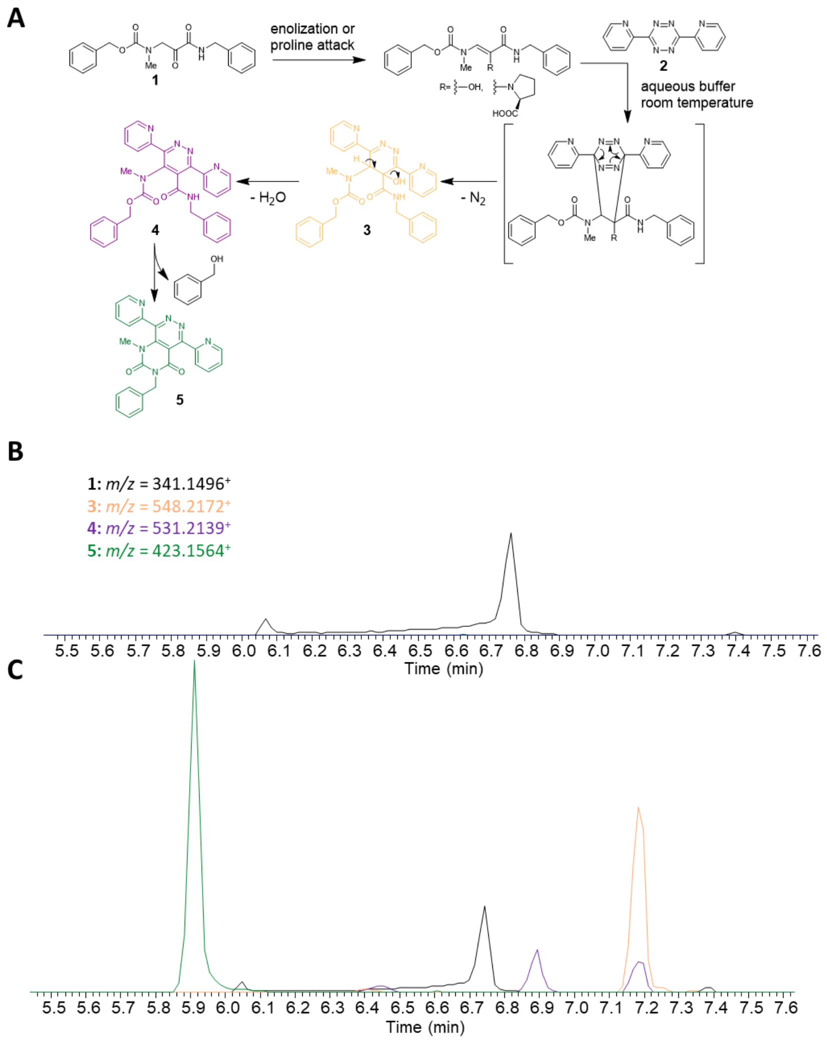

Supplementary Figure 23. A) cycloaddition of **1** and **2**, forming compounds **3**, **4**, and **5.** B) and C) Extracted ion chromatograms for *m/z* 341.1496 (**1**, $[M+H]^+$), *m/z* 548.2172 (**3**, $[M+H]^+$), *m/z* 531.2139 (**4**, $[M+H]^+$), and *m/z* 423.1564 (**5**, $[M+H]^+$), for B) no addition of **2** and C) 16 h incubation of **1** and **2**.

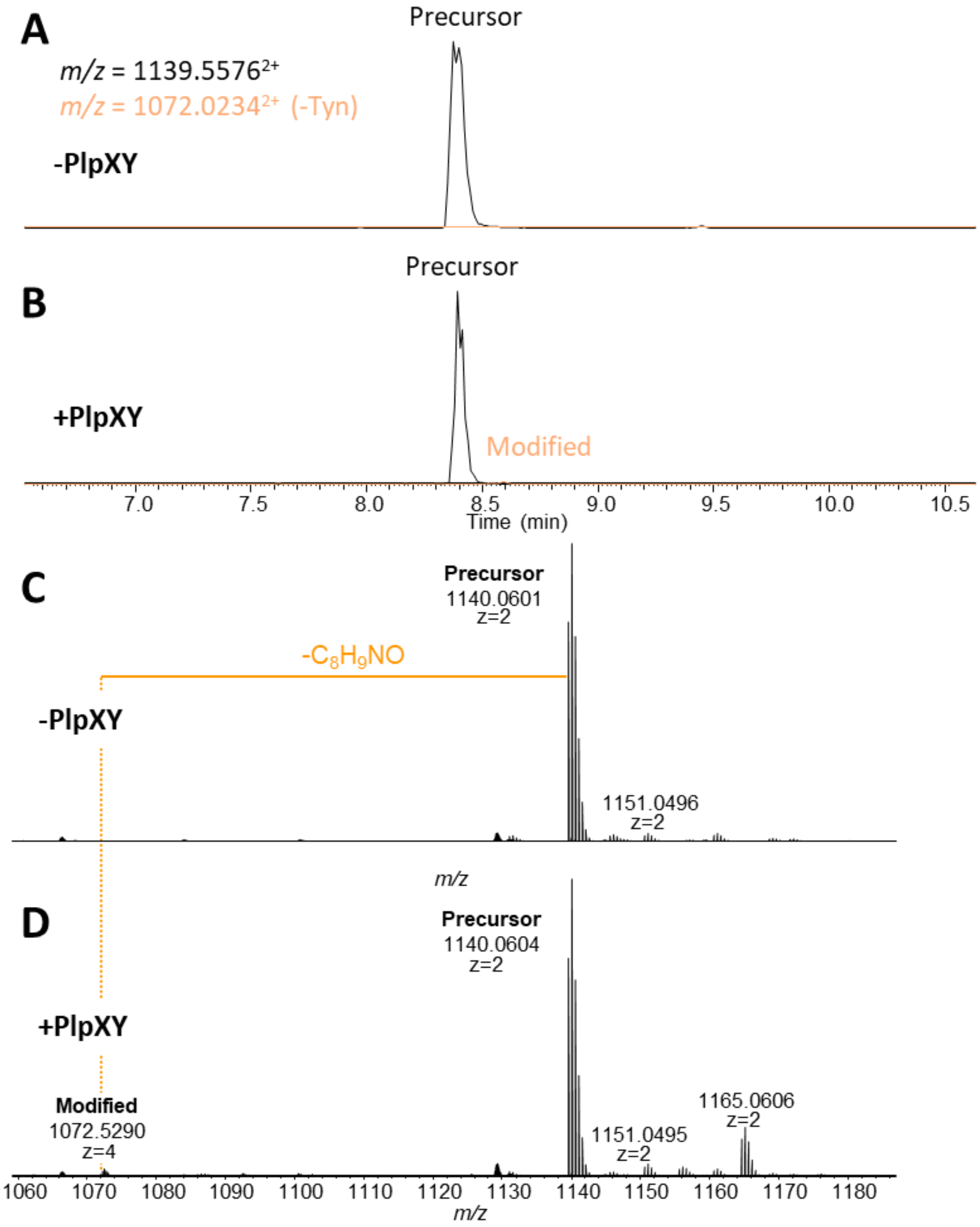

Supplementary Figure 24. LC-MS analysis for His$_6$-PlpA3-M5G cleaved with trypsin to give the peptide fragment AVAAGYGVVFPWDNEFPWPR. Extracted ion chromatograms for *m*/*z* 1139.5576 (unmodified, $[M+2H]^{2+}$), *m*/*z* 1072.0234 (modified, $[M+2H]^{2+}$) for A) precursor only expression and B) precursor + PlpXY co-expression. Extracted mass spectra for C) precursor only expression and D) precursor + PlpXY coexpression. "Modified" refers to excision of tyramine (-C_8H_9NO).

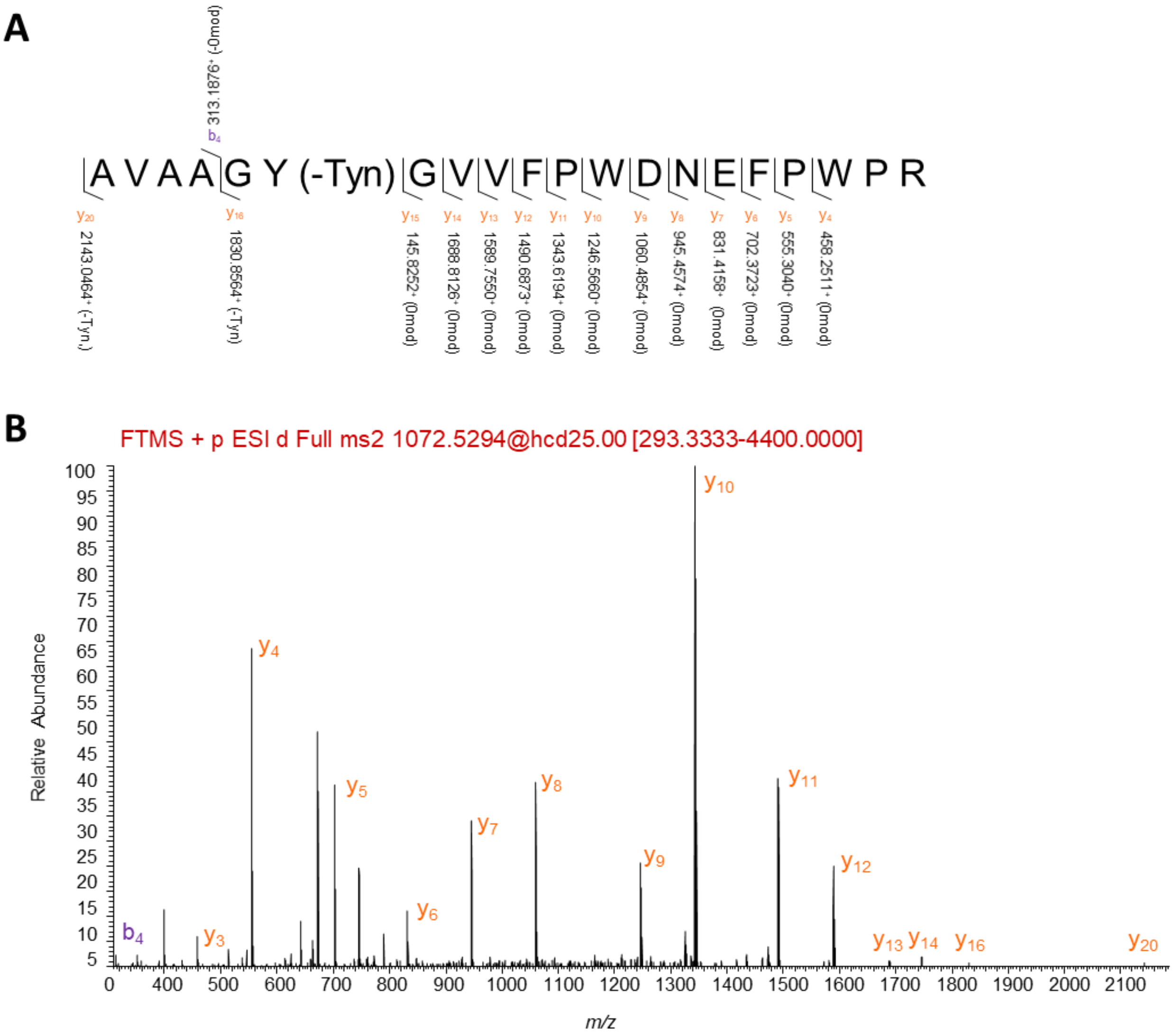

Supplementary Figure 25. Localization of C_8H_8NO loss from His$_6$-PlpA3-M5G cleaved with trypsin to give peptide fragment AVAAGYGVVFPWDNEFPWPR. A) Summary of observed b (above, purple) and y (below, orange) fragmentation ions. B) MS/MS spectrum from parallel reaction monitoring (PRM)-mediated fragmentation (CE 25) from *m/z* 1072.5294 ($[M+2H]^{2+}$) parent ion. Observed b and y ions are indicated as before.

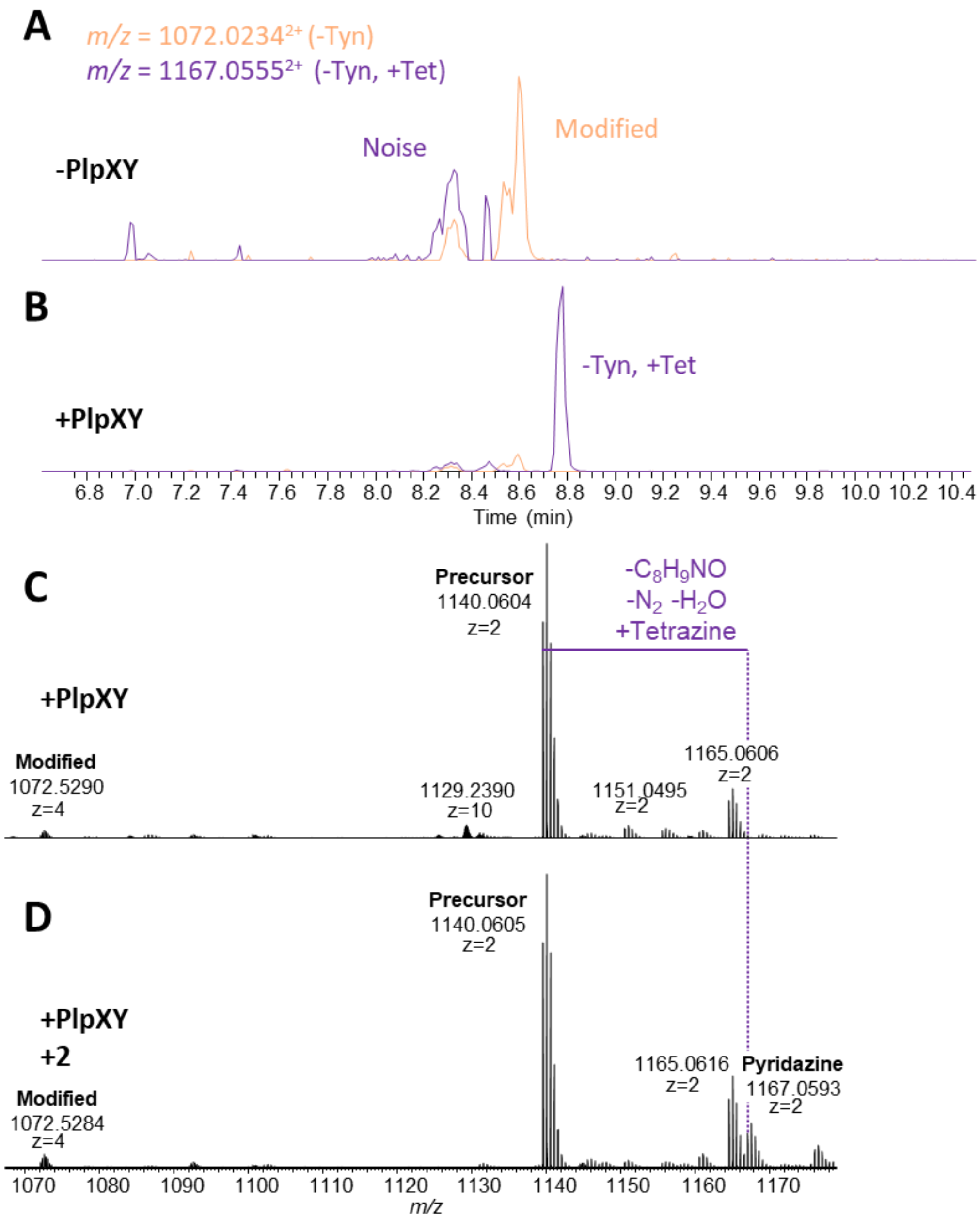

Supplementary Figure 26. LC-MS analysis for conjugation of tetrazine **2** with His_6-PlpA3-M5G cleaved with trypsin to give the peptide fragment AVAAGYGVVFPWDNEFPWPR. Extracted ion chromatograms for *m/z* 1139.5576 (unmodified, $[M+2H]^{2+}$), *m/z* 1072.0234 (modified, $[M+2H]^{2+}$), and *m/z* 1167.0555 (pyridazine, $[M+2H]^{2+}$) for A) precursor + PlpXY co-expression and B) overnight incubation with tetrazine **2**. Extracted mass spectra for C) precursor + PlpXY co-expression and D) overnight incubation with tetrazine **2**. "Modified" refers to excision of tyramine ($-C_8H_9NO$) and "pyridazine" to cycloaddition product formation.

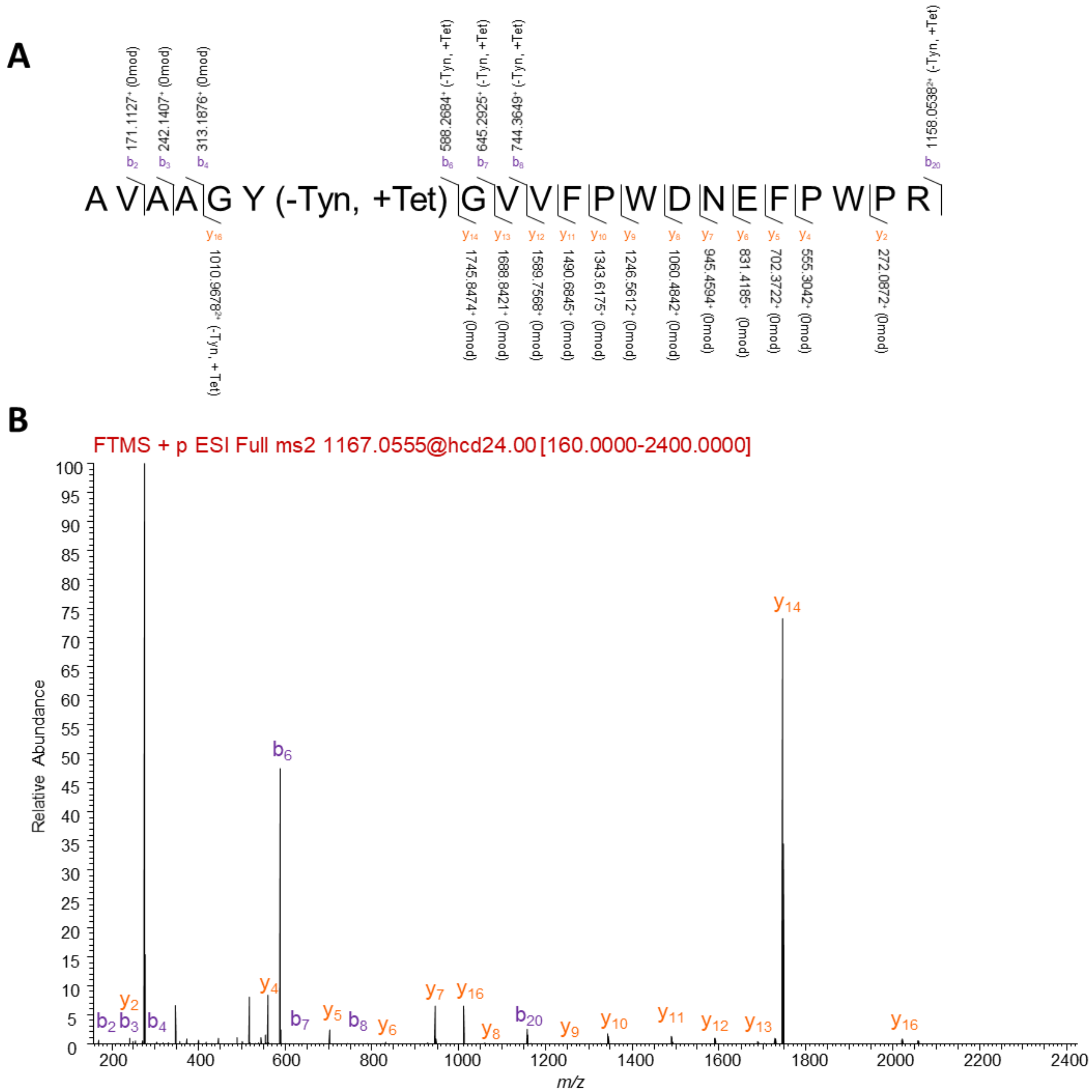

Supplementary Figure 27. Localization of pyridazine formation on His$_6$-PlpA3-M5G (with tetrazine **2**) cleaved with trypsin to give peptide fragment AVAAGYGVVFPWDNEFPWPR. A) Summary of observed b (above, purple) and y (below, orange) fragmentation ions. B) MS/MS spectrum from PRM-mediated fragmentation (CE 24) from *m/z* 1167.0555 ($[M+2H]^{2+}$) parent ion. Observed b and y ions are indicated as before.

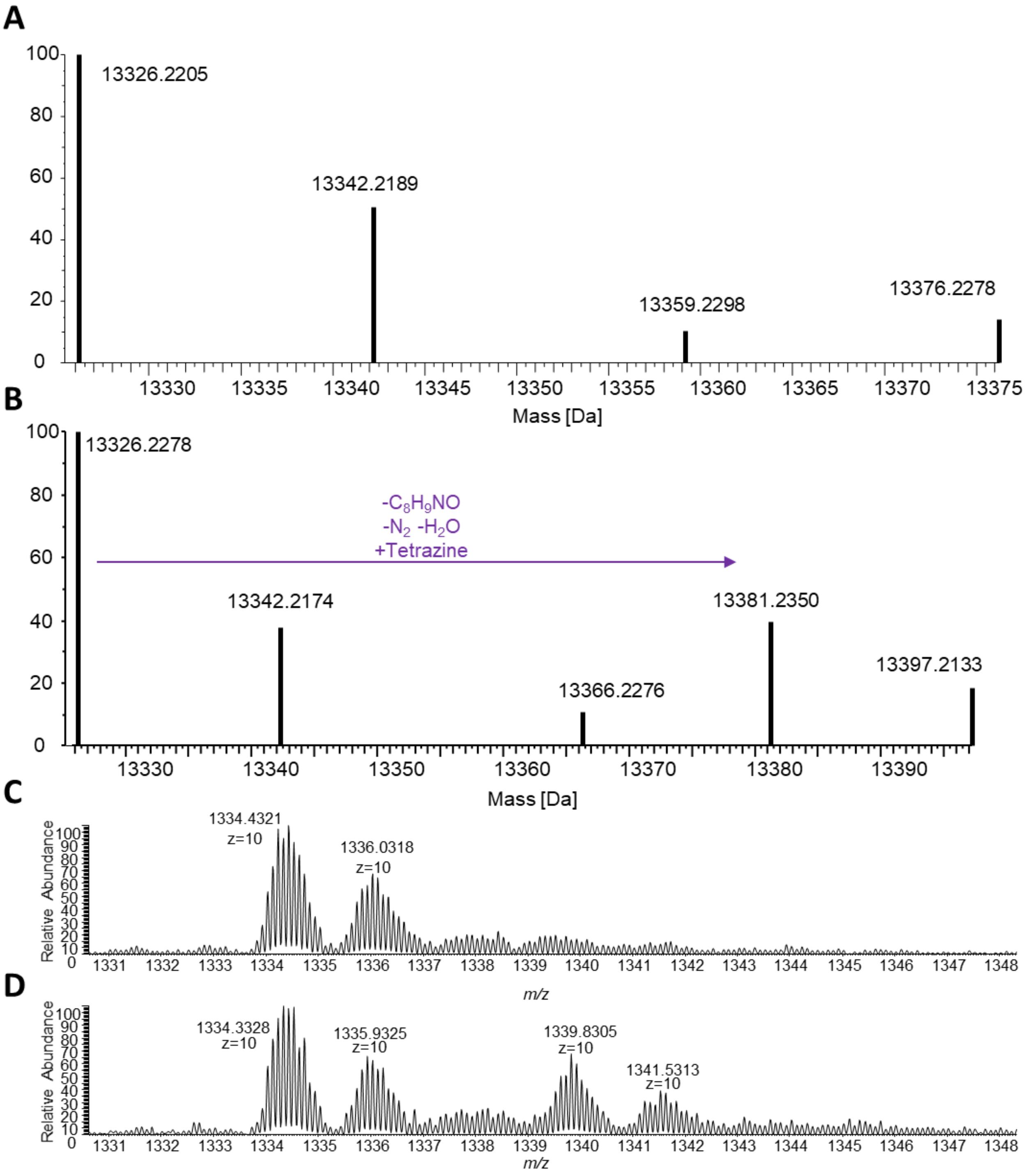

Supplementary Figure 28. LC-MS analysis of tetrazine **2** with His$_6$-PlpA3-M5G. Deconvoluted mass spectra for A) unconjugated (obs. 13326.2278 Da, calcd. 13326.2110 Da) and B) conjugated (obs. 13381.2350 Da, calcd. 13381.2237 Da) sample, deconvoluted with ThermoFisher Freestyle 1.3. Raw MS for retention time 10.01-10.38 for C) unconjugated and D) conjugated protein used for deconvolution.

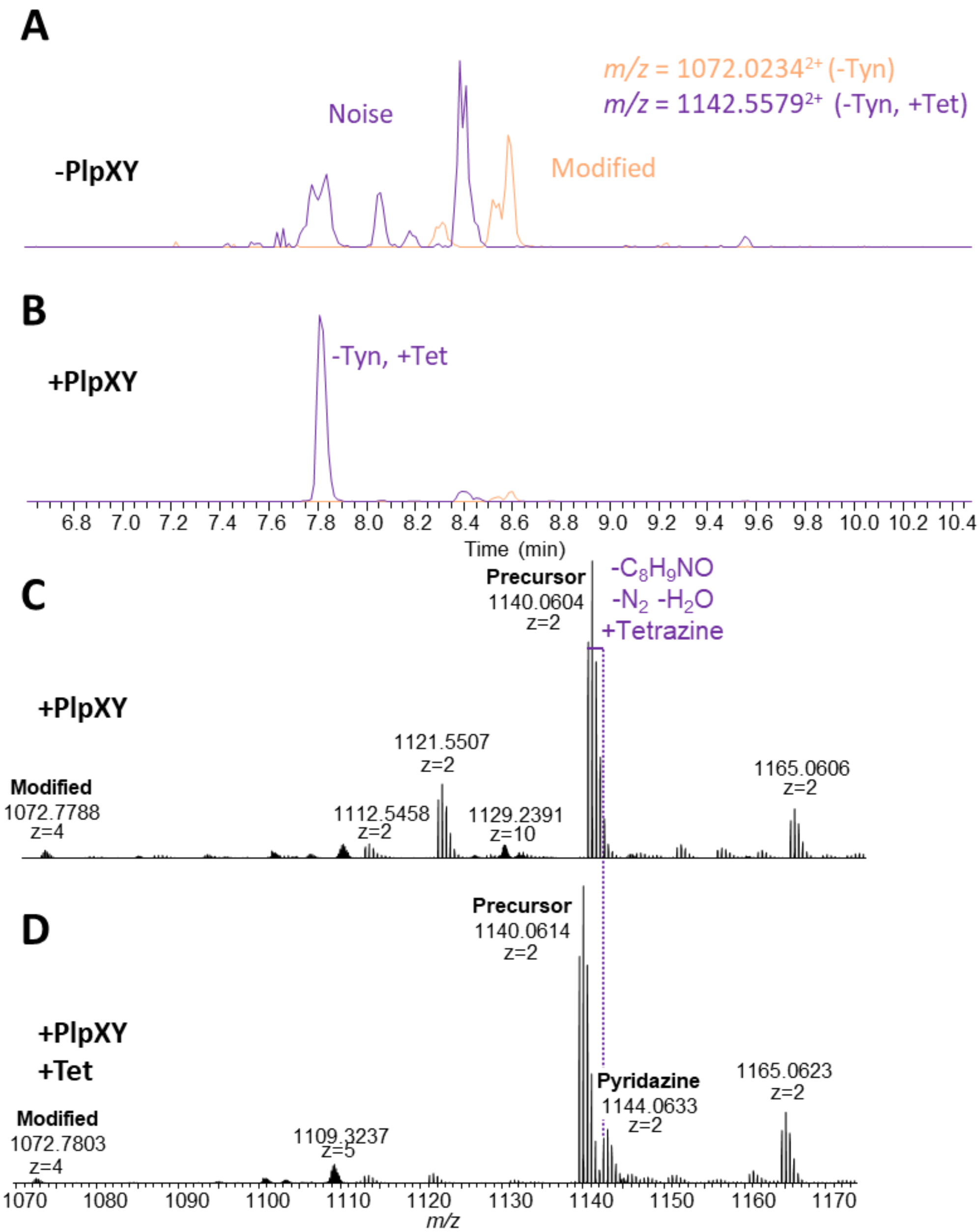

Supplementary Figure 29. LC-MS analysis for conjugation of 4-(1,2,4,5-tetrazin-3-yl)phenyl)methanamine **8** with His_6-PlpA3-M5G cleaved with trypsin to give the peptide fragment AVAAGYGVVFPWDNEFPWPR. Extracted ion chromatograms for *m/z* 1139.5576 (unmodified, $[M+2H]^{2+}$), *m/z* 1072.0234 (modified, $[M+2H]^{2+}$), and *m/z* 1142.5579 (pyridazine, $[M+2H]^{2+}$) for A) precursor + PlpXY co-expression and B) overnight incubation with tetrazine **2**. Extracted mass spectra for C) precursor + PlpXY co-expression and D) overnight incubation with 4-(1,2,4,5-tetrazin-3-yl)phenyl)methanamine **8**. "Modified" refers to excision of tyramine ($-C_8H_9NO$) and "pyridazine" to cycloaddition product formation.

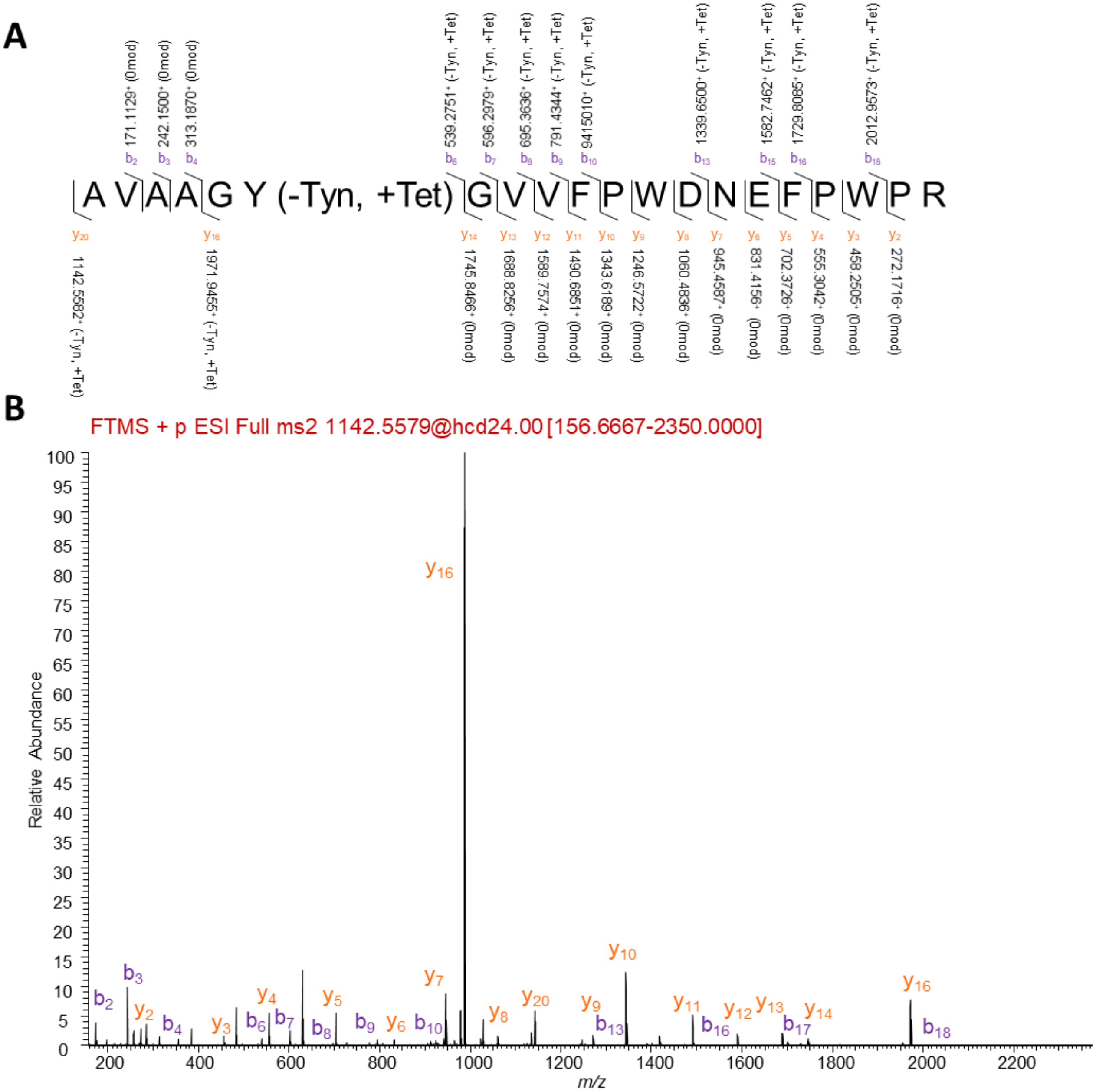

Supplementary Figure 30. Localization of pyridazine formation on His$_6$-PlpA3-M5G (with 4-(1,2,4,5-tetrazin-3-yl)phenyl)methanamine) cleaved with trypsin to give peptide fragment AVAAGYGVVFPWDNEFPWPR. A) Summary of observed b (above, purple) and y (below, orange) fragmentation ions. B) MS/MS spectrum from PRM-mediated fragmentation (CE 24) from *m/z* 1167.0555 ($[M+2H]^{2+}$) parent ion. Observed b and y ions are indicated as before.

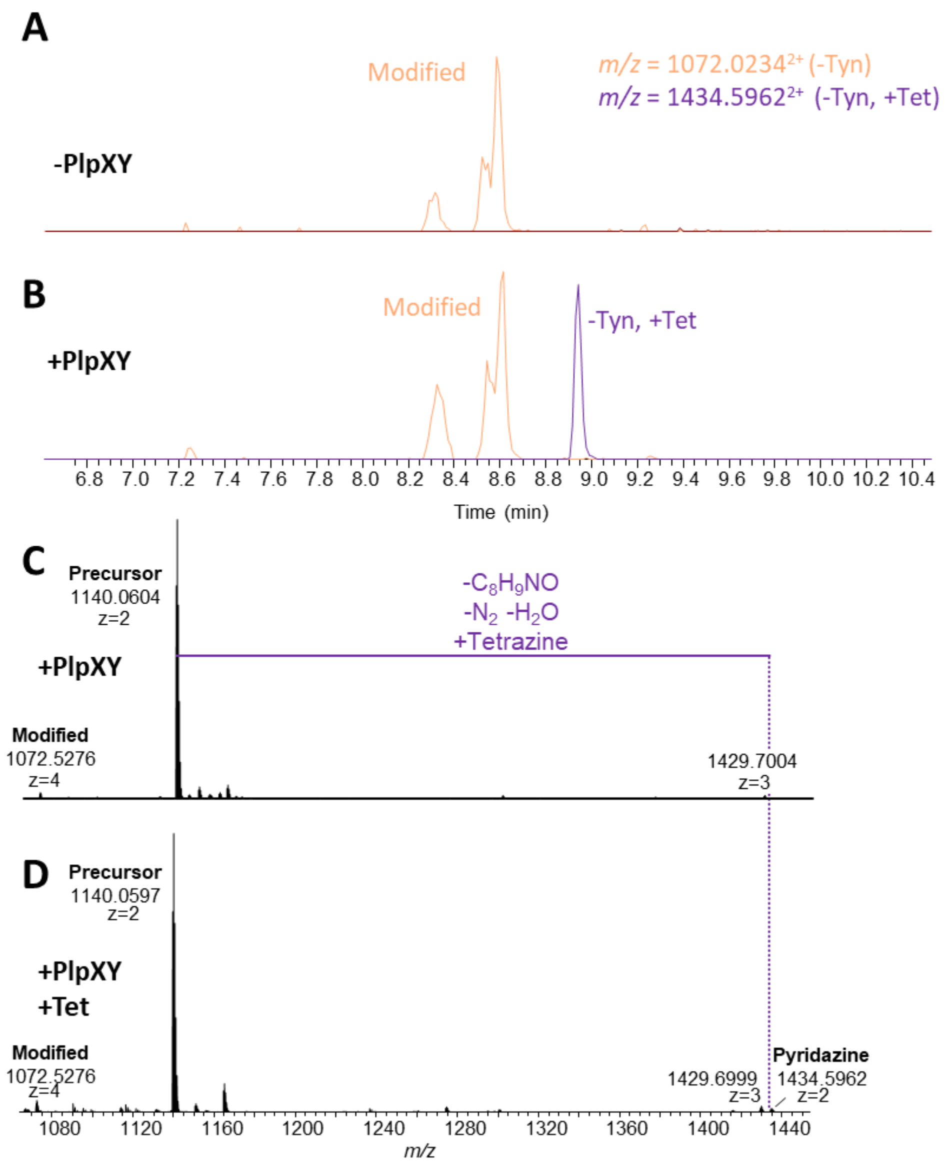

Supplementary Figure 31. LC-MS analysis for conjugation of CF®488A tetrazine **6** with His$_6$-PlpA3-M5G cleaved with trypsin to give the peptide fragment AVAAGYGVVFPWDNEFPWPR. Extracted ion chromatograms for *m/z* 1139.5576 (unmodified, $[M+2H]^{2+}$), *m/z* 1072.0234 (modified, $[M+2H]^{2+}$), and *m/z* 1434.5962 (pyridazine, $[M+2H]^{2+}$) for A) precursor + PlpXY co-expression and B) overnight incubation with tetrazine **2**. Extracted mass spectra for C) precursor + PlpXY co-expression and D) overnight incubation with CF488A® tetrazine **6**. "Modified" refers to excision of tyramine (-C_8H_9NO) and "pyridazine" to cycloaddition product formation.

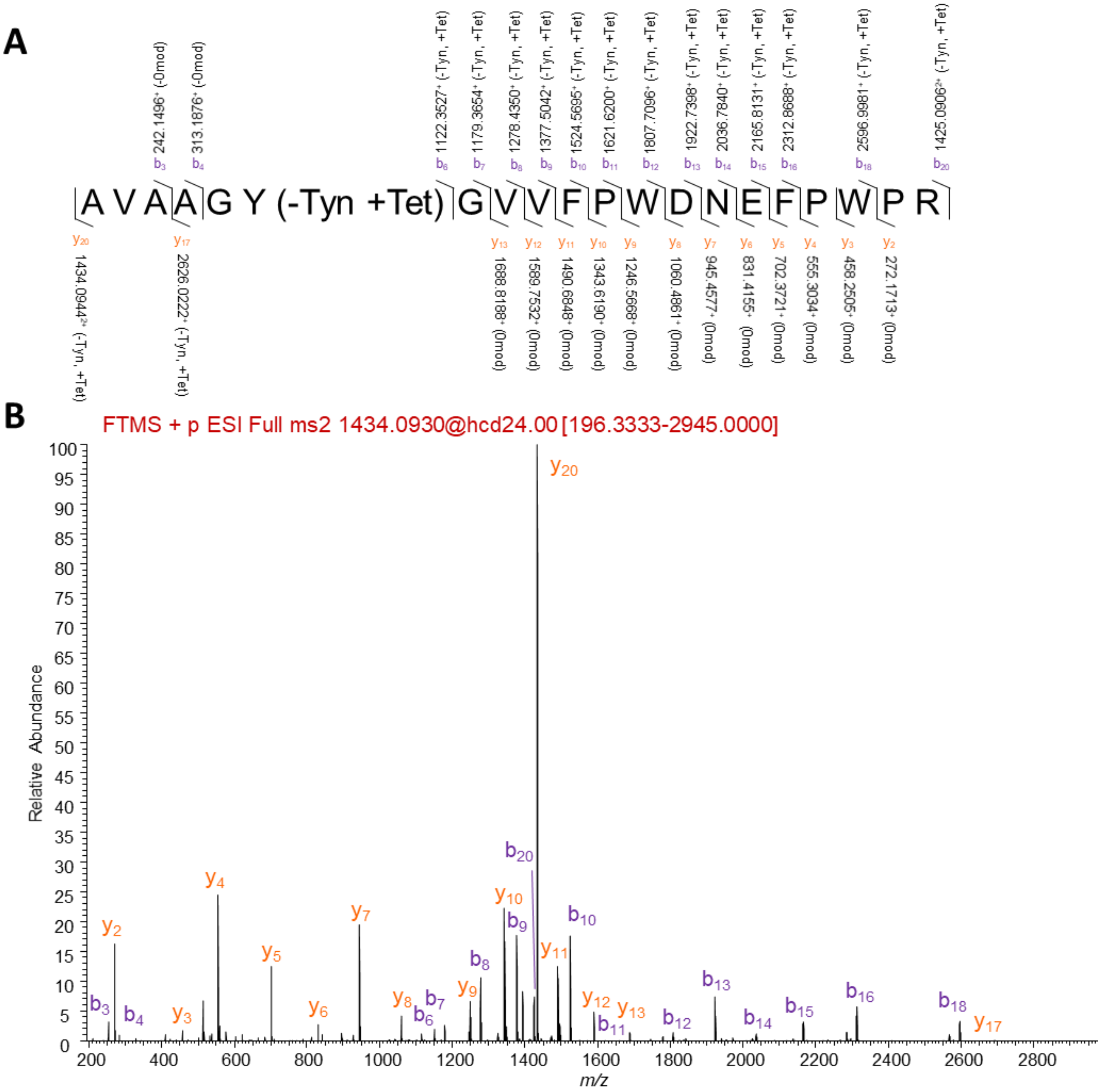

Supplementary Figure 32. Localization of pyridazine formation on His6-PlpA3-M5G (with CF®488A tetrazine **6**) cleaved with trypsin to give peptide fragment AVAAGYGVVFPWDNEFPWPR. A) Summary of observed b (above, purple) and y (below, orange) fragmentation ions. B) MS/MS spectrum from PRM-mediated fragmentation (CE 24) from *m/z* 1434.0930 ($[M+2H]^{2+}$) parent ion. Observed b and y ions are indicated as before.

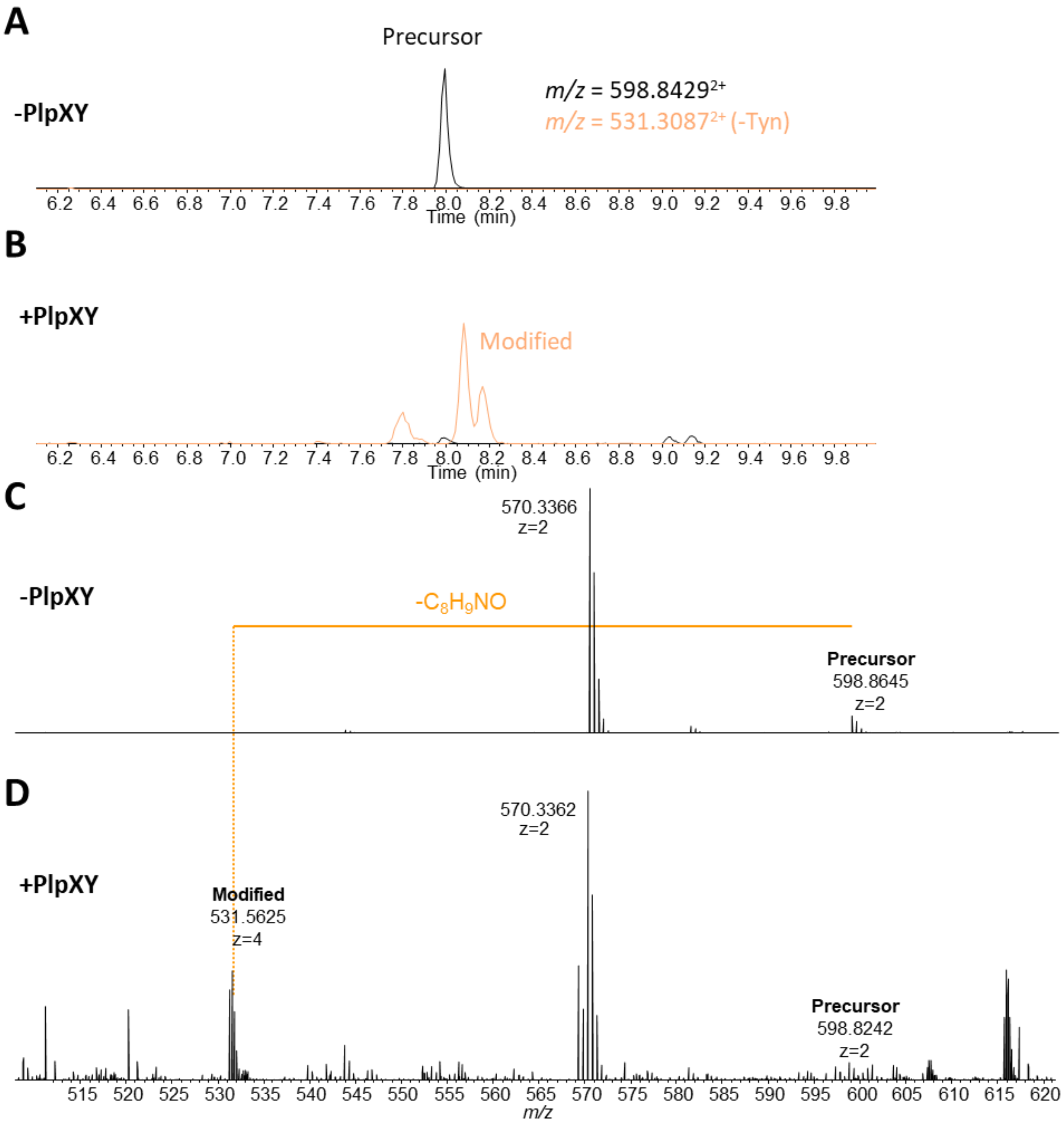

Supplementary Figure 33. LC-MS analysis for $Z_{Her2:342}$-GYG cleaved with trypsin to give the peptide fragment GGPIRPGYGLPI. Extracted ion chromatograms for *m*/*z* 598.8429 (unmodified, $[M+2H]^{2+}$), *m*/*z* 531.3087 (modified, $[M+2H]^{2+}$) for A) precursor only expression and B) precursor + PlpXY co-expression. Extracted mass spectra for C) precursor only expression and D) precursor + PlpXY coexpression. "Modified" refers to excision of tyramine (-C_8H_9NO). Conversion based on area under the curve for MS intensities is 83 %.

A

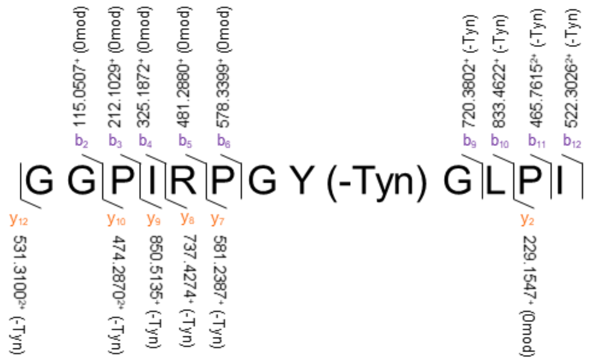

B

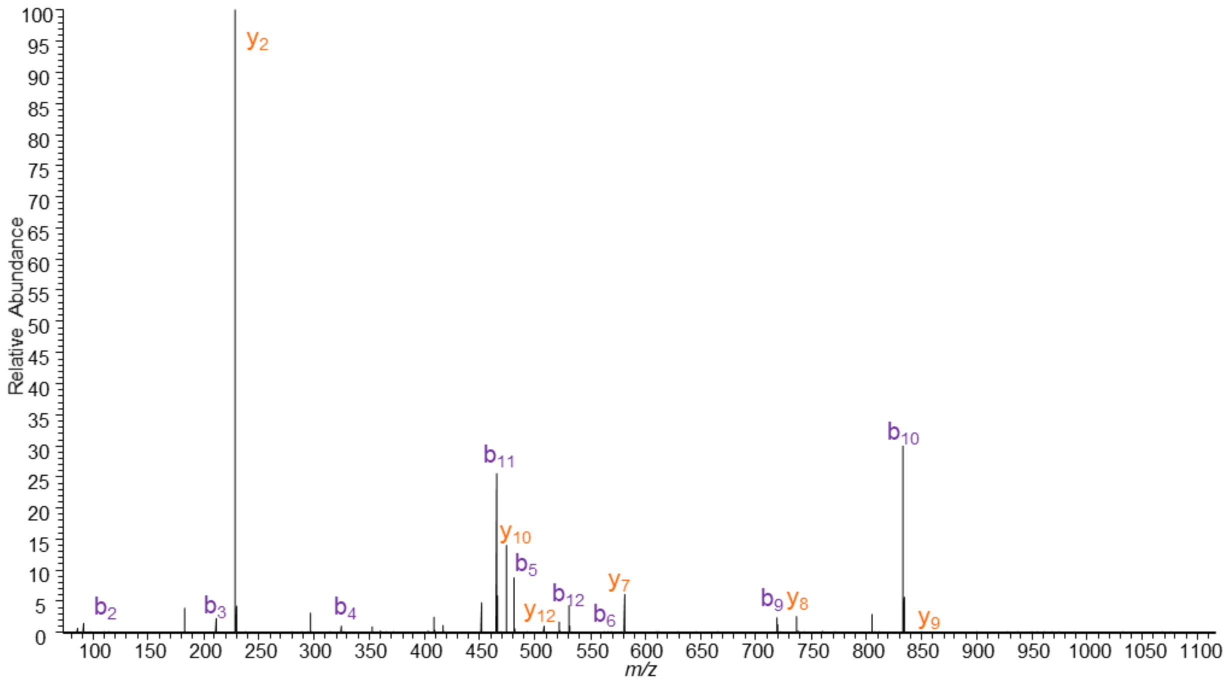

Supplementary Figure 34. Localization of C_8H_8NO loss from $Z_{Her2:342}$-GYG cleaved with trypsin to give peptide fragment GGPIRPGYGLPI. A) Summary of observed b (above, purple) and y (below, orange) fragmentation ions. B) MS/MS spectrum from PRM-mediated fragmentation (CE 21) from *m/z* 531.3087 ($[M+2H]^{2+}$) parent ion. Observed b and y ions are indicated as before.

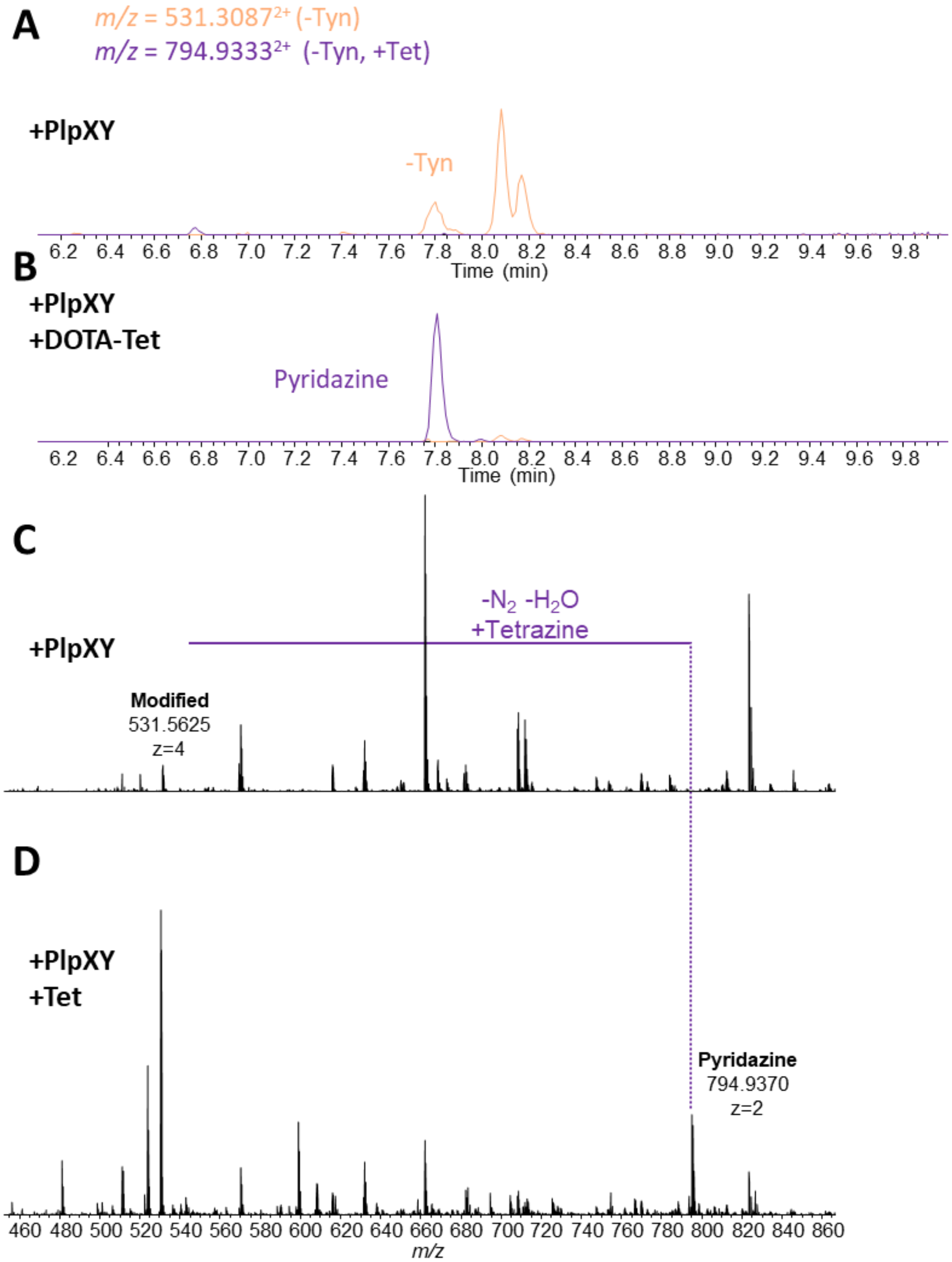

Supplementary Figure 35. LC-MS analysis for conjugation of DOTA-tetrazine **7** with $Z_{Her2:342}$-GYG cleaved with trypsin to give the peptide fragment GGPIRPGYGLPI. Extracted ion chromatograms for *m*/*z* 598.8429 (unmodified, $[M+2H]^{2+}$), *m*/*z* 531.3087 (modified, $[M+2H]^{2+}$), and *m*/*z* 794.9333 (pyridazine, $[M+2H]^{2+}$) for A) precursor + PlpXY co-expression and B) overnight incubation with tetrazine **7**. Extracted mass spectra for C) precursor + PlpXY co-expression and D) overnight incubation with DOTA-tetrazine **7**. "Modified"

refers to excision of tyramine (-C_8H_9NO) and "pyridazine" to cycloaddition product formation. Conversion based on area under the curve for MS intensities is 93 %.

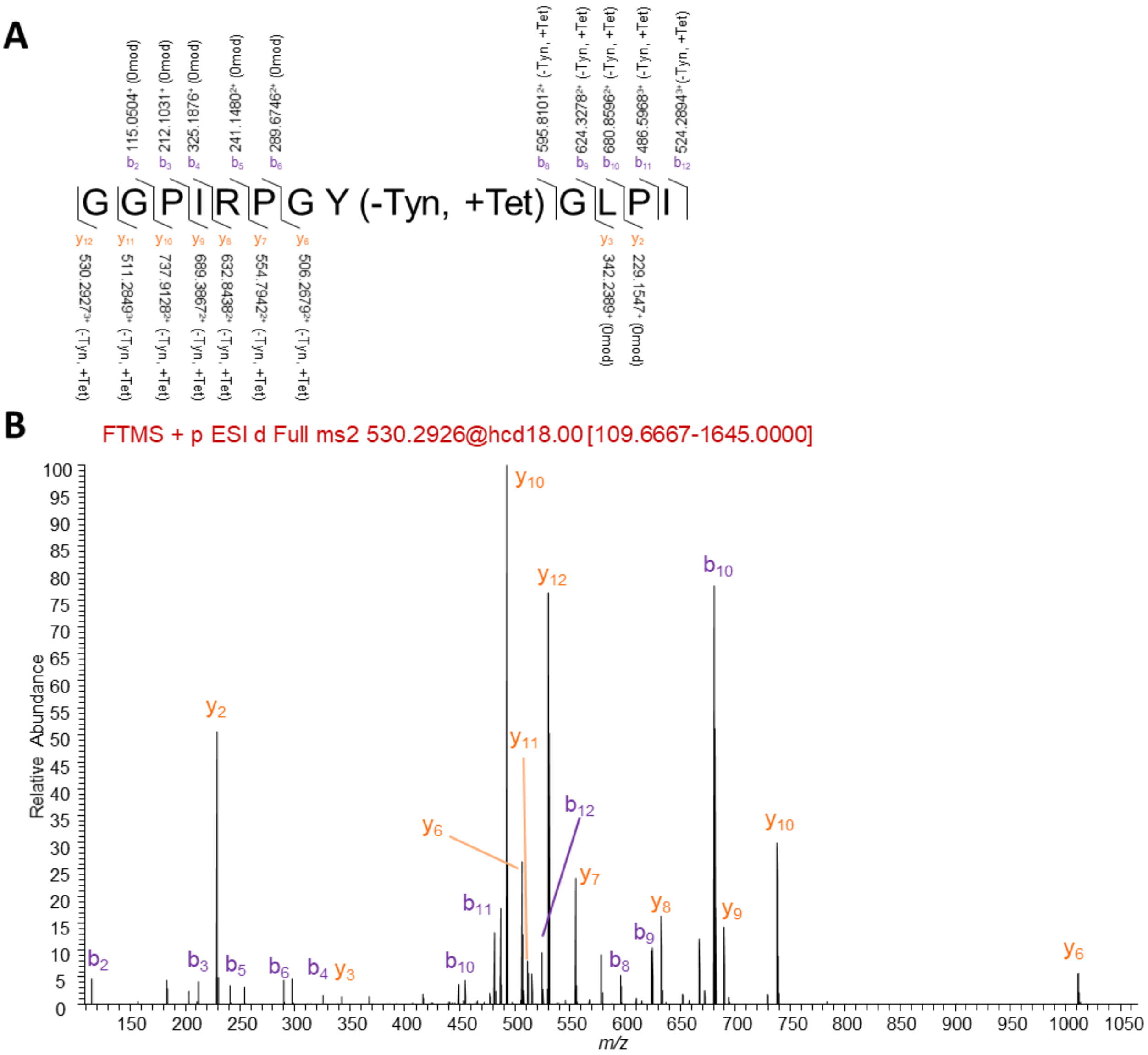

Supplementary Figure 36. Localization of pyridazine formation on $Z_{Her2:342}$-GYG (with DOTA-tetrazine **7**) cleaved with trypsin to give peptide fragment GGPIRPGYGLPI. A) Summary of observed b (above, purple) and y (below, orange) fragmentation ions. B) MS/MS spectrum from TOP10-mediated fragmentation (CE 18) from *m/z* 530.2926 ($[M+3H]^{3+}$) parent ion. Observed b and y ions are indicated as before.

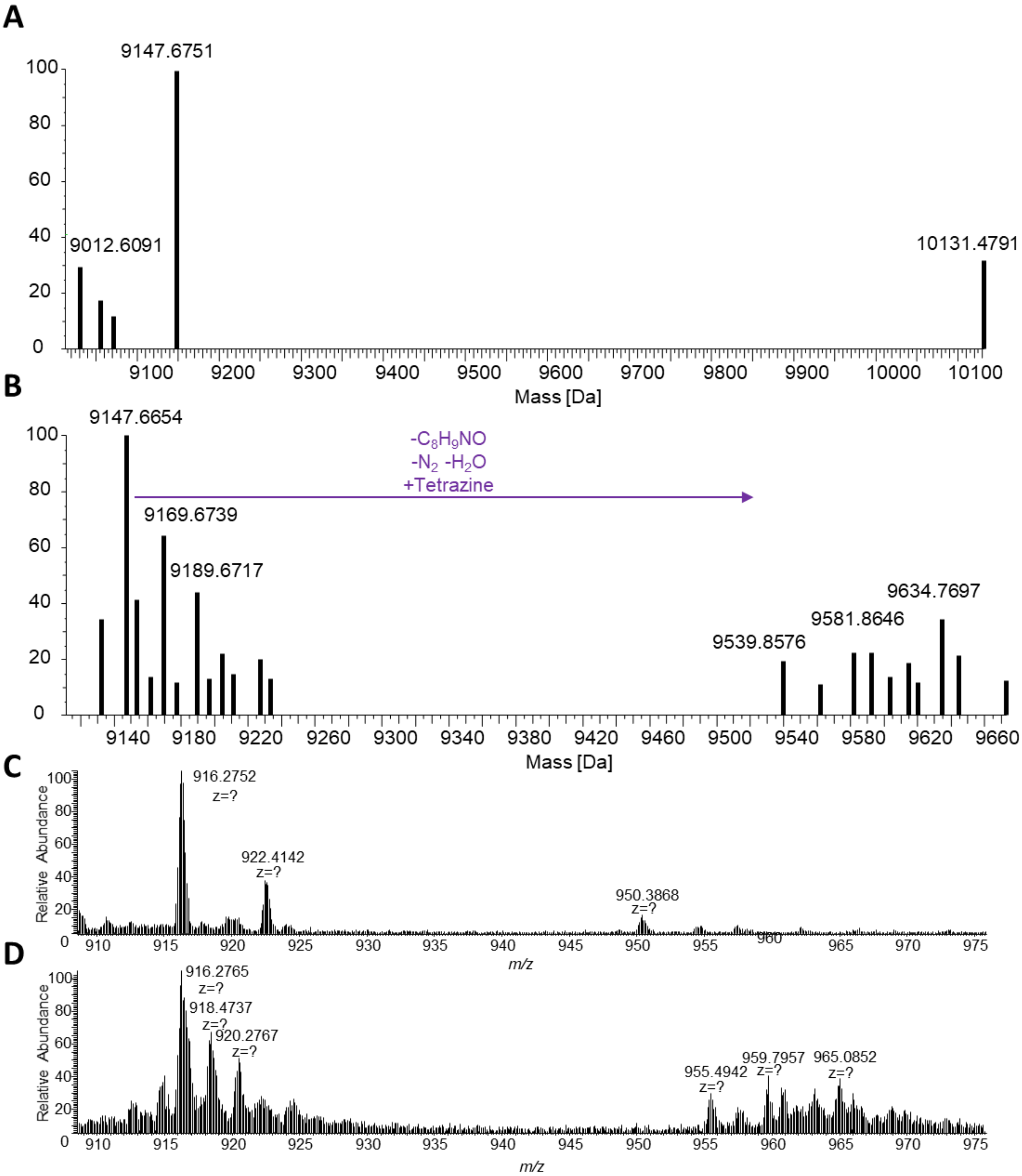

Supplementary Figure 37. LC-MS analysis of DOTA-tetrazine **7** with $Z_{Her2:342}$-GYG. Deconvoluted mass spectra for A) unconjugated (obs. 9147.6654 Da, calcd. 9147.6467 Da) and B) conjugated sample (obs. 9539.8576 Da, calcd. 9539.8275 Da), deconvoluted with ThermoFisher Freestyle 1.3. Raw MS for retention time 5.19-5.29 for C) unconjugated and D) conjugated protein used for deconvolution.

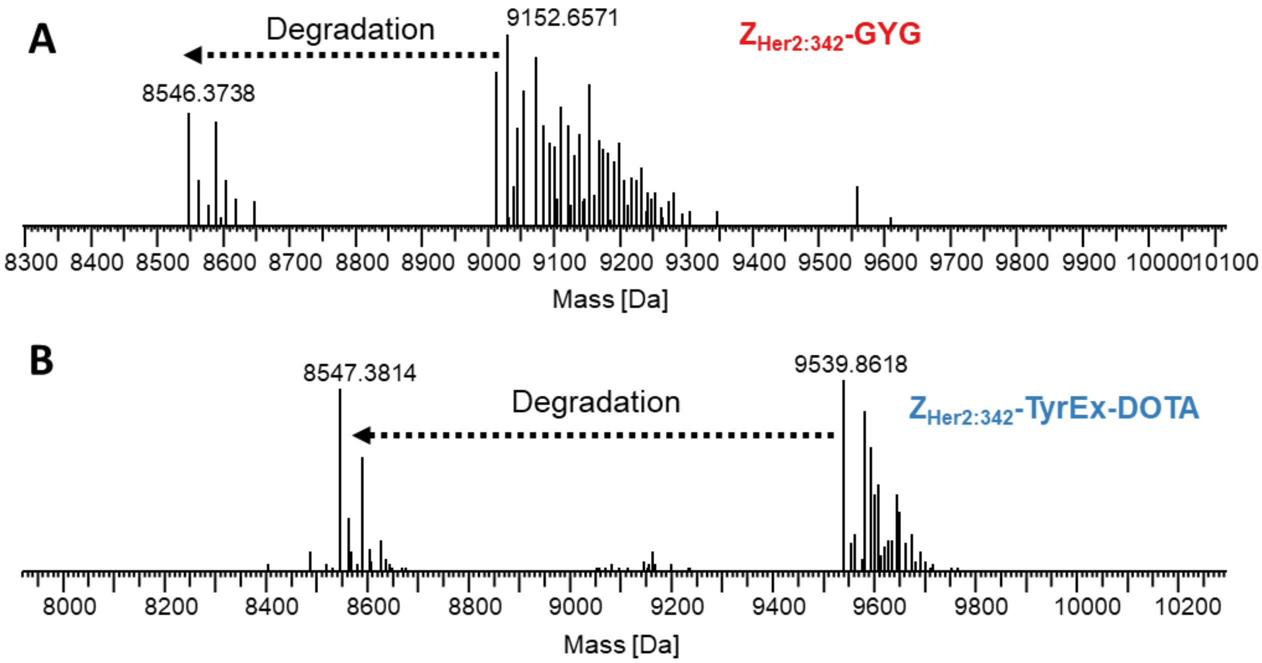

Supplementary Figure 38. LC-MS analysis of degradation of $Z_{Her2:342}$-GYG and $Z_{Her2:342}$-GYG-DOTA. Deconvoluted mass spectra for degradation product of A) unconjugated (obs. 8546.3738 Da) and B) conjugated sample (obs. 8547.3814 Da), deconvoluted with ThermoFisher Freestyle 1.3. Hydrolysis of the proline-glycine bond in the tag sequence GGPIRPGYGLPI leads to a product with mass of 8547.3196 Da. The masses suggest a C-terminal amide as the degradation product of $Z_{Her2:342}$-GYG.

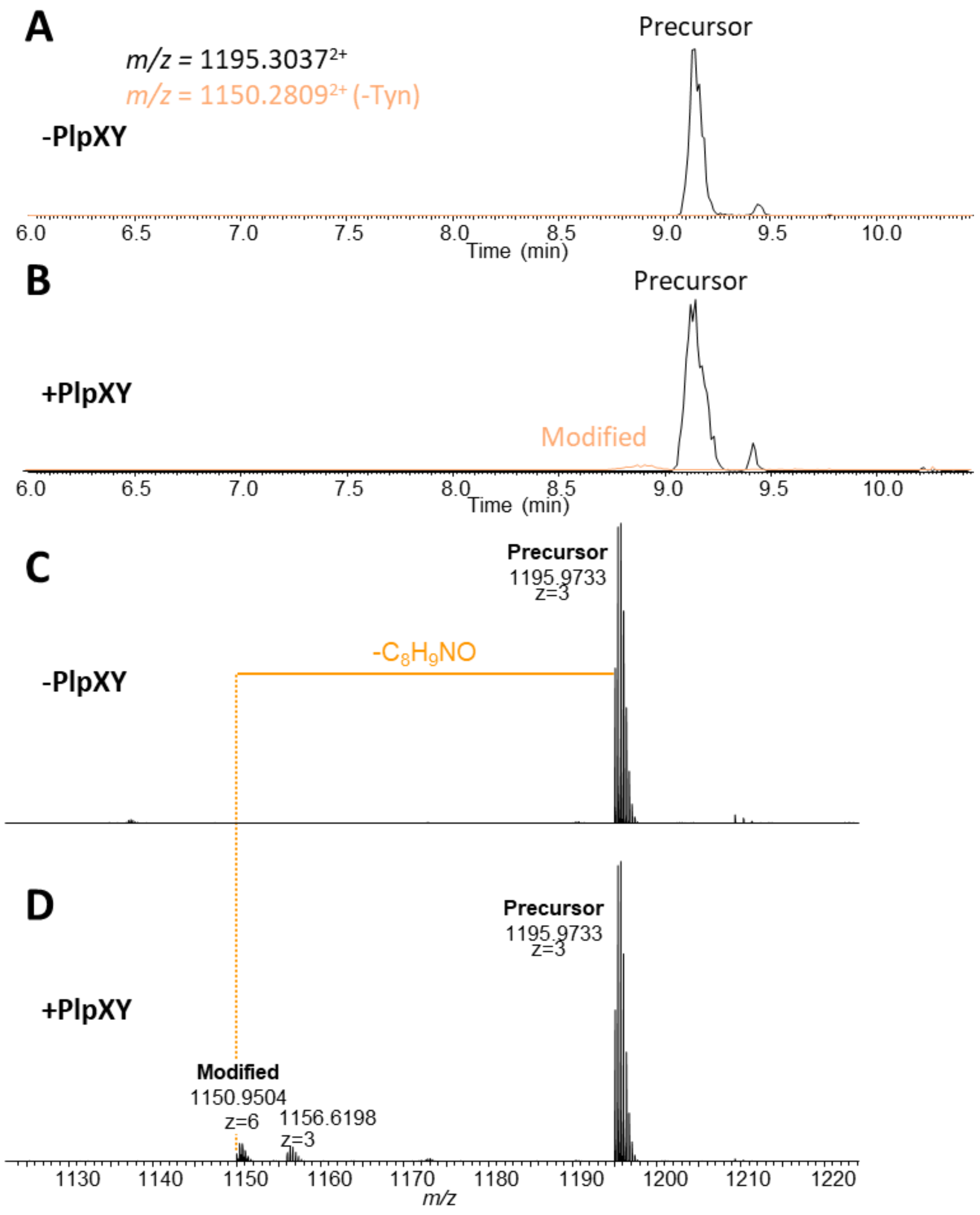

Supplementary Figure 39. LC-MS analysis for FtsZ-G55-Q56 cleaved with trypsin to give the peptide fragment TAGSTLEGGPIRPGYGLPIGGLEGSTQTIQIGSGITK. Extracted ion chromatograms for *m*/*z* 1195.3037 (unmodified, $[M+3H]^{3+}$), *m*/*z* 1150.2809 (modified, $[M+3H]^{3+}$) for A) precursor only expression and B) precursor + PlpXY co-expression. Extracted mass spectra for C) precursor only expression and D) precursor + PlpXY coexpression. "Modified" refers to excision of tyramine (-C_8H_9NO).

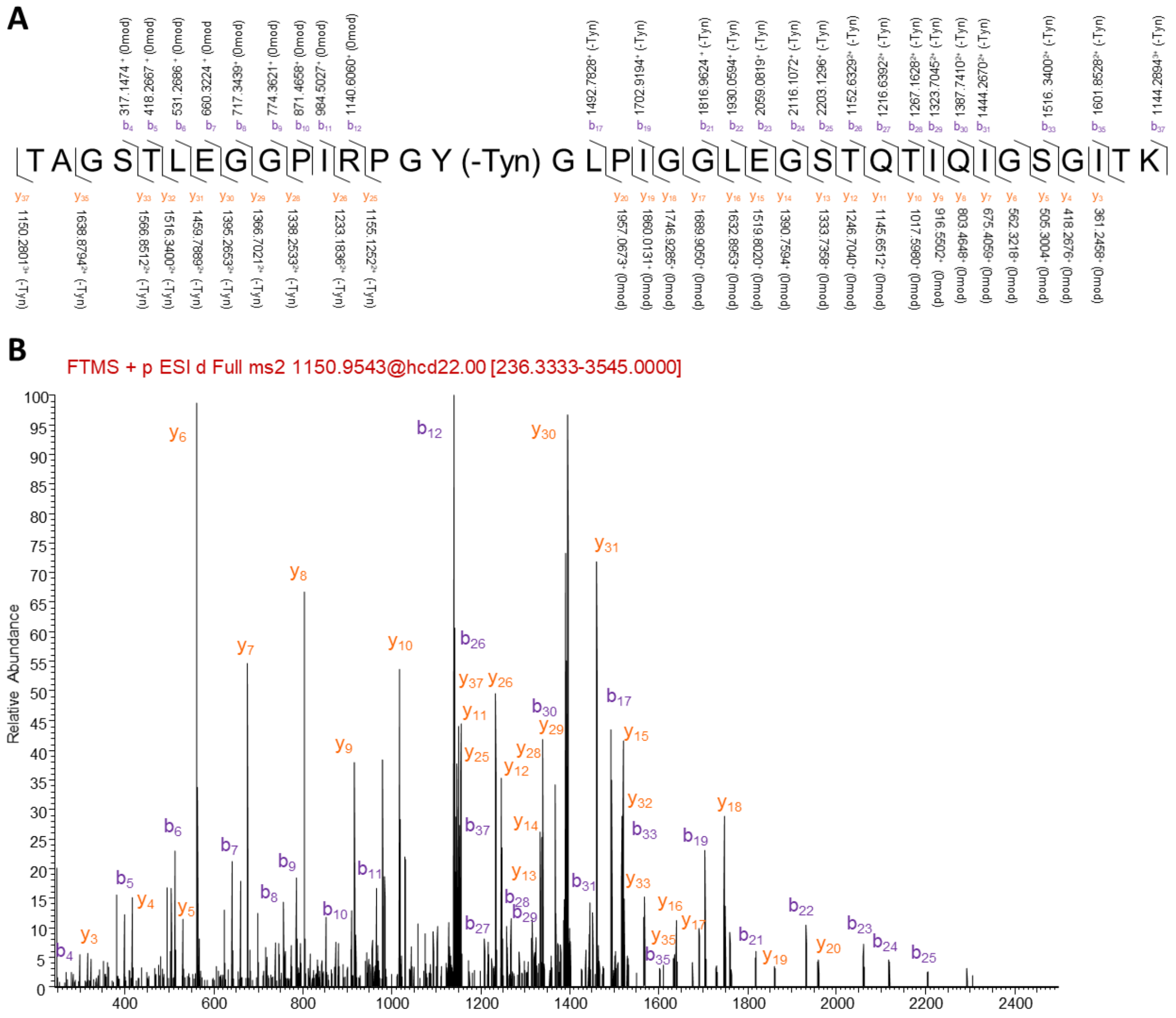

Supplementary Figure 40. Localization of C_8H_8NO loss from FtsZ-G55-Q56 cleaved with trypsin to give peptide fragment TAGSTLEGGPIRPGYGLPIGGLEGSTQTIQIGSGITK. A) Summary of observed b (above, purple) and y (below, orange) fragmentation ions. B) MS/MS spectrum from PRM-mediated fragmentation (CE 12) from *m/z* 1150.2809 ($[M+2H]^{3+}$) parent ion. Observed b and y ions are indicated as before.

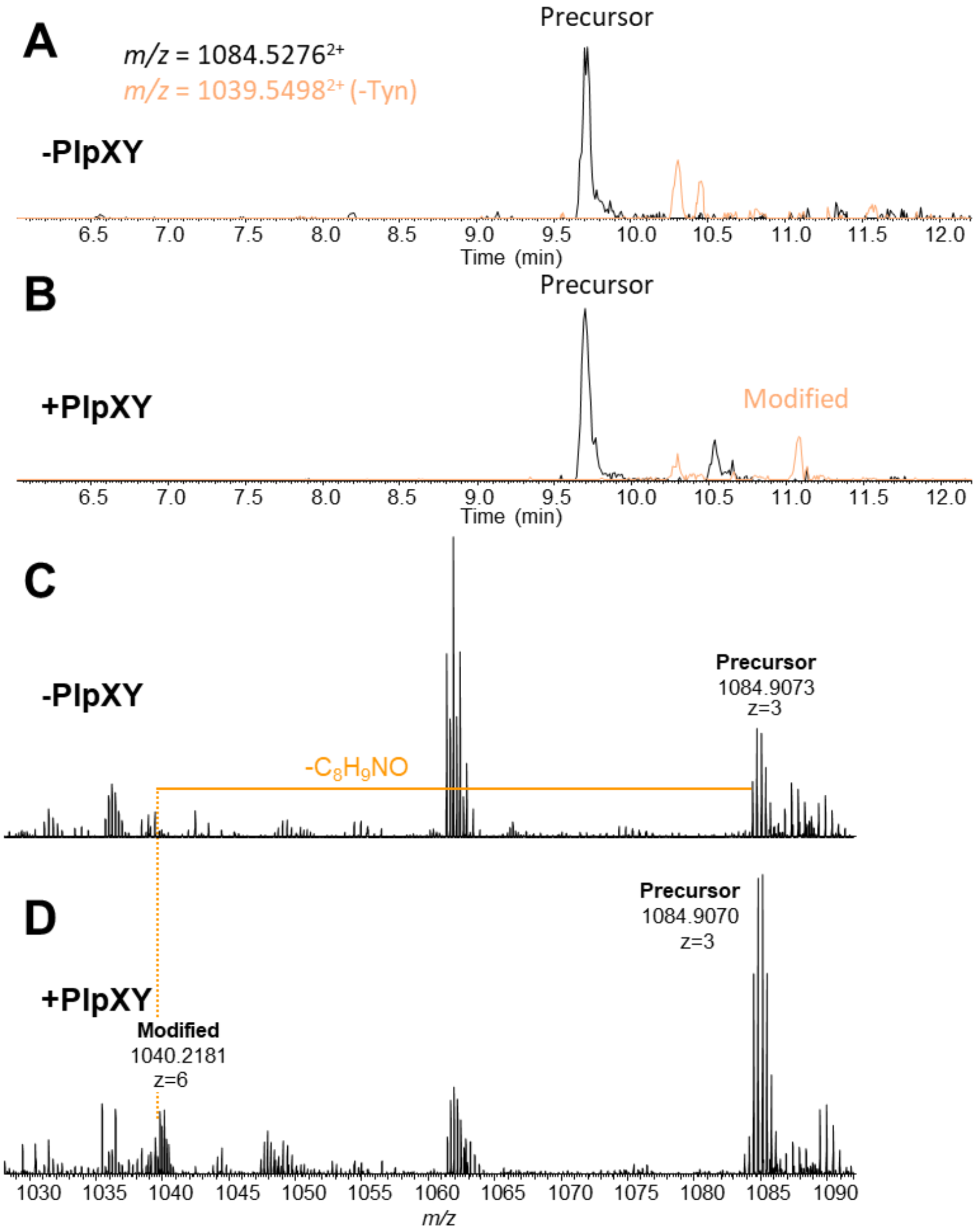

Supplementary Figure 41. LC-MS analysis for FtsZ-E350-Q364 cleaved with trypsin to give the peptide fragment YQQHGMAPLTQEPLFPIRPGYGLPIQTAK. Extracted ion chromatograms for *m/z* 1084.5276 (unmodified, $[M+3H]^{3+}$), *m/z* 1039.5498 (modified, $[M+3H]^{3+}$) for A) precursor only expression and B) precursor + PlpXY co-expression. Extracted mass spectra for C) precursor only expression and D) precursor + PlpXY coexpression. "Modified" refers to excision of tyramine ($-C_8H_9NO$).

A

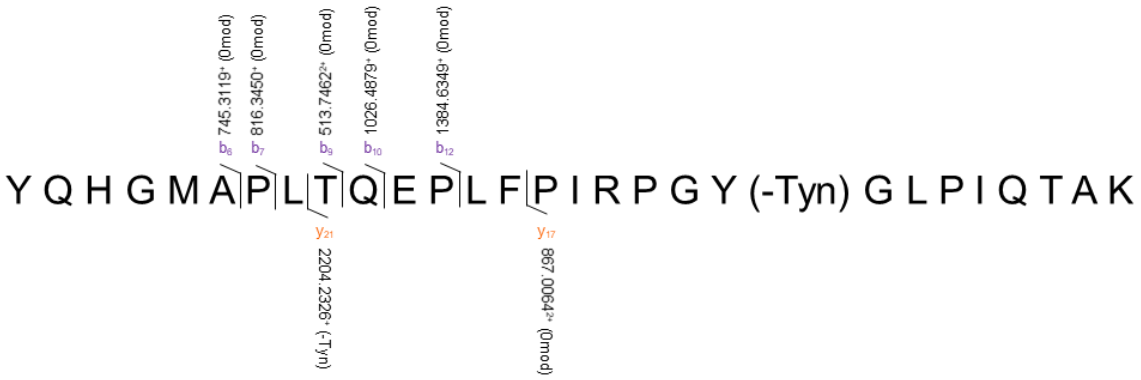

B

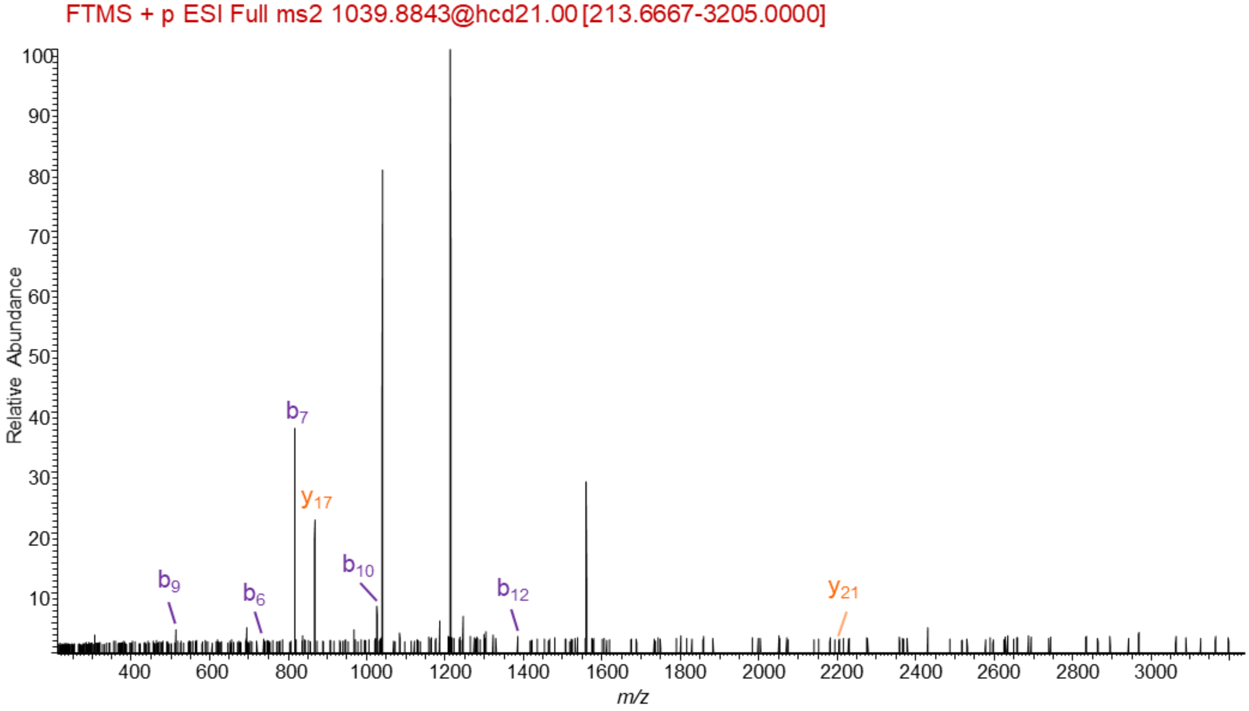

Supplementary Figure 42. Localization of C_8H_8NO loss from FtsZ-E350-Q364 cleaved with trypsin to give peptide fragment YQHGMAPLTQEPLFPIRPGYGLPIQTAK. A) Summary of observed b (above, purple) and y (below, orange) fragmentation ions. B) MS/MS spectrum from PRM-mediated fragmentation (CE 21) from *m/z* 1039.8843 ($[M+3H]^{3+}$) parent ion. Observed b and y ions are indicated as before.

Table S1. Primers used in the study

Name	Sequence (5’-3’)
FtsZ_iG55_F	CGGTCTGCCGATAGGAGGACAGACGATTCAAATCGGTAG
FtsZ_iG55_R	TATCCCGGGCGGATCGGTCCTCCAACCGCTGTTTTACG
FtsZ_iS218_F	TACGGTCTGCCGATAGGAGGAGAGATGGGCTACGCAATG
FtsZ_iS218_R	ACCCGGGCGGATCGGTCCTCCAGACATTACGGTGCGTAC
FtsZ_iE35_F	GGTTACGGTCTGCCGATAGGTGTTGAATTCTTCGCGGTAAATAC
FtsZ_iE35_R	CGGGCGGATCGGTCCTCCTTCAATGCGCTCGCGCAC
FtsZ_iNterm_F	TACGGTCTGCCCATAGGCGGCGACGCGGTGATTAAAGTC
FtsZ_iNterm_R	ACCCGGGCGGATAGGACCACCAAGTTCCATTGGTTCAAAC
FtsZ_iA53_F	GGATACGGTCTGCCGATAGTTGGACAGACGATTCAAATCGG
FtsZ_iA53_R	GGGCGGATCGGTCCTCCCGCTGTTTTACGCAGCGC
FtsZ-s325-D337_F	CTACGGTCTGCCCATAGATCGCTACCAGCAGCAT
FtsZ-s325-D337_R	CCCGGGCGGATGGGGAACAGAGTGATTTCAGGACGTTTG
FtsZ-s350-Q364_F	GGGTTATGGCTTGCCAATCCAAACTGCGAAAGAGCCGG
FtsZ-s350-Q364_R	GGTCTGATAGGGAACAAAGGCTCCTGGGTCAGCGGAGC

Table S1, cont.

FtsZ gene_fwd	ATCACCATCATCACCACAGCGGCAGCATGTTTGAACCAATG
FtsZ gene_rev	GGCGCGCCGAGCTCGAATTCTTAATCAGCTTGCTTACGCAG
pACYCDuet1_fwd	GAATTCGAGCTCGGCGCGCC
pACYCDuet1_rev	GCTGTGGTGATGATGGTGATGGC
Her2:342_UP	CAGCCATCACCATCATCACCACAGCCAGGTGGATAACAAATTTAACAAAG AAATGCGCAACGCGTATTGGGAAATTGCGCTGCTGCCGAACCTGAACAAC CAGCAGAAAAGAGCGTTTATTCGCAGCCTGTATGATGATCCGAGC
Her2:342_DOWN	CAGGCGCGCCGAGCTCGAATTCCTATATTGGTAAACCATAACCAGGTCTG ATAGGACCACCTTTCGGCGCCTGCGCATCGTTCAGTTTTTTCGCTTCCGC CAGCAGGTTCGCGCTCTGGCTCGGATCATCATACAGGCTGCGAATAAACG
Her2_Cys_FW	CTA GGA ATT CGA GCT CGG CG
Her2_Cys_RV	CAT TTC GGC GCC TGC GCA TC

Table S2. Plasmids used in the study

Plasmid	Description
plpA3/pACYCDuet-1	Encode the native PlpA3 precursor protein with an N-terminal His_6-tag (from here on referred to as His_6-PlpA3 (Morinaka, 2018)
plpXY/pRSFDuet	Encodes the Splicease PlpX together with its accessory protein PlpY under IPTG regulation and with kanamycin resistance (referred to as PlpXY) (Morinaka, 2018)
plpA3 M5G/pACYCDuet-1	PlpA3 protein with a point mutation at position 5 of the core peptide to glycine with an N-terminal His_6-tag (referred to as His_6-PlpA2-M5G)
ftsz/pRM006	FtsZ protein expression plasmid was a gift from Jie Xiao (Addgene plasmid # 98922)
ftsz G55/pRM006	FtsZ gene harbouring splicing motif insertion between G55-Q56
ftsz E350/pACYCDuet-1	FtsZ gene harbouring splicing motif substitution between E350-Q364
his_6-$z_{Her2:342}$-gyg /pACYCDuet-1	His_6-tagged $Z_{Her2:342}$ Affibody gene with C-terminal splicing motif
his_6-$z_{Her2:342}$-cys /pACYCDuet-1	His_6-tagged $Z_{Her2:342}$ Affibody gene with C-terminal reactive cysteine

Table S3. Protein sequences used in this study. Precursor (minimal) core sequence is underlined. Splicease modification sites in bold.

Protein	Sequence
Wild-type His$_6$-PlpA3	GSSHHHHHHSSGLVPRGSHMSIESAKAFYQRMTDDASFRTPFEAELSKEERQQLIKDSGYDFTAEEWQQAMTEIQAARSNEELNEEELEAIAGGAVAAM**YG**VVFPWDNEFPWPRWGG
His$_6$-PlpA3-M5G	GSSHHHHHHSSGLVPRGSHMSIESAKAFYQRMTDDASFRTPFEAELSKEERQQLIKDSGYDFTAEEWQQAMTEIQAARSNEELNEEELEAIAGGAVAA**GYG**VVFPWDNEFPWPRWGG
His$_6$-Z$_{Her2:342}$-GYG	GSSHHHHHHSQVDNKFNKEMRNAYWEIALLPNLNNQQKRAFIRSLYDDPSQSANLLAEAKKLNDAQAPKGGPIRPG**YG**LPI
His$_6$-Z$_{Her2:342}$-Cys	GSSHHHHHHSQVDNKFNKEMRNAYWEIALLPNLNNQQKRAFIRSLYDDPSQSANLLAEAKKLNDAQAPKGGC
E. coli FtsZ	MFEPMELTNDAVIKVIGVGGGGGNAVEHMVRERIEGVEFFAVNTDAQALRKTAVGQTIQIGSGITKGLGAGANPEVGRNAADEDRDALRAALEGADMVFIAAGMGGGTGTGAAPVVAEVAKDLGILTVAVVTKPFNFEGKKRMAFAEQGITELSKHVDSLITIPNDKLLKVLGRGISLLDAFGAANDVLKGAVQGIAELITRPGLMNVDFADVRTVMSEMGYAMMGSGVASGEDRAEEAAEMAISSPLLEDIDLSGARGVLVNITAGFDLRLDEFETVGNTIRAFASDNATVVIGTSLDPDMNDELRVTVVATGIGMDKRPEITLVTNKQVQQPVMDRYQQHGMAPLTQEQKPVAKVVNDNAPQTAKEPDYLDIPAFLRKQAD
