# Supplementary material for: Site-specific bioorthogonal protein labelling by tetrazine ligation using endogenous β-amino acid dienophiles
Source: Nat Chem. 2023 Jul 3;15(10):1422–30. doi: 10.1038/s41557-023-01252-8 (PMC10533398; doi:10.1038/s41557-023-01252-8)
Supplement: Supplementary file 2 — Reporting Summary [file 41557_2023_1252_MOESM2_ESM.pdf]

nature research

Corresponding author(s): Professor Jörn Piel

Last updated by author(s): Apr 25, 2023

Reporting Summary

Nature Research wishes to improve the reproducibility of the work that we publish. This form provides structure for consistency and transparency in reporting. For further information on Nature Research policies, see our Editorial Policies and the Editorial Policy Checklist.

Statistics

For all statistical analyses, confirm that the following items are present in the figure legend, table legend, main text, or Methods section.

n/a	Confirmed	
☐	☒	The exact sample size (*n*) for each experimental group/condition, given as a discrete number and unit of measurement
☐	☒	A statement on whether measurements were taken from distinct samples or whether the same sample was measured repeatedly
☐	☒	The statistical test(s) used AND whether they are one- or two-sided<br>*Only common tests should be described solely by name; describe more complex techniques in the Methods section.*
☒	☐	A description of all covariates tested
☒	☐	A description of any assumptions or corrections, such as tests of normality and adjustment for multiple comparisons
☐	☒	A full description of the statistical parameters including central tendency (e.g. means) or other basic estimates (e.g. regression coefficient) AND variation (e.g. standard deviation) or associated estimates of uncertainty (e.g. confidence intervals)
☐	☒	For null hypothesis testing, the test statistic (e.g. *F*, *t*, *r*) with confidence intervals, effect sizes, degrees of freedom and *P* value noted<br>*Give P values as exact values whenever suitable.*
☒	☐	For Bayesian analysis, information on the choice of priors and Markov chain Monte Carlo settings
☒	☐	For hierarchical and complex designs, identification of the appropriate level for tests and full reporting of outcomes
☒	☐	Estimates of effect sizes (e.g. Cohen's *d*, Pearson's *r*), indicating how they were calculated

Our web collection on statistics for biologists contains articles on many of the points above.

Software and code

Policy information about availability of computer code

Data collection	MS acquisition: Xcalibur 4.1, Chromeleon Xpress 7.2 (Thermo Scientific); NMR acquisition: TopSpin 3.5./4.1 (Bruker); Microscopy: Visiview (Visitron)
Data analysis	MS: Xcalibur 4.1 (Thermo Scientific); NMR: MNova 14 (Mestrelab Research); Assays: Microsoft Excel 2019 (Microsoft), Prism 9.2 (GraphPad Software), Inkscape 0.92 (Open-source); DNA and protein sequences: Geneious 7.1.9 (Biomatters Limited.); Microscopy: Fiji 1.51 (Open-source)

For manuscripts utilizing custom algorithms or software that are central to the research but not yet described in published literature, software must be made available to editors and reviewers. We strongly encourage code deposition in a community repository (e.g. GitHub). See the Nature Research guidelines for submitting code & software for further information.

Data

Policy information about availability of data

All manuscripts must include a data availability statement. This statement should provide the following information, where applicable:

- Accession codes, unique identifiers, or web links for publicly available datasets
- A list of figures that have associated raw data
- A description of any restrictions on data availability

The data supporting the findings of this study are available in this article and the Supplementary Information. The crystal structure of the affibody was fetched from the RCSB PDB (PDB 3MZW).

Field-specific reporting

Please select the one below that is the best fit for your research. If you are not sure, read the appropriate sections before making your selection.

☒ Life sciences ☐ Behavioural & social sciences ☐ Ecological, evolutionary & environmental sciences

For a reference copy of the document with all sections, see nature.com/documents/nr-reporting-summary-flat.pdf

Life sciences study design

All studies must disclose on these points even when the disclosure is negative.

Sample size	Biological test were performed in triplicates ; sample size was chosen based on limited amounts of compounds.
Data exclusions	In the conditions experiment for (Figure 1) two values were excluded as the MS machine reported an error and did not measure properly
Replication	Labling with the reaction was performed on multiple substrates, in multiple attempts to verify reproducibility. Small molecule studies were all repeated 3 times with high reproduciblity. ELISA measurements were done in duplicates. Stability measurements of the Affibody constructs were done in triplicates with high reproduciblity. Representative microscopy images were taken from 3-12 images acquired for each condition
Randomization	Randomization was not relevant for this study, as we did not perform group comparisons.
Blinding	No blinding was performed in our study, as no group comparisons were performed and there was no specific reason to expect bias, as MS/MS and NMR are unbiased techniques.

Reporting for specific materials, systems and methods

We require information from authors about some types of materials, experimental systems and methods used in many studies. Here, indicate whether each material, system or method listed is relevant to your study. If you are not sure if a list item applies to your research, read the appropriate section before selecting a response.

Materials & experimental systems

n/a	Involved in the study	
☐	☒	Antibodies
☒	☐	Eukaryotic cell lines
☒	☐	Palaeontology and archaeology
☒	☐	Animals and other organisms
☒	☐	Human research participants
☒	☐	Clinical data
☒	☐	Dual use research of concern

Methods

n/a	Involved in the study	
☒	☐	ChIP-seq
☒	☐	Flow cytometry
☒	☐	MRI-based neuroimaging

Antibodies

Antibodies used	Only commercially available antibodies were used in ELISA for affibodies: Goat Anti-Affibody IgG (Affibody SE, catalog Nr. 20.1000.01.0005, Batch 1304-PB011), Anti-goat IgG (Fc specific)-Peroxidase antibody (Sigma Aldrich, catalog number SAB3700259-2MG, Lot RI34052)
Validation	Validation statments of the antibodies were supplied with the commercially available antibodies. Anti-goat IgG (Fc specific)-Peroxidase antibody was tested by ELISA, Western Blotting and Immunohistochemistry.
