# Supplementary figures and images for: Site-specific bioorthogonal protein labelling by tetrazine ligation using endogenous β-amino acid dienophiles
Source: Nat Chem. 2023 Jul 3;15(10):1422–30. doi: 10.1038/s41557-023-01252-8 (PMC10533398; doi:10.1038/s41557-023-01252-8)

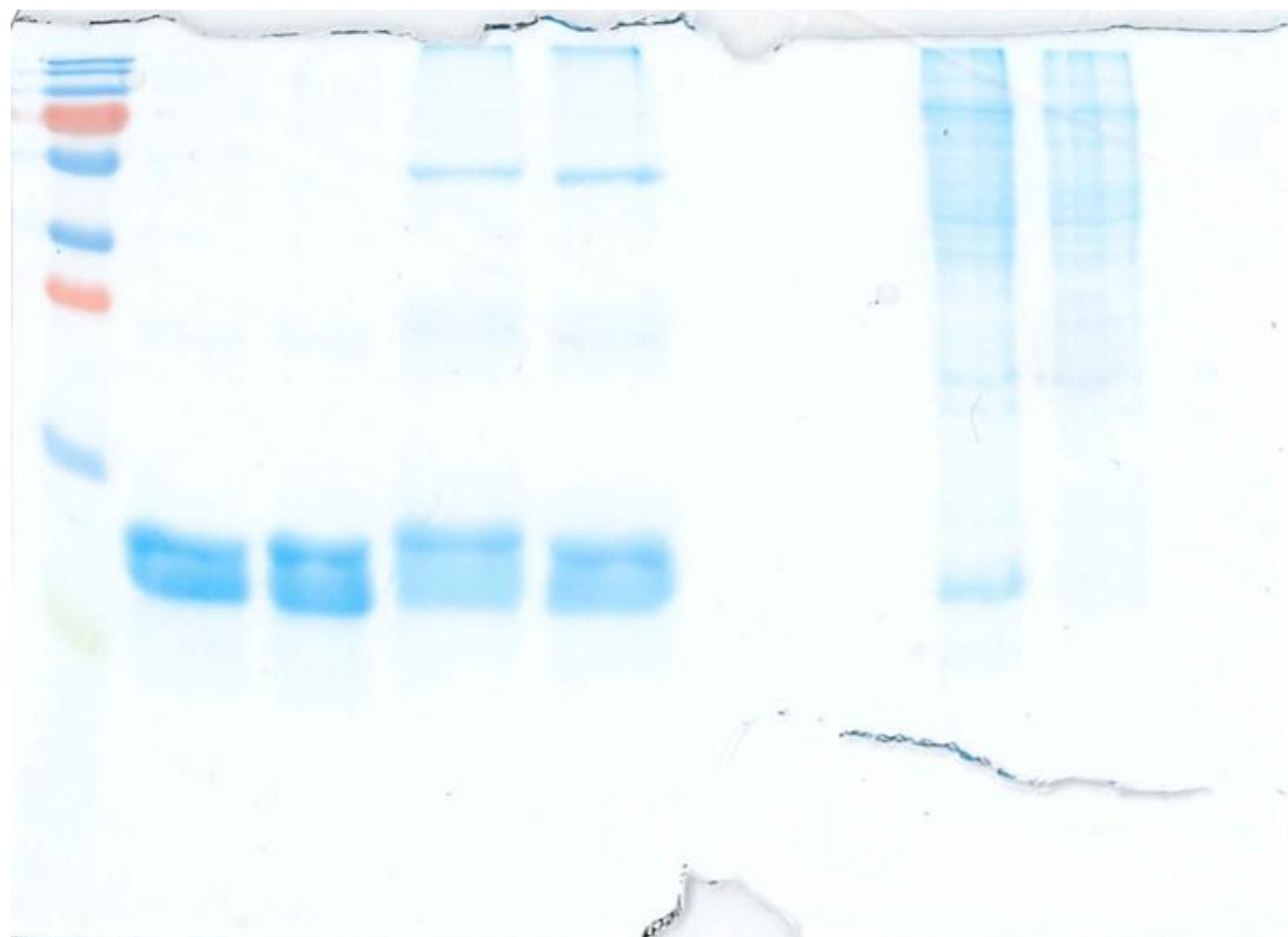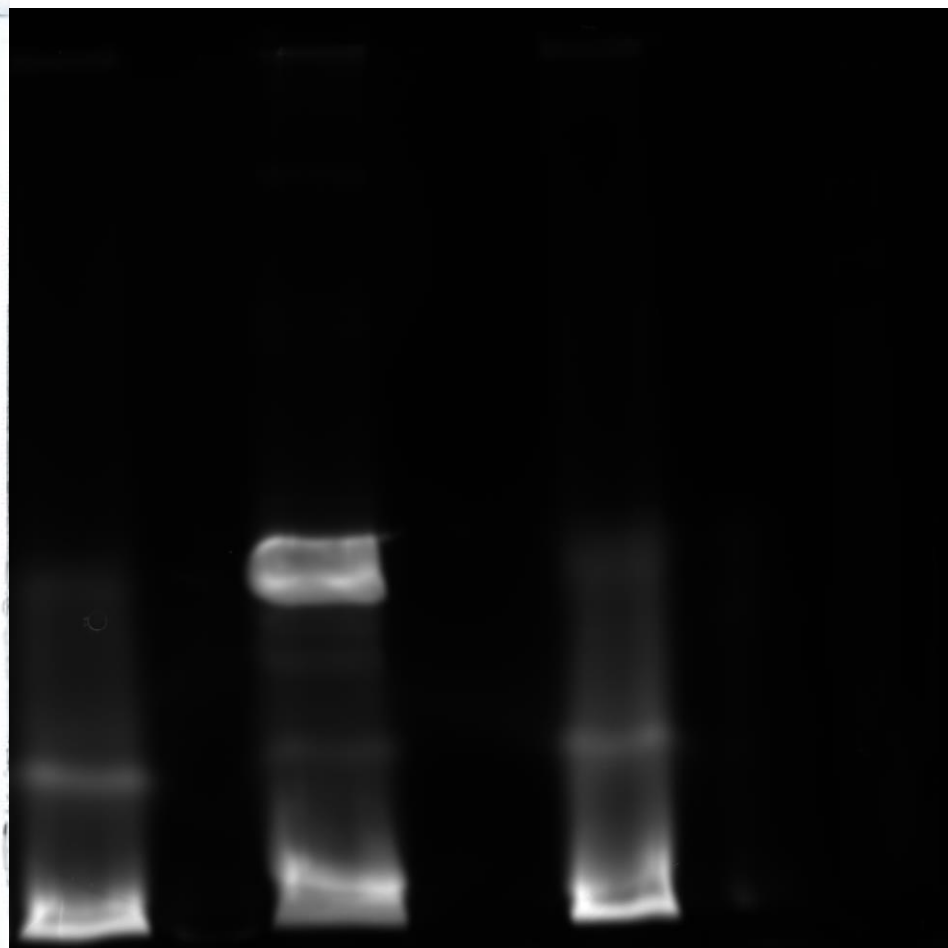

Supplement: Supplementary file 4 — Unprocessed gel scans. [file 41557_2023_1252_MOESM4_ESM.pdf]
