# Supplementary material for: Site-specific bioorthogonal protein labelling by tetrazine ligation using endogenous β-amino acid dienophiles
Source: Nat Chem. 2023 Jul 3;15(10):1422–30. doi: 10.1038/s41557-023-01252-8 (PMC10533398; doi:10.1038/s41557-023-01252-8)
Supplement: Supplementary file 5 — Unprocessed + cropped section of microscopy images, and unprocessed gel scans. [file 41557_2023_1252_MOESM5_ESM.pdf]

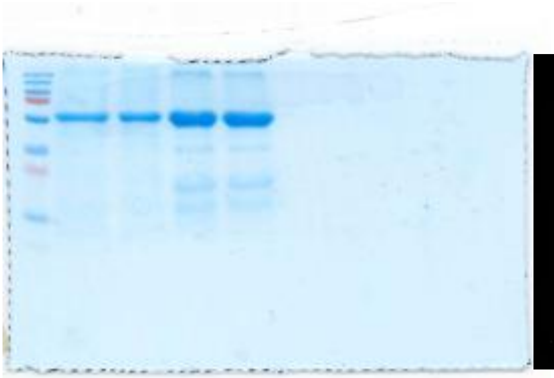
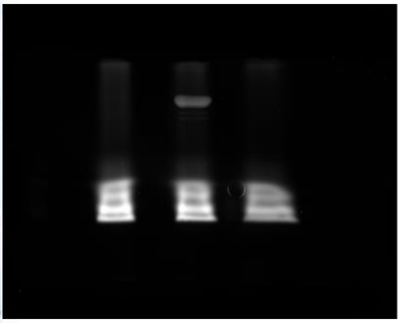

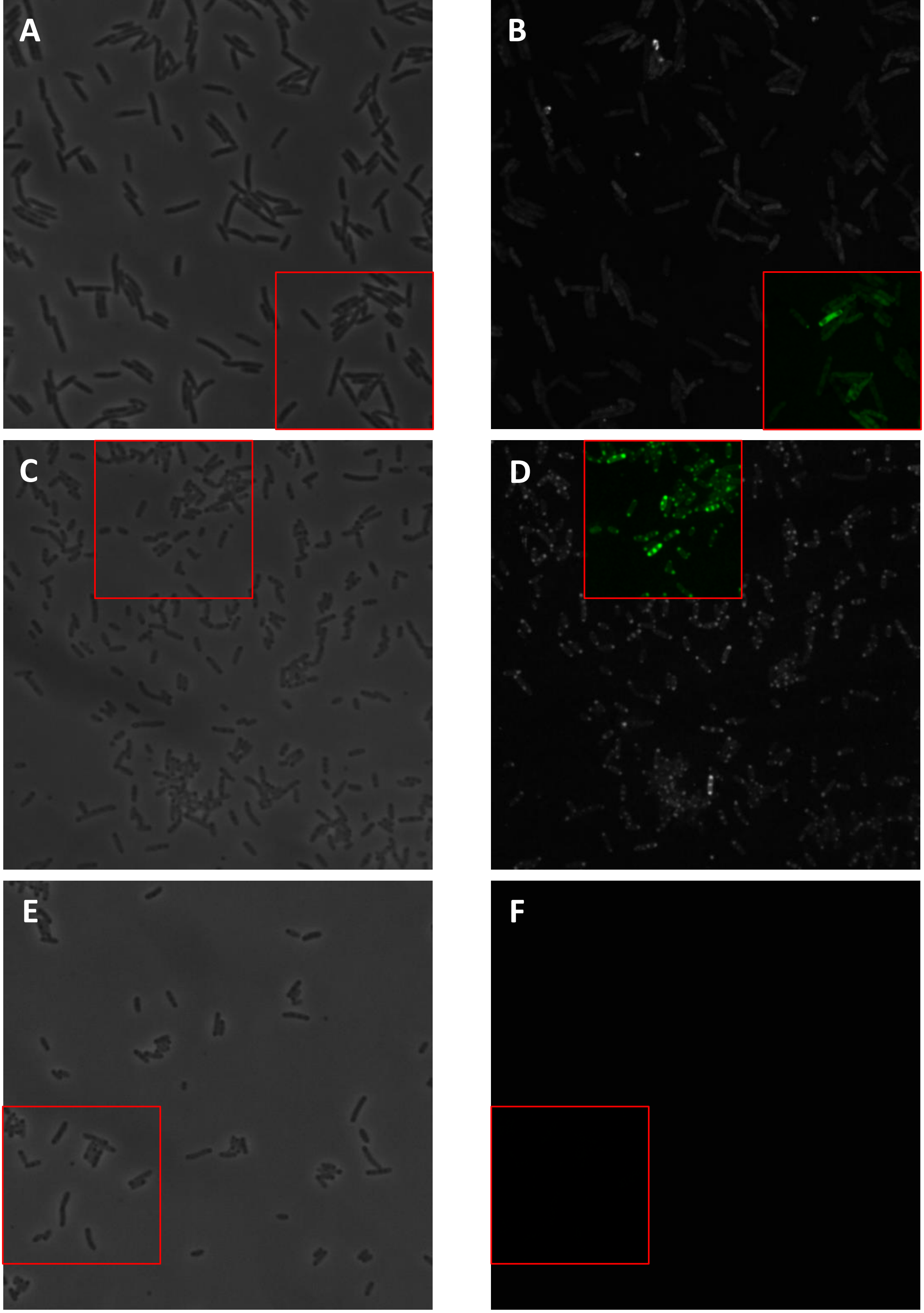

Full confocal microscopy images for FtsZ-G55 without (A,B) and with co-expression of PlpXY (C, D, E, F) in brightfield (A ,C, E) and GFP channel (B, D, F). Cells were stained with CF488A® tetrazine **6** (A,B,C,D). Cropped images used in the main text are illustrated with the red rectangle.

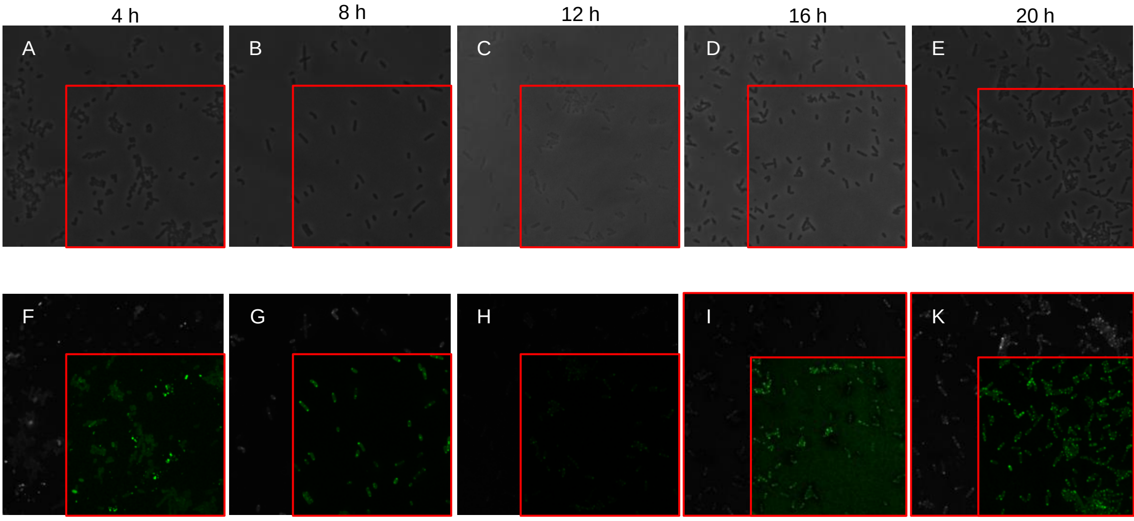

Full confocal microscopy images for FtsZ-G55 with co-expression of PlpXY in brightfield (A, B, C, D, E) and GFP channel (F, G, H, I, K). Cells were stained with CF488A® tetrazine **6**. Cropped images used in the main text are illustrated with the red rectangle.
